# Stratification of clear cell renal cell carcinoma (ccRCC) genomes by gene-directed copy number alteration (CNA) analysis

**DOI:** 10.1371/journal.pone.0176659

**Published:** 2017-05-09

**Authors:** H.-J. Thiesen, F. Steinbeck, M. Maruschke, D. Koczan, B. Ziems, O. W. Hakenberg

**Affiliations:** 1 Institute of Immunology, University of Rostock, Rostock, Germany; 2 Department of Urology, University of Rostock, Rostock, Germany; 3 Department of Urology, HELIOS Hanseklinikum Stralsund, Germany; 4 Steinbeis Center for Proteome Analysis, Rostock, Germany; Universitat Zurich, SWITZERLAND

## Abstract

Tumorigenic processes are understood to be driven by epi-/genetic and genomic alterations from single point mutations to chromosomal alterations such as insertions and deletions of nucleotides up to gains and losses of large chromosomal fragments including products of chromosomal rearrangements e.g. fusion genes and proteins. Overall comparisons of copy number alterations (CNAs) presented in 48 clear cell renal cell carcinoma (ccRCC) genomes resulted in ratios of gene losses versus gene gains between 26 ccRCC Fuhrman malignancy grades G1 (ratio 1.25) and 20 G3 (ratio 0.58). Gene losses and gains of 15762 CNA genes were mapped to 795 chromosomal cytoband loci including 280 KEGG pathways. CNAs were classified according to their contribution to Fuhrman tumour gradings G1 and G3. Gene gains and losses turned out to be highly structured processes in ccRCC genomes enabling the subclassification and stratification of ccRCC tumours in a genome-wide manner. CNAs of ccRCC seem to start with common tumour related gene losses flanked by CNAs specifying Fuhrman grade G1 losses and CNA gains favouring grade G3 tumours. The appearance of recurrent CNA signatures implies the presence of causal mechanisms most likely implicated in the pathogenesis and disease-outcome of ccRCC tumours distinguishing lower from higher malignant tumours. The diagnostic quality of initial 201 genes (108 genes supporting G1 and 93 genes G3 phenotypes) has been successfully validated on published Swiss data (GSE19949) leading to a restricted CNA gene set of 171 CNA genes of which 85 genes favour Fuhrman grade G1 and 86 genes Fuhrman grade G3. Regarding these gene sets overall survival decreased with the number of G3 related gene losses plus G3 related gene gains. CNA gene sets presented define an entry to a gene-directed and pathway-related functional understanding of ongoing copy number alterations within and between individual ccRCC tumours leading to CNA genes of prognostic and predictive value.

## Introduction

Tumourigenic processes driven by epi-/genetic and genomic alterations consist of an interplay of individual events from single point mutations to chromosomal alterations such as insertions and deletions of nucleotides up to gains and losses of large chromosomal fragments including products of chromosomal rearrangements e.g. fusion genes and proteins [1–5]. All these processes specify genetic heterogeneities within tumour tissues and contribute to the malignancy of individual tumour sub-/types [6–10]. Renal cell carcinoma is the most common malignancy of the adult kidney with an increasing incidence over the last years [11] reaching 2–3% of all malignancies worldwide [12]. The most frequent histomorphological subtype that originates from renal parenchyma is clear cell renal cell carcinoma (ccRCC) accounting for 70–80% of all malignancies [13, 14]. Surgical removal of the affected kidney by complete or partial nephrectomy is considered the primary treatment [15, 16]. Currently, even earlier tumour stages are reached due to widespread use of high resolution kidney imaging techniques [17, 18]. Thus, renal tumour tissues are accessible for histological staging / grading and in-depth genetic analysis [19–21]. The pathogenesis of ccRCC has been shown to be closely connected with common genetic alterations at particular chromosomal regions [22–25]. Deletions and unbalanced translocations of chromosome 3p are the most frequent abnormalities associated with chromosomal loss of specific regions, involving among others the *VHL* gene locus [26, 27]. *VHL* gene inactivation occurs in more than approximately 60% of sporadic RCC through a gene mutation (33% to 66% of cases) or less commonly through promoter methylation (5% to 19%) [28]. Losses and gains of certain gene segments in RCC tumour tissues are suspected to interfere with gene functionalities such as transcriptional gene expression and patient outcome [29, 30]. Loss of the remaining *VHL* allele (loss of heterozygosity) leads to a decrease in functional *VHL* protein and, subsequently, to the induction of hypoxia regulated genes [31]. Recent studies of gene expression levels in haploid and diploid chromosomal regions in HAP1 cells substantiate the relevance and the impact of gene losses and gains on the transcriptional level. In the HAP1 cell system, expression levels of an originally diploid chromosomal region have recently been shown to be reduced by half after the diploid region has become haploid by CRISPR-Cas9 engineering [32]. Thus, ongoing search and characterization of robust nominators describing ccRCC subtypes are considered instrumental in elucidating individual steps driving tumour initiation and progression [33–34]. Recent CNA studies supported by exome and whole genome studies underscore the presence of huge tumour heterogeneities within individual tumour samples [35] leading to cancer trunk-branch [36] and river models [37] of mutational cancer evolution.

**Fig 1:**
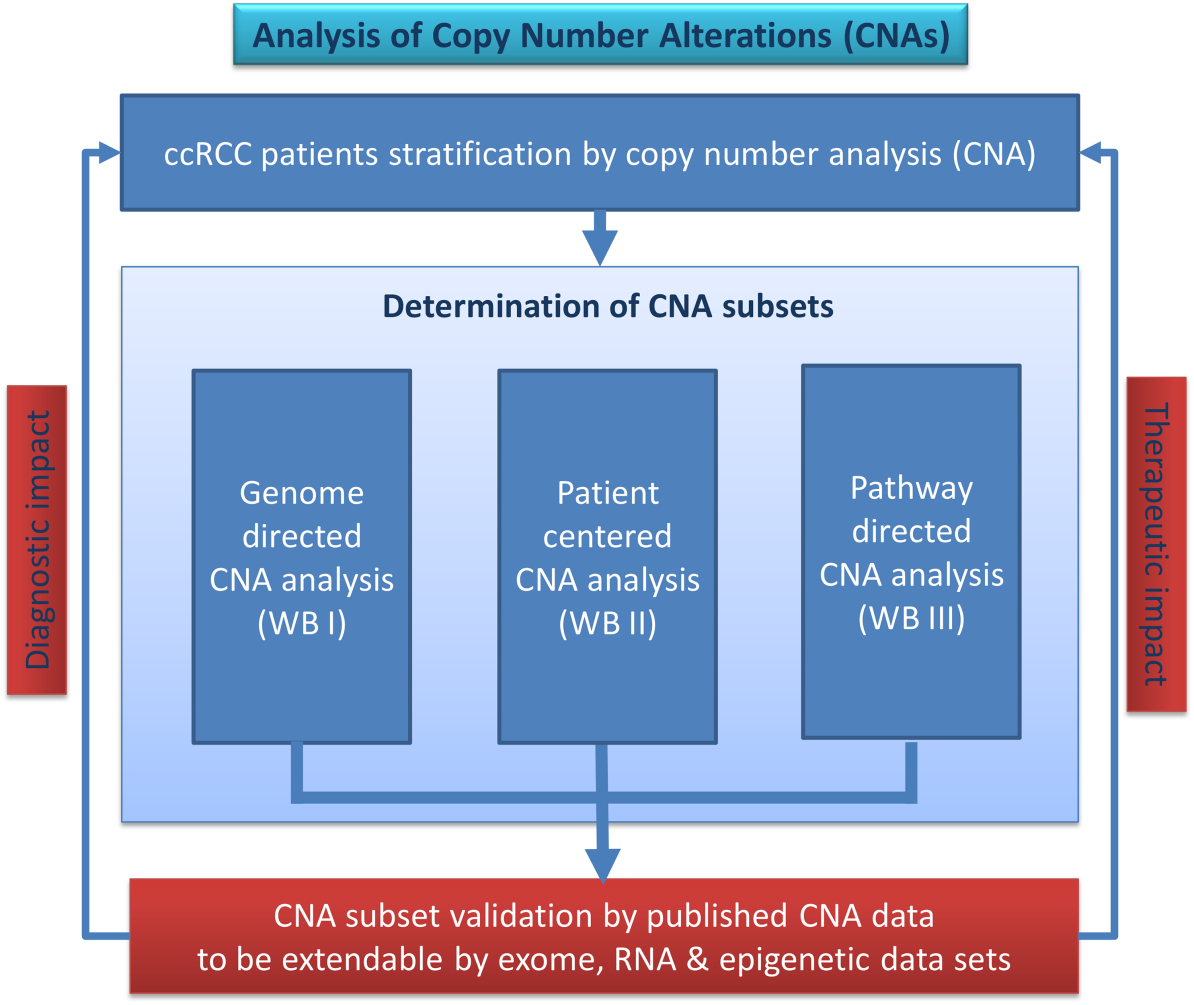
Workflow “analysis of copy number alterations (CNAs)”. Three different workflow branches (WB I–III) conducting CNA analysis have been applied leading to CNA gene sets that were validated by published and external data.

The roadmap and workflow of the copy number analysis performed at the University Medicine of the Hansestadt Rostock (HRO) stratifies gene losses and gains in clear cell renal cell carcinoma (ccRCC) tumours. Fuhrman grade G1 (26 HRO tumour samples) have been distinguished from Fuhrman grade G3 (20 HRO tumour samples) by Affymetrix SNP 6.0 mapping array analysis by studying 48 ccRCC tumour genomes in total. Our workflow (Fig 1) provides a strategy how to stratify genome-wide copy number alterations (CNA). Noteworthy, CNA data sets of ccRCC tumours available from TCGA encompass only 10 G1 tumours with limited access to clinical information [30]. Regarding the HRO study, gene members were categorized in a genome-wide unbiased gene-centred CNA approach comprising copy number alterations in at least 20 out of 48 ccRCC tumour samples. Firstly, genomic losses and gains of 15762 CNA genes affected were broken down to the gene level and related to Fuhrman malignancy grades G1 and G3. Secondly, CNA gene alterations mapped and grouped according to their Fuhrman assignments were assigned to chromosomal cytoband loci. Thirdly, the most prominent CNA gene losses and gains present in 48 individual ccRCC tumours were related to losses and gains of suppressor and driver genes to their occurrence in at least 20 HRO tumours and to their contribution in 280 KEGG pathways. Finally, CNA gene sets HRO286 and HRO201 have been established and validated indicating that CNA signatures are suitable to stratify ccRCC tumour subtypes as confirmed by CNA data available under GSE19949. The workflow of the copy number analysis performed entitles three different workflow approaches (Fig 1): A. Gene/genome-directed approach (Workflow Branch WB I), B. Patient-centered approach (Workflow Branch WB II) and C. Pathway-directed approach (Workflow Branch WB III).

## Results

The SNP 6.0 Affymetrix microarray analysis of 48 ccRCC tumour genomes resulted in 15762 separate gene alterations that at least once occurred in one HRO tumour. In total, 30720 gene losses and 32872 gene gains were detected in the HRO cohort (Table A and B in S1 File). Most of the CNA contributed either exclusively to 6174 gene losses (min: 15, max: 2918), respectively to 6532 gene gains (min: 38; max: 3248). Apparently, 3056 individual CNA gene members (min: 20; max: 1369) showed both, losses and gains, in the HRO cohort designated mixed-type CNA genes (Table A in S1 File). Mixed-typed genes that appear either in haploid or triploid copies are preferentially expected to induce significant changes on the transcriptional level as long as these genes epigenetically are not transcriptionally repressed. Thus, CNAs of mixed-type genes are considered to be putative nominators of predictive or prognostic power. The Swiss cohort GSE19949 (Table C in S1 File) represents 6 Fuhrman grade G1, 10 grade G2, 12 grade G3 and 2 grade G4 classified by 22601 CNA gene members (min 266, max 8291), of which 10872 CNA genes are exclusively losses (min 11, max 6041), 7096 genes gains (min 0, max 3375) and 4633 mixed-type CNA genes (min 83, max 2795), see Table A in S1 File. Regarding the HRO CNA data, ratios of gene losses to gene gains of 26 individual Fuhrman G1 genomes versus 20 individual Fuhrman G3 genomes shift from a ratio of 1.25 to a ratio of 0.58 indicating that gains of genes are an important feature for enhancing malignant processes in tumours (Table A in S1 File). Discriminatory power of CNA genes in regard to Fuhrman grades G1 and G3 was stratified by p-values based on Fisher’s exact test (S2 File).

### Genome directed CNA analysis (WB I)

The Workflow Branch WB I is focussed on selecting copy number alterations by molecular terms that are suitable to distinguish ccRCC tumours initially graded by pathologists using the Fuhrman classification [38]. As such, genome directed CNA analysis was initiated by assigning CNAs to gene loci, to mixed-type CNA genes and to oncogenes in respect to Fuhrman grades.

#### Gene loci directed CNA analysis

Gene losses were determined in 3p (Table K in S2 File): CNA assignments of 559 genes lost at chromosomal loci on 3p were interrogated to stratify tumours based on losses present at individual cytoband loci (Table P in S2 File). CNA genes present at cytobands on chromosome 3p such as 3p21.1 (37/37 genes), 3p22.1 (36/36 genes), 3p25.3 (47/49 genes) and 3p21.31 (140/149 genes) did hardly show any association with Fuhrman grades. CNAs of 257 genes such as *LIMD1-AS1* and *SACM1L* at 3p21.31 classified 10 and more G1 tumours in respect to G3 tumours, see Table P in S2 File. S*H3BP5*, *CAPN7*, *SH3BP5-AS1*, *ATRIP*, *LIMD1*, *SNRK* and *COL6A4P1* showed even greater differences (21 G1 vs. 4 G3; 20 G1 vs. 4 G3 and 21 G1 vs. 5 G3 HRO tumours) with p-values ranging from 6.7·10^−05^ to 0.00025 (Fisher’s exact test). CNAs of genes that were altered in G1 but only once in G3 are *METTL6* (p<0.00012), *TRIM71* (p<0.00017), *SLC25A38*, *IRAK2*, *VHL* and *CMTM8* with p<0.00044, *SNORA6* (p<0.00109) and *DCP1A*, *TKT*, and *ZNF852* with p<0.00257, and *ZNF852* with p<0.00588, see Table P in S2 File. In conclusion, cytoband loci at 3p represent signatures of gene losses contributing to Fuhrman grade G1, the less malignant phenotype. Note, losses in the *VHL* locus 3p25.3 plus gains in loci at 5q are shared at 5q31.2 and 5q31.1 by at most 21/48 tumours (14/26 G1 vs. 7/20 G3, respectively 13/26 G1 vs. 9/20 G3). Apparently, locus 5q31 did not show any preference for Fuhrman malignancy grades (Table A in S3 File). Furthermore, losses of *VHL* seemed to be associated more frequently with Fuhrman malignancy grade G1 independently of losses and gains of CNA genes at loci on 5q (Table B in S3 File). Recently, Moore et al. [39] reported in line with Chen et al. [40]) that *VHL* wild-type ccRCC tumours were observed to have higher number of genetic instabilities suggesting a greater potential for tumour progression, as copy number alterations have been associated with tumour stage, grade and worse prognosis. Median progression-free survival and ccRCC-specific survival were significantly reduced in patients with wild-type *VHL* expression [41].

#### Analysis of mixed-type CNA genes

**Fig 2:**
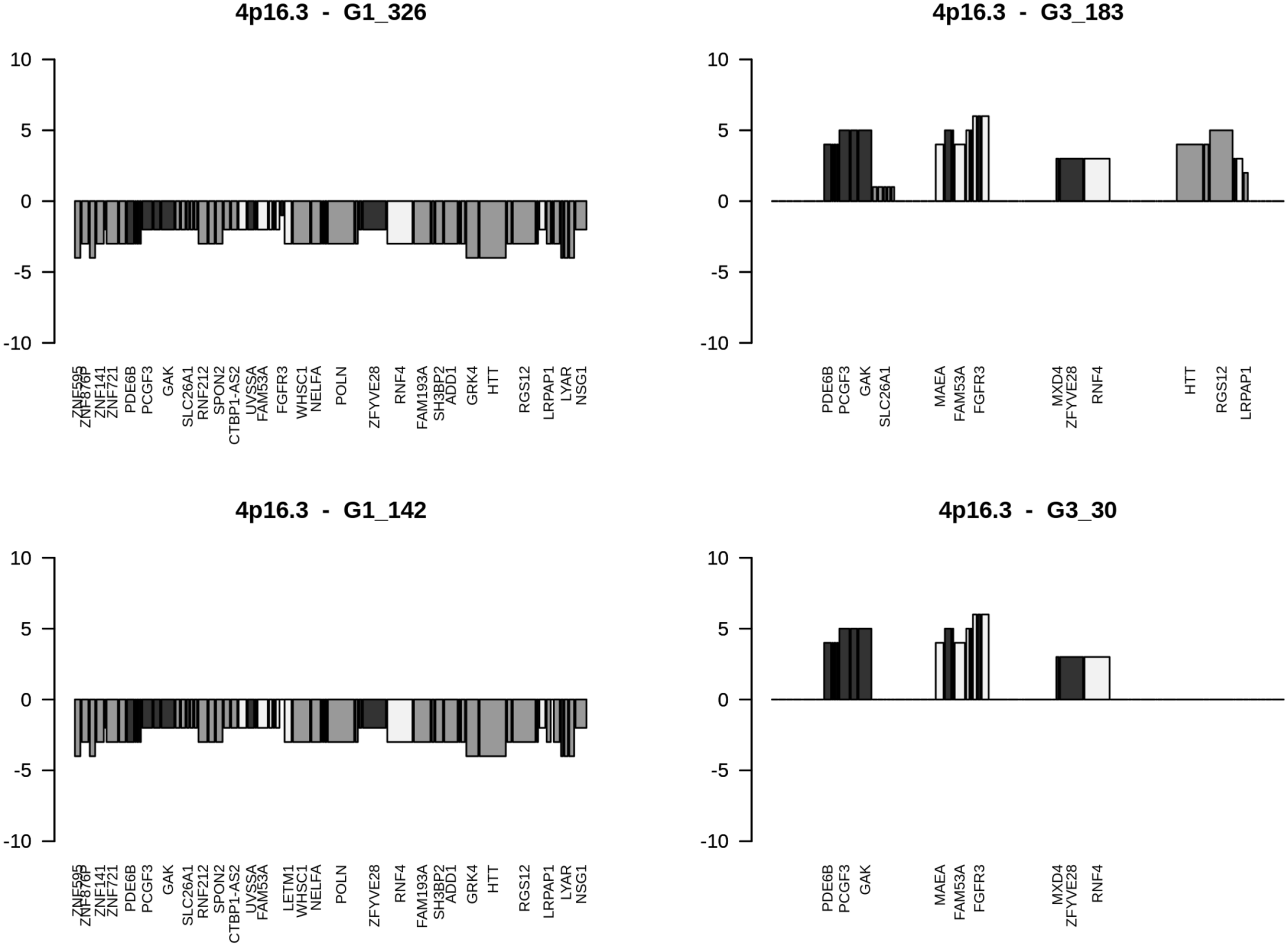
Gains and losses of CNA genes ordered at 4p16.3. Cytoband-plots of 4p16.3 show CNAs of selected HRO tumours. Corresponding data are available at Table F in S8 File. The height of bars documents number of tumours that share CNAs. Assignments of colourings are dark = G3 nominator genes, medium gray = G1 nominator genes, light gray = genes without any preference for Fuhrman malignancy grades.

**Fig 3:**
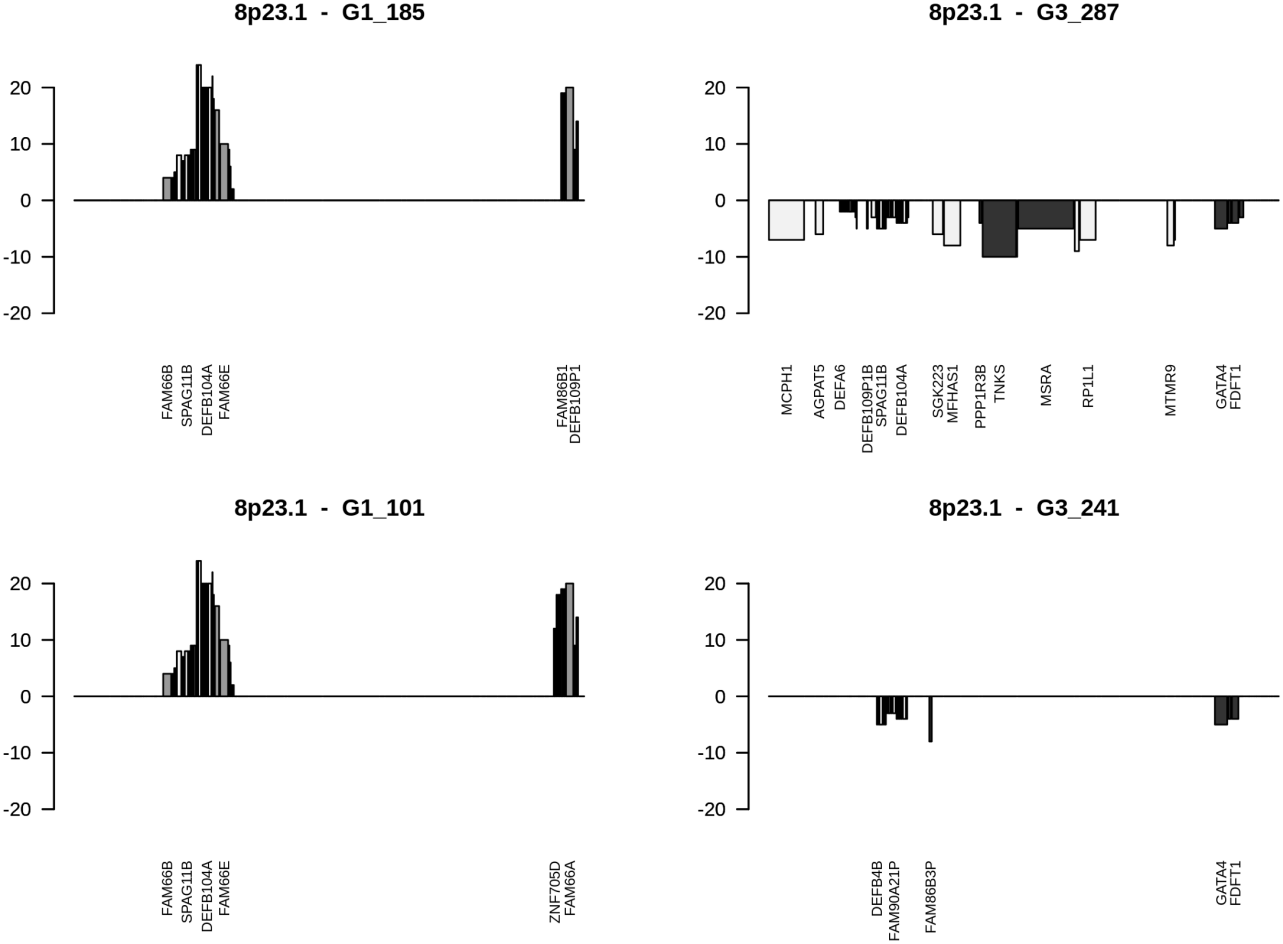
Gains and losses of CNA genes ordered at 8p23.1. Cytoband-plots of 8p23.1 show CNAs of selected HRO tumours, corresponding data at Table G in S8 File. Assignments of colourings are dark = G3 nominator genes, medium gray = G1 nominator genes, light gray = genes without any preference for Fuhrman malignancy grades.

**Fig 4:**
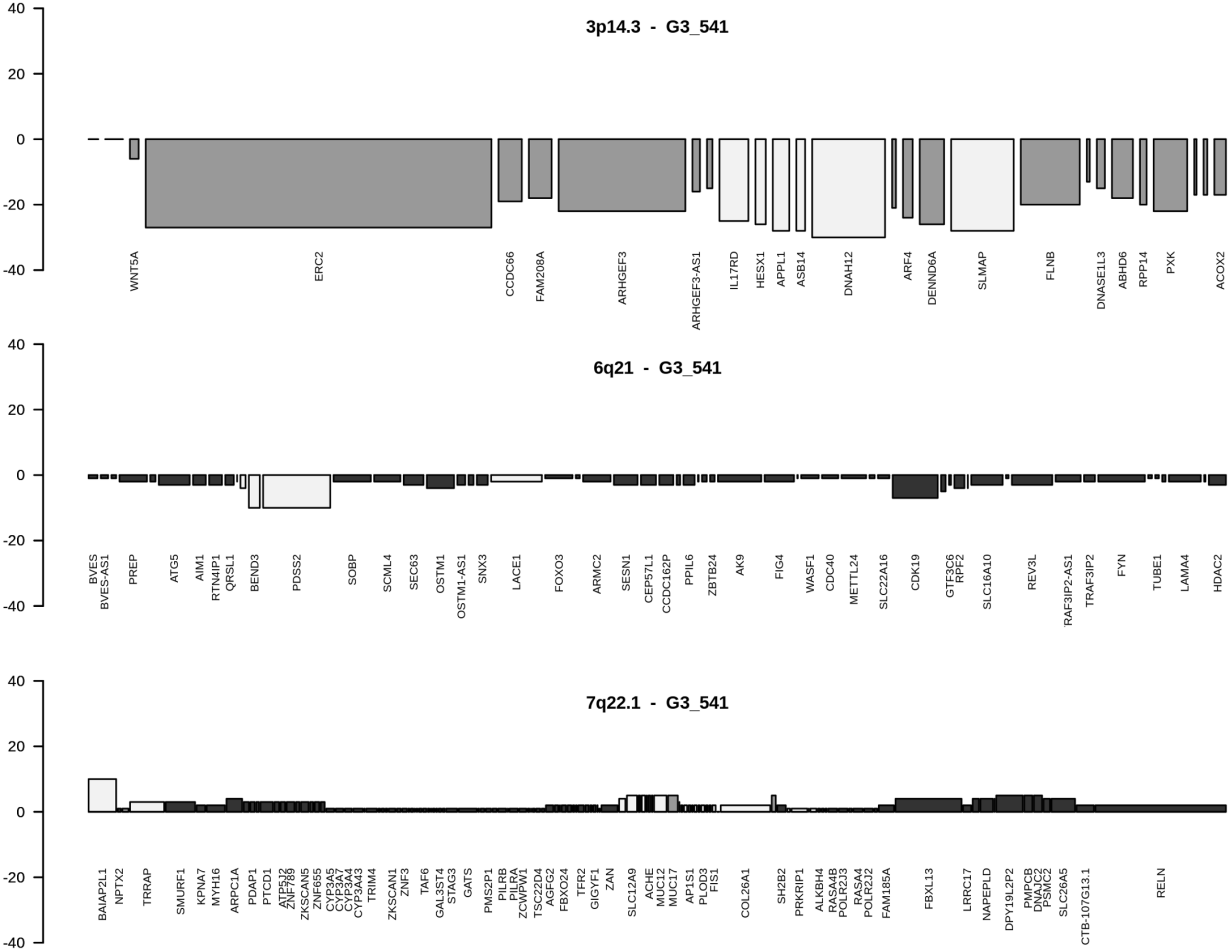
CNA genes in HRO tumour G3_541 at cytobands 3p14.3, 6q21 and 7q22.1. Cytoband-plots of HRO tumour G3_541 depict three cytobands affecting almost all genes (bars are present), data at Tables H-J in S8 File. Assignments of colourings are dark = G3 nominator genes, medium gray = G1 nominator genes, light gray = genes without any preference for Fuhrman malignancy grades.

Mixed type genes (Table B in S2 File) are considered to be of high relevance since a loss in G1 and a gain in G3 or vice versa are expected to lead at specific chromosomal loci to quite opposite signatures resulting in enhanced phenotypes as exemplified by gene losses opposed to gene gains and vice versa. From initially 3056 mixed-type genes (Table A in S1 File), 195 genes were present in at least 6 tumour genomes with gains in 2 to 23 and losses in 2 to 21 tumour genomes (Table B in S2 File). *PUS7* at 7q22.3 was amplified in 3 G3 tumours and lost in 7 G1 tumours (Fisher’s exact p-value: 0.0050343). CNA genes predominantly amplified were genes at loci 8p23.1, Xq28, 15q11.2, and 17q21.31. Losses predominantly mapped to 5q11.1 (*EMB*), 3p21.1 (*SNORA26*) and 3p25.3 (*SNORA43*). Fuhrman G1 and G3 gene assignments revealed that CNA genes at loci 9q34.3, 8q, 7q36.1 and 9/16 genes at 4p16.3 go along with Fuhrman grade G3, whereas CNA genes at chromosomal loci 14q11.2 and 15q11.2 favour a G1 phenotype underlined by p-values of Fisher’s exact test (Table B in S2 File). At locus 4p16.3, gene losses favour Fuhrman grade G1, while gene gains support grade G3 phenotypes (Fig 2). At locus 8p23.1, gene gains direct to Fuhrman grade G1, gene losses to grade G3 phenotypes (Fig 3). In cases CNA genes of grade G3 ccRCC tumours map to loci assigned as Fuhrman grade G1 as exemplified by locus 3p14.3, losses and gains at additional loci are most likely nominators for specifying final Fuhrman grades such as losses at 6q21 and gains at 7q22.1 in respect to losses common to chromosomal loci at 3p. CNAs at distinct chromosomal loci are shown to lead to G3 phenotypes as visualized by ccRCC G3-541 (Fig 4). Comparative analyses of losses and gains of individual loci indicate the presence of ordered processes of copy number alterations that occur in numerous HRO tumour genomes suspected to be guided by common mechanisms. (Figure C in S4 File)

#### Chromosomal loci directed CNA analysis

**Fig 5:**
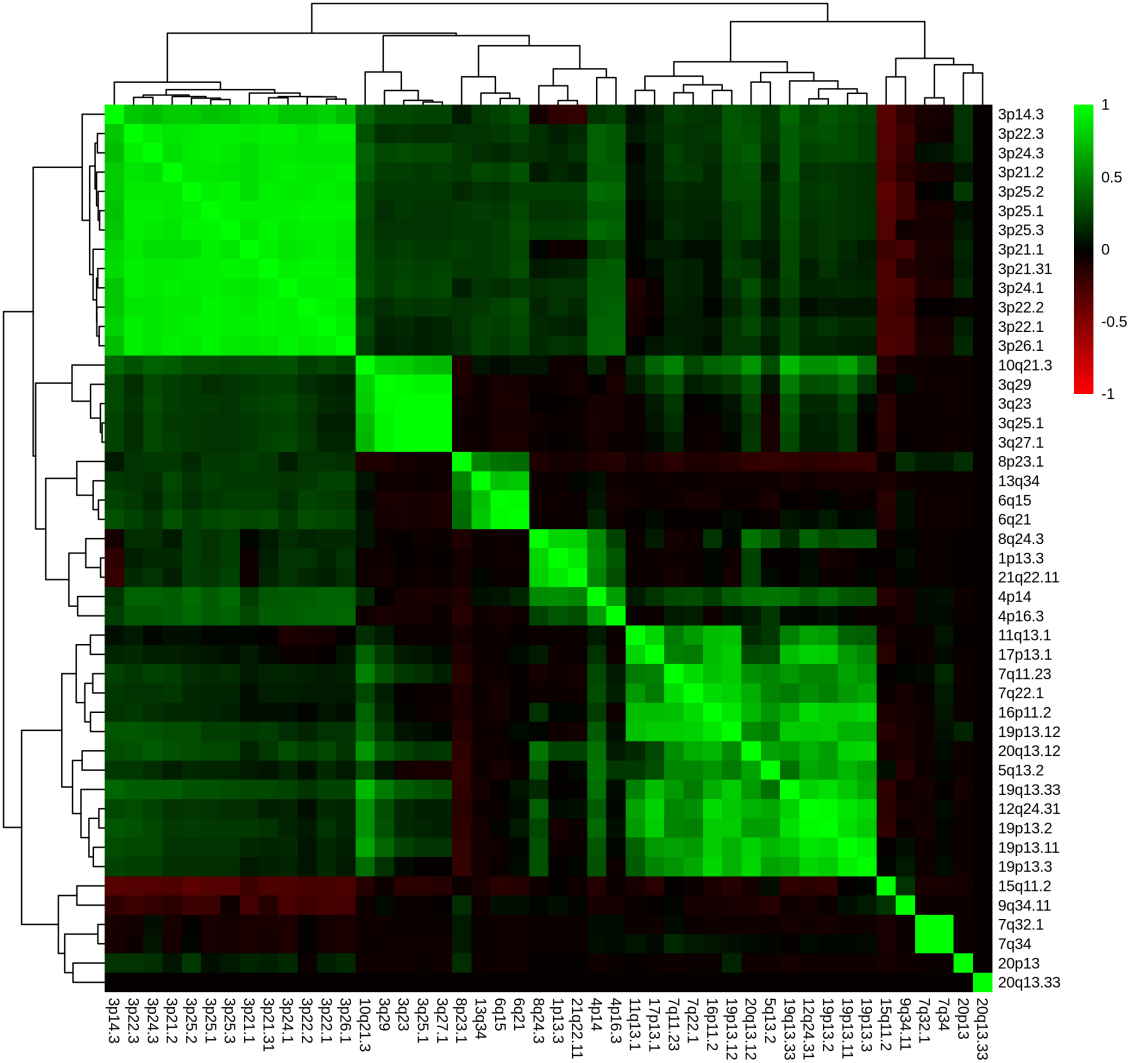
Correlation of cytobands based on gene losses. Unsupervised hierarchical clustering by average linkage and Euclidian distance, representing a correlation-matrix displaying Pearson correlation of gene losses between cytobands specified by p-values below 10^−14^ (Table C in S5 File).

**Fig 6:**
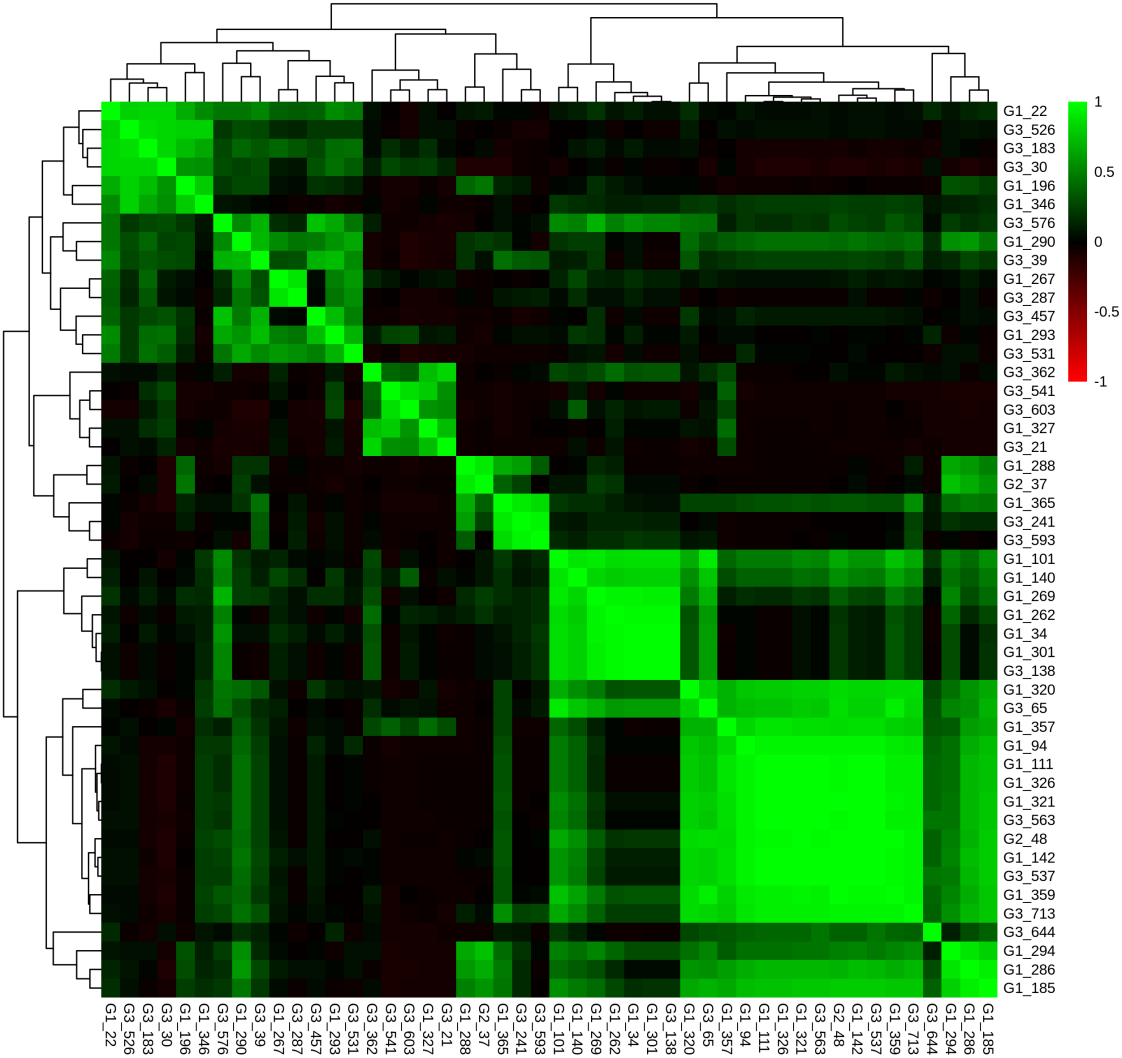
Correlation of ccRCC tumours based on gene gains in cytobands. Unsupervised hierarchical clustering by average linkage and Euclidian distance, representing a correlation-matrix displaying Pearson correlation between HRO tumours based on CNA gains per cytoband specified by p-values below 10^−14^ (Table C in S5 File).

CNA genes were assigned to chromosomal loci of 48 ccRCC tumours (Table A in S2 File) according to their localisation at 795 chromosomal cytoband loci (Table A in S5 File). Each chromosomal locus was characterized by the number of CNA gene losses and gains including the number of HRO tumours and the distribution of Fuhrman grades G1 and G3. P-values of Fisher’s exact test ranked chromosomal loci to stratify ccRCC tumour genomes by analysing Fuhrman gradings in respect to CNA gene losses and gene gains (Table A in S5 File). At least 20 HRO tumours are stratified by 82 cytobands. Thus, 25 chromosomal bands display p-values below 10^−14^ (Table B in S5 File). The other 57 chromosomal loci such as 5q33.3, 5q34 and 5q35.3 do not show any discriminatory power regarding Fuhrman grading. In total, the HRO tumours have been characterized by 50 cytobands with p-values below 10^−14^ (Table C in S5 File) leading to CNA gene losses and CNA gene gains. In essence, assignments of HRO tumours to CNA gene losses at chromosomal loci do hardly distinguish ccRCC subtypes (Fig. A, C and E in S6 File). Individual chromosomal loci were compared with each other (S6 File) using Pearson correlation analysis either by including all cytobands representing CNA gene losses (Fig. A in S6 File), gene gains (Fig. B in S6 File) or by selecting cytobands of CNA losses (Fig. C in S6 File) and gene gains (Fig. D in S6 File) present in at least 20 and more ccRCC genomes. P-values of Fisher’s exact test underline the contribution of Fuhrman gradings G1 and G3 on CNAs assigned to individual cytobands (S5 File). Chromosomal loci stratified by p-values of Fisher’s exact test describe CNA gene losses and gains of chromosomal bands in respect to Fuhrman gradings. Correlations of chromosomal loci stratified by p-values of lower than 10^−14^ determine CNA gene losses (Fig 5) and gene gains (Fig. E in S7 File) of the most informative chromosomal bands. In particular, gene losses and gains were assigned to individual chromosomal loci by determining chromosomal loci covering CNA gene losses (Fig. A in S7 File) or gene gains (Fig. B in S7 File) as well as chromosomal loci based on gene losses and gains in at least 20 or more ccRCC tumours (Fig. C and D in S7 File). Inspections of individual CNA loci underscore the presence of ongoing chromosomal alterations. CNA data sets of this kind indicate to be suitable for stratifying ccRCC in early and more advanced ccRCC tumour subtypes. Gene losses seem by majority to be a common feature in ccRCC tumorigenesis. Correlation analyses of cytoband loci based on gene gains demonstrate the putative presence of ccRCC subtype-specific features (Fig 6 and Fig. D in S6 File) confirming the discriminatory power of CNA gene gains in comparing ccRCC tumours and in stating the presence of highly and less highly related CNA signatures capable of distinguishing ccRCC tumours within the HRO cohort. Apparently, copy number alterations seem to obey and follow specific constraints in a non-random fashion that reoccur in a similar and comparable manner in numerous ccRCC tumours (S8 File).

### Oncogene directed CNA analysis

In line with current ccRCC tumour models [42] gains of oncogenes and losses of tumour suppressor genes are considered to be powerful events contributing to tumourigenesis. Thus, 419 tumour suppressor genes [43] downloaded from http://bioinfo.mc.vanderbilt.edu/TSGene/ and 254 driver genes [44] downloaded from http://www.intogen.org/web/mutations/v04 were analysed including 49 genes being assigned to harbour either tumour driver or suppressor activities.

#### CNAs of tumour suppressor genes

Regarding CNAs present in 419 tumour suppressor genes (Table C in S2 File), losses of the most common CNA tumour suppressor genes are found at 3p21.31 specified by genes *RBM6* in 35 HRO-, *RBM5* in 30 HRO-, *LIMD1* in 28 HRO-, *TCTA* in 26 HRO- and at 3p22.2 by *MLH1* in 33 HRO-tumours. CNA gains of suppressor genes are enlisted by gains of *HADAC3* in 18 HRO-, *COL18A1* in 16 HRO-, *TGFBI* in 16 HRO- and *CDH4* in 15 HRO tumours (Table C in S4 File). Losses of *RBM6* (23/26 G1; 10/20 G3), *MLH1* (22/26 G1; 9/20 G3) and *LIMD1* (21/26G1; 5/20 G3) on 3p are predominantly present in Fuhrman G1 HRO tumours (Table C in S4 File). Gains of 4 tumour suppressor genes such as *PAX4* at 7q32.1, *NAPEPLD*, *ARMC10*, and *FBXL13* at 7q22.1 were determined in 4/20 G3 tumours as well as 5 single gene gains at loci at 1q21.2 (*PLEKHO1*), 11q13.1 (*PLCB3*), 12p13.1 (*EMP1*), 18p11.21 (*PTPN2*), and 19p13.3 (*PLK5*) additionally present in 5/20 G3 tumours exclusively specifying Fuhrman G3 tumours (S1 Table). Interestingly, similar CNA signatures are present in the Swiss cohort (S1 Table). Note, in cases epigenetic processes as histone methylation lead to transcriptional silencing, CNA gene losses and gains of these genomic segments are expected to be of less functional relevance. As such, losses of tumour suppressor genes that are initially not expressed do necessarily not indicate growth advantages leading to more malignant tumours or vice versa. Keep in mind, CNA suppressor genes linked with more than one pathway adds another level of complexity. CNAs of suppressor genes present in at least 5 renal tumour genomes linked *PPP3CC*, *PPP2CB* and *RBX1* in 9/11/7 HRO tumours with 19/11/11 KEGG pathways (Table C in S2 File). Losses and gains of individual tumour suppressor genes cannot exclusively be related to one individual cellular function.

#### CNAs of driver genes

Chromosomal losses and gains regarding 254 driver genes analysed determined losses of *SETD2* (43/48) and *MAP4* (38/48) at 3p21.31 assigned to Fuhrman grade G1 in 24/26 G1 and 23/26 G1 tumours, respectively, to 17/20 G3 and 13/20 G3 tumours. Four top-ranked driver genes that favour a G1 assignment were found to be exclusively lost on loci at 3p22.1 by *CTNNB1*, at 3q22.3 by *PIK3CB*, at 10q21.3 by *CCAR1*, and at 17q12 by *CDK12*, supported by gains at 1q44 by *NLRP3*, at 2q23.3 by *RIF1* and at 2q31.2 by *NFE2L2*. Assignments of Fuhrman grade 3 identified 6 top-ranked driver loci, of which 4 described losses exclusively at loci 14q13.2 by *RALGAPA1*, 14q23.3 by *MAX* and 14q32.12 by *TRIP11* 4 and *DICER1* including 2 gains at 5p15.32 by *KIAA0947* and at 2q21.1 by *POTEF* (Table D in S2 File). CNA driver genes such as *PIK3CB* lost in 9 tumours, *PIK3R1* and *AKT* amplified in 6 and 4 tumours respectively are linked with 58, 57 and 53 KEGG pathways underlining the tremendous effects of individual gene losses and gains.

#### CNA genes assigned as driver or suppressor genes

Numerous genes have been published to present either drivers or tumour suppressor genes (Table E in S2 File). Regarding these 49 CNA genes, Fuhrman grade G1 identified 7 gene losses of Fuhrman grade G1 on 3p e.g. specified by *RHOA* in 20/26 at 3p21.31 and by *BAP1* at 3p21.1 in 19/26 G1 in respect to 4/20 G3 cases. Respectively 3/20 G3 HRO tumours were specified by *ARHGAP35* at 19q13.32 and by *FOXA2* at 20p11.21. Interestingly, *BAP1* mutations in 15% of all ccRCC [45] have been reported together with *SETD2* mutations being more frequent in higher stage tumours and associated with worse prognosis [46]. Accordingly, copy number alterations have to be put into perspective to occurring mutations and measured protein expression levels in order to rank the oncogenic potential of individual CNA genes.

### Patient-directed tumour analysis (WB II)

Patient-directed assignments were focussed on determining CNAs present in 20 and more ccRCC tumours (TP20 data sets). CNA signatures designated TP20 were primarily derived from Fuhrman grade G1 and G3 tumours of 46/48 ccRCC tumour genomes. Two Fuhrman ccRCC tumours of grade G2 were excluded from comparative CNA analyses between grade G1 and grade G3 tumours (S9 File).

#### TP20—Gene losses and gene gains

Gene list TP20 determines gene losses and gains of 370 genes occurring in CNAs of 20 and more HRO tumours present in up to 48 ccRCC tumour genomes (Table B and C in S9 File). Worth noting, the *VHL* gene at chromosomal locus 3p25.3 gene displayed only 16 gene losses found in 48 HRO tumours. The TP20 gene list predominantly encompasses 290 member genes encoded on chromosome 3p, of which 93 have been assigned to chromosomal locus 3p21.31 and 46 to chromosomal locus 3p22.1. The gene locus 3p21.31 represented by gene losses of *SETD2* (43 tumours), *KIF9-AS1* (43 tumours) and *KIF9* (42 tumours) seems to constitute one locus presenting common Fuhrman grade independent gene losses. Of *290* genes on 3p, only 7 genes showed gains, of which *SNORA26* (8 tumours) and *SNORA43* (4 tumours) were amplified in more than one tumour genome. Comparing gene assignments to CNA of gene losses in Fuhrman grades G1 and G3, a significant greater number of genes in 3p were predominantly lost in grade G1 vs. G3 tumour genomes: 5100 CNA events in 26 G1 genomes (mean values = 196 gene alterations per tumour genome) to 1803 CNA events in 20 G3 genomes (mean values = 90 gene alterations per tumour genome) (Table A in S9 File).

#### TP20 gene losses

The complete HRO data set was interrogated for TP20 gene losses (Table B in S9 File). 295 genes were scored to be present in at least 20 HRO tumours. In addition to genes at 3p listed in Table A in S9 File, gene losses showed up at 14q such as *ALDH6A1*, *ENTPD5*, *CCDC176* as well as *LIN52* at 14q24.3 in respect to *EMB* at 5q11.1 and *ADAM5* and *ADAM3A* at 8p11.22. The locus of *EMB* was scored as gene gain as well as gene loss, a mixed-type CNA gene. Comparing the tumour grades G1 and G3 in respect to gene losses, *LIMD1-AS1* and *SACM1L* at 3p21.31 together with additional genes at 3p21.31, 3p21.1, 3p22.1, 3p22.3, 3p25.1 and 3p25.3 underscore that losses of these genes/chromosomal segments favour the establishment of grade G1 ccRCC tumours. Interestingly, weighted analysis of gene losses assigned CNA genes at chromosomal loci 14q24.3 to Fuhrman grade G3 (p-value = 0.77): Most common CNA genes that segregate Fuhrman grade G1 from G3 were gene losses of chromosomal regions on 3p documented by p-values of lower than 0.01 representing 130 CNA genes and 0.001 representing 39 CNA genes, respectively (Table B in S9 File).

#### TP20 gene gains

Fifty-seven gene gains present at least in 20/48 tumour genomes are found at chromosomal loci 14q32.33 (6 genes), at 15q11.2 (4 genes), at 1p36.33 (1 gene), at 1q21.1 (8 genes), at 22q11.22 (4 genes), at 5p15.33 (4 genes), at 5q31.3 (19 genes), at 8p23.1 (9 genes) and at 9q21.11 (1 gene), see Table C in S9 File. Genes at 8p23.1 and at 15q11.2 belong to mixed-type CNA loci: They were predominantly amplified, but in fewer cases they were lost instead. The top-ranked six genes present at chromosomal locus 14q32.33 were detected to be amplified in 39 to 48 tumour genomes. The most abundant events of gene gains in line with Fuhrman grade G3 were genes such as *SRGAP2B* at locus 1q21.1 (Fisher’s exact test: p<0.0588) including 5 additional genes with p<0.1283 plus IGHV3-11 at locus 14q32.33 (Fisher’s exact test p<0.0326), see Table C in S9 File.

#### TP20 mixed-type gene segments

Twenty-six genes (Table D in S9 File) were selected that harbour losses or gains in at least 20 tumour genomes regarding gains in 4 to 23 and losses in 3 to 21 HRO tumour genomes. The TP20 gene list encompasses 26 genes with chromosomal loci at 15q11.2 (6 genes), at 17q21.31 (5 genes), and at 8p23.1 (9 genes). Single mixed-type genes at chromosomal loci were identified at 3p21.1, 3p25.3, 4p14, 4q13.2, 5q11.1 and Xq28 (Table D in S9 File). CNAs of gene *SNORA26* (3p21.1) and *UGT2B17* (4q13.2) were predominantly determined in G1 (Fisher’s exact test p<0.00811) and in G3 tumours (Fisher’s exact test p<0.137), respectively (Table D in S9 File).

### Pathway directed CNA analysis (WB III)

In total, 15762 CNAs resulted in 15402 gene assignments addressed in 280 Pathways (PWs). Six top-ranked pathways representing most abundant gene gains and losses were *PI3K*-Akt Signaling Pathway (264/364 genes), Pathway in Cancer (251/327 genes), Neuroactive-Ligand-Receptor-Interactions (208/275 genes), MicroRNAs in Cancer (202/296 genes), *MAPK* Signaling Pathway (191/259 genes) and Cytokine-Cytokine Receptor Interactions (185/265 genes), see Table D in S1 File. Pathway assignments identified 30/48 losses of *RHOA* linked with 25/280 KEGG pathways, followed by 14 CNA losses of *TGFBR2* in 14 pathways plus 12 CNA losses of *PRKCD* in 9 pathways. KEGG pathways addressing at least 20 and more HRO tumours were visualized (Fig. A in S10 File).

Pathways were selected whose CNA genes are at least present within 2 HRO tumours (Table B in S11 File). In total, CNA genes within 11 most common KEGG pathways have been identified to be present in at least 20 HRO tumours (Fig. B in S10 File).

Gene losses and gains of individual genes such as *RAF1* or *RHOA* interfered with pathway functionalities present in at least 8 different pathways (Table B in S11 File). CNAs of genes contributing to the most common top-ranked pathways (5/6) were predominantly gains of *EGFR*, *PDGFRA*, *PDGFRB*, and *PDGFA* (Table F in S2 File). Of all CNA gene gains and losses, the PI3K-Akt Pathway enlisted 264 genes of which *RAF1* (16 times lost), *ITGA9* (15 times lost) and *LAMB2* (15 times lost) were abundantly affected in HRO G1 ccRCC tumour samples. Regarding Fuhrman G3 grades *RPTOR* (10 times amplified), *PDGFRB* (8 times amplified) and *PRLR* (8 times amplified) have to be mentioned (Table G in S2 File). The *MAPK* Signalling Pathway (Table H in S2 File) highlighted 191 genes of which *RAF1* (21 losses), *CACNA2D3* (25 losses), *CACNA1D* (24 losses) and *FLNB* (20 losses) constituted G1 CNA genes and *BRAF*, *PPM1A* and *MAX* G3 CNA genes, respectively. Renal Cell Carcinoma Pathway (Table I in S2 File) assigned of 49/66 CNA genes *RAF1* (16/26) and *VHL* (14/26) as G1 top-ranks with Fisher’s exact test p-values of 0.00228 and 0.0004437 (respectively) and *SOS2* (7/20), *HGF* (4/20) and *BRAF* (3/20) as G3 top-ranks with p-values of 0.17, 0.51 and 0.075, respectively. Pathways in Cancer (Table J in S2 File) describe of 251/327 genes *RAF1* and *VHL* again as G1 nominators followed by *WNT7A* (15/26) and *MLH1* (22/26) with p<0.00553, respectively p<0.00985. *MAX* (7/20) and *FZD9* (3/20) specified G3 assignments with p<0.0288 and p<0.075, respectively. Worth to note, CNAs of 37 genes were exclusively found in G3 tumours such as *WNT7B* (4 gains), *BAD* (3 gains), *WEGFB* (3 gains) and *HDAC2* (3 losses) in respect to 65 CNA genes exclusively presented in G1 tumours, such as *GRB2* (5 losses) and *FGF21*, *STAT3*, *CTNNA3* and *DVL3* with 3 losses each. Regarding pathway assignments of at least 20 HRO tumours (Table A in S11 File), 10 top-ranked genes, of which 9 were lost in 3p, were linked with 7 to 45 pathways (n = 280) comprising *RAF1* (45 PWs), *RHOA* (25 PWs), *IPTR1* (22 PWs), *CACNA1D* (21), *ITGA9* (8PWs), *WNT7A* (8 PWs) *TLR9* (7PWS9, *LAMB2* (7 PWs) and *ARPC4* (6 PWs) (Table B in S11 File). A comprehensive illustration of all connectivities representing the most common pathways addresses the participation of particular CNA genes in cellular gene regulation (Fig. B in S10 File / Table B in S11 File). As such, distinct gene losses and gains occurring in individual tumour genomes prioritize pathway assignments suitable to predict and evaluate functionalities of malignancy grades of individual tumours with the potential of offering more individualized treatment options.

#### Pathway PI3K related CNAs assigned to HRO tumours

**Fig 7:**
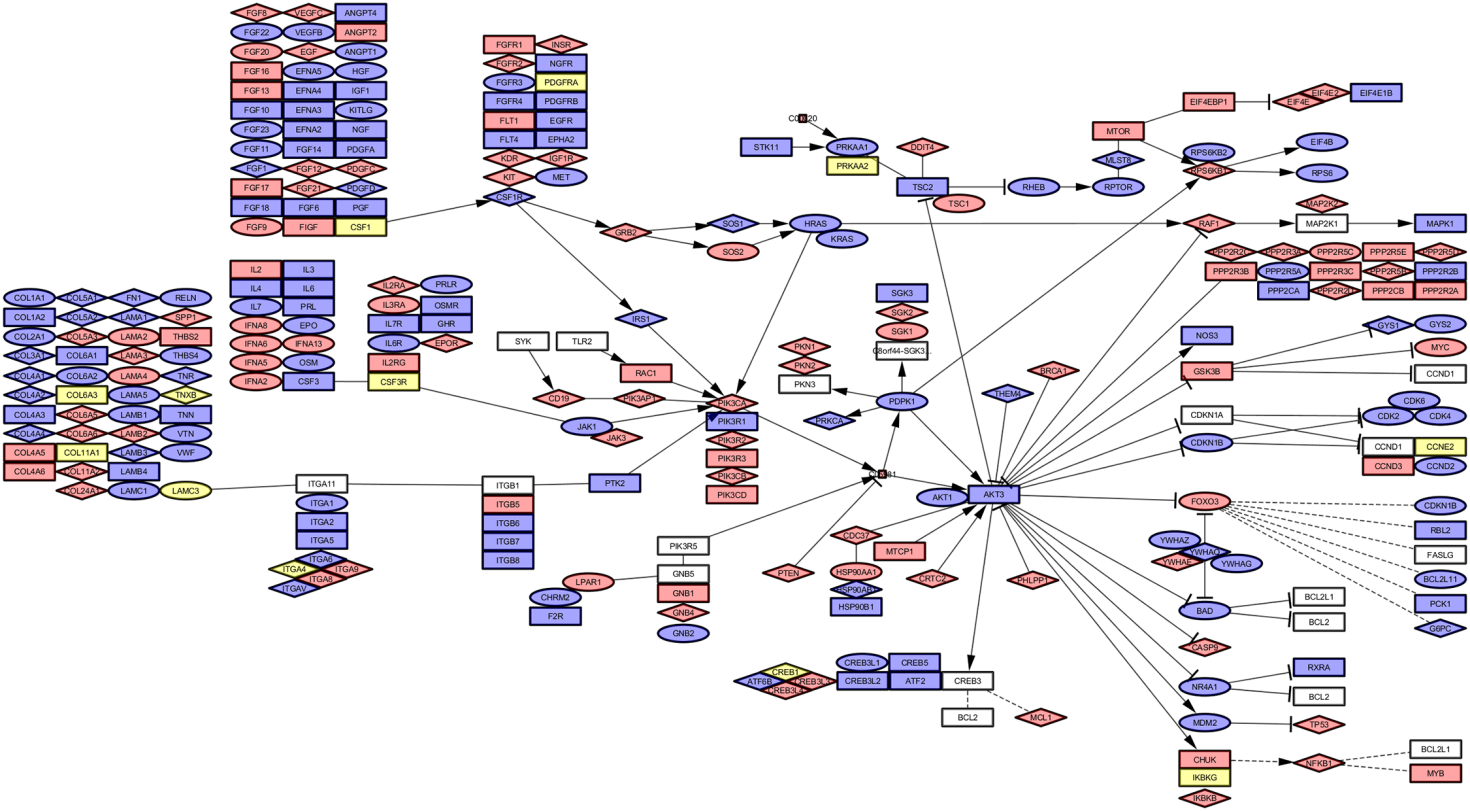
CNAs of all HRO tumours assigned to PI3K pathway. Decomposition of the KEGG Pathway PI3K shows a colour coded map of genes. The shapes are: Red = LossBlue = GainYellow = Both states (Gain/Loss)Rectangle = Uncoloured, Gene is not affectedRectangle = Grade (1/3) equally affected, colour shows aberrationCircle = Grade 3, colour shows aberrationDiamond = Grade 1, colour shows aberration Corresponding data sets are enlisted at Table G in S2 File.

The *PI3K*-*AKT*-*mTOR* pathway is considered to be a relevant therapeutic target as recently discussed by Slomovitz et al. [47] in endometrial cancer and in RCC by the Cancer Genome Atlas Research Network [30]. In particular, the *PI3K*-*AKT*-*mTOR* pathway is considered to have an important role in cellular growth and survival [48] due to intersections with other signalling pathways, for example, the *RAS*/*RAF*/*MEK* pathway [49]. Our comparative analyses of CNA assignments to KEGG pathways identified the PI3K pathway (HSA04151) with 264/346 CNA genes as the pathway most commonly hit by CNA losses and gains. The *PI3K* pathway touched dominant CNA gene gains of *PPP2R2B*, *RPTOR* and *PDGFRB* and gene losses of *ITGA9*, *LAMB2* and *RAF1* (Table G in S2 File). Gene losses representing Fuhrman grade G1 were represented by *RAF1*, *ITGA9* and *LAMB2*. Gains of grade G3 seemed to be associated with *RPTOR*, *PDGFRB* and *PRLR* (Table G in S2 File). CNAs of all 48 HRO tumour genomes were mapped to the *PI3K* pathway (Fig 7) in comparison to corresponding CNAs derived from individual ccRCC genomes specified by ccRCC G1_365 (S13 Fig 1) and by ccRCC G3_287 (Fig. B in S12 File). To visualize the impact of CNA genes within the *PI3K* pathway, CNA genes predominantly contributing to Fuhrman grade G1 were illustrated by diamond shapes, and CNA genes predominantly contributing to Fuhrman grade G3 by circles, respectively. Blue-coloured shapes represent gene gains and red-coloured gene losses (Fig 7, S12 File). Noteworthy, in line with visualizations of diamond shapes and circles assigned to the *PI3K* pathway, *PI3K* signaling has previously been reported to be involved in complex processes with seemingly opposing functional effects such as cancer progression and anti-tumour response on one hand and escape mechanisms from immunological surveillance and immune suppression on the other hand [50]. Due to combined functionalities of CNA gene losses or gains stratifying either G1 or G3 ccRCC phenotypes, ambiguities in predicting functionalities of *PI3K* signaling become apparent and explainable (Fig 7). As outlined, patient specific CNA signatures of *PI3K*-*AKT*-*mTOR* pathway related genes might add additional prognostic information in ccRCC patient management in line with recent reports on targeted therapies by Haddad and Margulis [42] and other colleagues [51].

### CNAs in respect to Fuhrman classification

Fuhrman grading was used to stratify each gene loss or gain in respect to its presence in 46 Fuhrman grade G1 (n = 26) or grade G3 tumours (n = 20) ranked by Fisher’s exact test (Table A in S2 File). CNA genes were assigned to chromosomal loci (S5 File) and stratified by their contribution to Fuhrman classification based on Fisher’s exact test (Table N and O in S2 File). In particular, 172 (849) CNA genes were determined to discriminate between Fuhrman grade G1 (n = 26) and G3 (n = 20) by Fisher’s exact test (p-values p<0.01 / Table A in S13 File; p<0.1 / Table B in S13 File). The most common 44 genes (p<0.001) including *VHL* enlist *MON1A*, *CCDC51* and *TMA7* at 3p21.31 (31/29 HRO tumours), and *MIRLET7G* and *WDR28* at 3p21.1 (31/31 tumours), respectively (Table C in S13 File). CNA genes categorized by p-values of p = 1.0 (Fisher’s exact test) did not distinguish between Fuhrman grade G1 and grade G3. Thus, these CNA genes most likely constitute common nominators of ccRCC tumourigenesis, see supporting information S15 text. For instance, most common gene losses at 3p14.3 of *APPL1*, *SLMAP* and *ASB14* present in 15/26 G1 and 11/20 G3 tumours (Fisher’s exact test p-values of p = 1) differ from gene losses of genes such as *CCDC66* and *ARHGEF3* present in 19 and 22 tumours assigned to Fuhrman grade G1 in 15 and 16 cases, respectively (Fisher’s exact test p-values of p<0.0055 and p<0.0071, Table A in S4 File). Regarding loci 16p13.3 and 19p13.3, a considerable number of genes was either assigned to Fuhrman grades G1 (33 genes, respectively 32 genes) or G3 (12 genes, respectively 47 genes) besides 114, respectively 88 CNA genes that do not show any bias towards Fuhrman grading (S5 File).

Cytoband 7q22.1 favours Fuhrman grade G3 (87/124 genes) in respect to cytoband 16p11.2 representing Fuhrman grade G1 (70/107 genes), see Fig. D and E in S4 File. Fuhrman grade G1 related CNA gene loci are predominantly found on 1q32.1 (43/81 genes), 4q13.3 (35/40 genes), 10q22.1 (31/47 genes), 16p11.2 (70/107 genes), 19p13.12 (30/30 genes), and 10q26.13 (30/38 genes). Fuhrman G3 nominator genes are predominantly located on 6q21 (39/57 genes), 9q34.11 (27/32 genes), 8q24.3 (23/102 genes) and 12q13.3 (11/48 genes), see S5 File. A closer inspection of CNA loci in Table C in S5 File indicates that Fuhrman grading G1 and G3 are distinguishable by chromosomal alterations. Fuhrman grade G1 related CNA genes were predominantly present at loci 19p13.12 by 30/30 and 16p11.2 by 70/107 CNA genes. Grade G3 related genes were determined at 12p13.31 by 39 of 60 CNA genes and at 7q11.23 by 32/77 CNA genes (Table B in S5 File). Comparative CNA analysis discriminating between G1 and G3 tumours outlined either 681 CNA genes exclusively found in at least 1/26 Fuhrman G1 tumour or 486 CNA genes present in at least 1/20 Fuhrman G3 tumour (Table C in S3 File). Corresponding chromosomal loci are enlisted in Table C in S5 File. CNA gene losses and gains of cytoband 14q32.33 (Fig. C and D in S4 File) visualize the contribution of Fuhrman gradings G1 and G3 and the physical order of ongoing copy number alterations.

### Gene set HRO201

Our initial CNA analysis performed on ccRCC tumour genomes was based on the conceptual understanding that copy number alterations start as lower malignant tumours and progress to higher malignant ccRCC tumours. The analysis of CNA loci as depicted in Figs 5 and 6 followed by CNA gene assignments that favour either Fuhrman grades G1 or G3 (Table C in S3 File) fuelled a combinatorial approach for determining the most informative CNA gene losses and gains. Finally, a CNA gene set of 201 CNA genes that differentially occur in Fuhrman G1 and G3 tumours has arithmetically been determined in respect to their presence and abundance in G1 and G3 ccRCC tumours, see Material and methods. This approach of determining CNA subsets has been restricted to gene sets being at least altered in 4/20 G3 or 5/26 G1 ccRCC tumour patients, see Material and methods. This gene set of HRO201 displays a subset of CNA genes initially enlisted with p-values of p<0.1 by Fisher’s exact test (Table 1). This current CNA subset constitutes of 93 CNA genes stratifying tumour grade G3 and 108 CNA genes tumour grade G1, respectively. Regarding the HRO201 gene set, 185 genes have been successfully determined in TCGA data regarding ccRCC tumours, and 171 genes in the Swiss data set, respectively (Table 1). The Swiss data set was preferentially taken for further comparative analysis of distinct CNA gene sets. Respectively, TCGA ccRCC data display significantly higher numbers of copy number alterations (Table A in S1 File). The number of CNAs per ccRCC tumour was significantly higher in the TCGA data (5743.8 CNA genes per patient) than in the Swiss data set (2994.8 CNA genes per patient) compared to the HRO cohort (1324.6 CNA genes per patient), see Table A in S1 File. Thus, gene set HRO201 (Table B in S14 File) was subjected to comparative analyses of HRO (Table B in S1 File) and Swiss cohort tumour data (Table C in S1 File). Correlation matrix analysis identified mutual ccRCC subtypes in both ccRCC cohorts (S1 Fig). The Swiss cohort encompasses Fuhrman grade G1, one grade G2, 6 grade G3 and one grade G4 tumours, whose CNA signatures did highly correlate with signatures of at least 5 HRO Fuhrman G3 tumours in contrast to 5 HRO Fuhrman G1 tumours (S1 Fig). CNA composition of these 20 ccRCC tumours enlisted in Table C in S14 File confirmed that distinct CNA losses and gains, initially determined by relating CNA signatures to Fuhrman grading of the HRO cohort, are present in the Swiss cohort as well.

**Table 1:** Comparative analysis of overlapping CNA gene sets.

Gene Set	Data-Source	HRO286	p<0.001	p<0.01	p<0.1	+G3	+G1	HRO201	Swiss All Genes	HRO All Genes
**HRO All Genes**	S2.A	286	44	172	849	93	108	201	12837	**15762**
**Swiss All Genes**	GSE19949	251	44	170	795	85	86	171	**22601**	
**HRO201**	S2.M	100	6	24	201	93	108	**201**		
**+G1**	S2.M	85	6	23	108	0	**108**			
**+G3**	S2.M	15	0	1	93	**93**				
**p < 0.1**	S13.A	286	44	172	**849**					
**p < 0.01**	S13.B	73	44	**172**						
**p < 0.001**	S13.C	13	**44**							
**HRO286**	S13.G	**286**								

Intersection between different gene data sets (number of identical genes) between any two given gene data sets. Gene set HRO286 and HRO201 described below have been validated by making use of an external data set, here Swiss data set GSE19949.

## Discussion

The analysis of CNAs in genomes of clear cell renal cellular carcinoma underlines that CNAs present in Fuhrman grade G1 and grade G3 tumours differ from each other leading to different CNA signatures.

### Coherence of CNA data sets with previous studies

CNA genes in HRO data match with CNA patterns described before [22, 26, 52, 53]. Concordantly, 557 losses (17 gains) on chromosome 3p were seen in 35/48 tumours together with additional 733 gains (42 losses) on chromosome 5q. Gene losses on chromosome 3p were observed in all 48 tumour samples (Table A in S3 File). Regarding CNA analysis at other chromosomal regions such as chromosome 5q, gene gains of 16/48 (33%) for *TGFBI*, and of 10/48 (20%) for *CSF1R* were in line with previous observations [54–57]. There, *TGFBI* (5q31) was reported to be amplified in 70% of ccRCC cases (7/10) with 5q23.2–q34 gain [54] and *CSF1R* (5q32) to be upregulated in 30% of tumours (3/10) [55]. Soares et al. [56] addressed *CSF1R* involvement in ccRCC pathogenesis based on *CSF1R* copy number gain, overexpression in cancer tissue on mRNA and protein level, plus two novel mutations identified in ccRCC tumours. Upregulation of *CSF1R* gene correlating with 5q gains has been observed by Girgis et al. as well [57]. Recently, novel candidate oncogenes located in distal region of 5q have been discussed, including *PDGFRB*, *STC2*, and *WWC1* [57]. The HRO CNA data reveal gains of *PDGFRB* in 16/48 cases including 12/16 gains of *WWC1* and 8/16 *STC2* (Table D in S15 File). In the study of Beroukhim et al. [58], an integrated analysis of copy-number and expression profiles of ccRCC (n = 48) determined 22 genes located in 5q35.3 region with significant overexpression observed in cancer tissue compared to normal kidney. Twelve of them, including *GNB2L1*, *MGAT1*, *RUFY1*, *RNF130*, *MAPK9*, *CANX*, *CNOT6*, *SQSTM1*, *LTC4S*, *TBC1D9B*, *HNRPH1*, and *FLT4* have been proposed as new ccRCC proto-oncogene candidates. These 12 genes showed at least one gene gain in 19 out of 48 HRO tumour samples (Table D in S15 File). Gains of the complete gene set (n = 12) were present in 3/19 HRO tumours. Eleven genes were amplified in one HRO sample (1/19), 10 in 3/19 tumour genomes (Table E in S15 File). *MYC* (8q24.21) initially reported to be a potential target of 8q gain and a ccRCC candidate oncogene [57, 58] was not found to be amplified, but deleted once in 48 HRO cases. Recently, an integrated analysis of high-density copy number and gene expression data for 54 ccRCC tumours identified *STC2* (5q35.1) and *VCAN* (5q14.3) as potential 5q oncogenes in ccRCCs [59]. Functional studies revealed *STC2* and *VCAN* in ccRCC cell lines to promote cell growth by inhibition of cell death. The HRO data set documented gains of *STC2* in 8 HRO cancer tissues, of which 5 harboured gains of *VCAN* as well (Table C in S15 File). The distribution of 25 CNA genes discussed above were assigned to Fuhrman grades G1 and G3 represented in HRO ccRCC cohort (Table D in S15 File). Fuhrman grade G1 is supported by CNA genes *VHL*, *MGAT1*, *WWC1*, *CSF1R*, *MAPK9* and *TGFBI*. Fuhrman G3 enlisted *CNOT6*, *MET*, *MYC* and *RAF* to be predictive in a small number of G3 tumours.

### Assignments of mutational status of genes associated with CNAs

Conceptually, gene losses are expected to lead to reduced and gene gains to increased levels of gene expression as evinced by published data (Table F in S15 File). In return, gene mutations resulting in loss or gain of functions might mimic or enhance CNA associated phenotypes and vice versa. In case of loss of heterozygosity (LOH), mutations of the remaining allele are most likely going to determine the phenotype. Thus, CNAs have to be put into perspective to mutational frequencies of genes affected by copy number alterations [60]. *SETD2* has been found to be mutated by 11.6%, respectively by 33.5% in ccRCC cohorts analysed by TCGA and by the Memorial-Sloan-Kettering Cancer Center [61]. Haddad and Margulis [42] published a meta-analysis of genes that are preferentially mutated in ccRCC tissues. Interestingly, the most common mutations are seen in genes that as well are predominantly found to be lost in ccRCC genomes of the HRO and Swiss data sets (Table 2). These data are of reasonable relevance most likely indicating that detrimental activities of pre-existing mutations might become enhanced once the corresponding unmutated allele has been lost, see extended data set Table G in S15 File. In the HRO data set the most common 148 CNA genes predominantly lost in at least 25 patients were located on chromosome 3p followed by *ADAM5* on chromosome 8p (Table L in S2 File). Concerning our observation, *VHL* was predominantly found to be lost in Fuhrman grade G1 but retained in several Fuhrman G3 tumours. One might speculate that the *VHL* gene, if present in cases of higher ccRCC malignancies, is either epigenetically repressed instead of being genetically lost or *VHL* mutants dysfunctional in biallelic states do override the functionality of the normal *VHL* allele (Tables A-C in S18 File). Regarding losses of genes in chromosome 3p (Table L in S2 File), subsequent mutations in the remaining *PBRM1* or *BAP1* alleles were expected to result in ccRCC with different pathologic features and outcomes [46], see Table 2 [42, 30, 48, 62–65]

**Table 2:** Fuhrman CNA data of genes that are known to be highly mutated.

	ccRCC clear-cell renal cell carcinoma						ccRCC CNA (HRO)		G1 (26)		G2 (2)		G3 (20)		ccRCC CNA (CH)		G1 (6)		G2 (10)		G3+4 (14)	
Gene	TCGA	Sato et al.	Dalgliesh et al.	Guo et al.	Arai et al.	Scelo et al.	% Loss	% Gain	% Loss	% Gain	% Loss	% Gain	% Loss	% Gain	% Loss	% Gain	% Loss	% Gain	% Loss	% Gain	% Loss	% Gain
SETD2	11.5	11.3	4.4	4.1	9	39.4	89.58		92.3		100.0		85.0		76.7		66.7		90.0		71.4	
VHL	52.3	39.6	51.5	27.6	53.7	73.4	33.33		53.8		50.0		5.0		76.7		66.7		90.0		71.4	
BAP1	10.1	7.5		8.2	6	19.2	58.33		73.1		100.0		35.0		76.7		66.7		90.0		71.4	
PBRM1	32.9	26.4		20.4	32.8	58.5	50.00		65.4		100.0		25.0		76.7		66.7		90.0		71.4	
ERC2					6		56.25		65.4		100.0		40.0		80.0		66.7		90.0		78.6	
RPL14		2.8					41.67		57.7		50.0		20.0		80.0		66.7		10.0		71.4	
RNF123			0.9				35.42		46.2		100.0		15.0		73.3		66.7		90.0		64.3	
MST1	1.4						33.33		42.3		100.0		15.0		73.3		66.7		90.0		64.3	
KIAA0101					6		10.42		11.5				10.0		6.7	3.3			10.0		7.1	7.1
HIF1A			0.9				8.33		3.8				15.0		23.3		33.3		10.0		28.6	
WDFY3						6.4	6.25		7.7				5.0		10.0		16.7				14.3	
MUC4		5.7					6.25		11.5						23.3	16.7	16.7	16.7	30.0	10.0	21.4	21.4
AKAP13				5.1			6.25						15.0		6.7	3.3			10.0		7.1	7.1
ZNF804A				3.1			6.25	14.58	3.8	15.4	50.0		5.0	15.0		6.7				20.0		
ABCA13					7.5			12.50		15.4				10.0		16.7		33.3				21.4
LRP1B				7.1				12.50		11.5				15.0	3.3	6.7			10.0	20.0		
TTN					17.9		2.08	10.42		11.5			5.0	10.0		10.0				30.0		
CSMD3		2.8	2.1	5.1		9.6	2.08	10.42		7.7			5.0	15.0	6.7	10.0	16.7		10.0			21.4
ZNF469						7.5		10.42		11.5				10.0	3.3	6			10.0			
ADAM23					4.5			10.42		11.5				10.0		6.7				20.0		
COL14A1			1.2				2.08	8.33		7.7			5.0	10.0	6.7	6.7	16.7		10.0			14.3
TRRAP						6.4	4.17	6.25	7.7	3.8				10.0	6.7	10.0		16.7	10.0		7.1	14.3
ZFHX4						11.7	2.08	6.25		3.8			5.0	10.0	6.7	10.0	16.7		10.0			21.4
ARID1A	2.9						2.08	6.25	3.8	3.8				10.0	6.7		16.7				7.1	
SLITRK6	1.7						2.08	6.25		7.7		50.0	5.0			3.3						7.1
CARD11				4.1				6.25		7.7				5.0		13.3		33.3				14.3
NAV3				4.1				6.25		3.8				10.0		6.7				10.0		7.1
KMT2D			3.8					6.25		7.7				5.0								
M6PR		3.8						6.25		7.7				5.0								

Most common mutations of genes reported to be preferentially mutated in ccRCC tissues are seen in genes that are predominantly found to be lost in ccRCC genomes of the HRO and Swiss data sets.

### The VHL gene locus

The *VHL* gene showed losses in only 16/48 tumour genomes of which 14 represent grade G1, but only just 1 loss was detected in Fuhrman grades G2 and G3. In line, Patard et al. [66] showed absence of *VHL* mutations to be associated with tumour aggressiveness and poor survival. Interestingly, 12/48 HRO tumours had no visible alteration of the *VHL* locus 3p25.3 besides gene losses at other loci in 3p (Table A in S15 File). Our CNA data on VHL are in line with reports by Yao et al. [67], Kondo et al. [68], Parker et al. [69], Rini et al [70] and Mandriota et al. [71]. According to Yao et al. [67] and Parker et al. [69], *VHL* gene alterations are associated in sporadic clear cell renal carcinoma with better prognosis. Rini et al [70] reported longer Kaplan-Meier time in disease progression in patients with *VHL* mutation or promoter methylation. Genetic changes of *VHL* were reported by Kondo et al. [68] and Mandriota et al. [71] to be an early or first step in ccRCC tumourigenesis rather than a late event. According to our data, losses of *EMC3* (33/48 ccRCC genomes) at 3p25.3 preceded in number losses of *VHL* 16/48 ccRCC tumour at that locus (Table B in S15 File). Our high resolution copy number analysis outlined that CNA genes positioned nearest to *VHL* (16/48 tumour cases) were differentially lost 60kb downstream in 29 ccRCC tumour cases (*FANCD 2*), but 30kb upstream only in 17 ccRCC tumour cases (*IRAK2*). Definitely, future studies are required to determine whether these different assignments of *VHL* gene losses in G1 and G3 ccRCC tumours were due to technical differences in scoring DNA losses or scientifically explainable by accompanied duplication of the remaining chromosomal region eventually resulting in copy-neutral loss of heterozygosity (LOH). The corresponding Swiss data set seems not to separate these genes from each other within the *VHL* locus (Table C in S15 File). Note, gene losses at *VHL* locus favoring less malignant tumour progression are further supported by observations made in von-Hippel-Lindau disease. There, von-Hippel-Lindau tumours of low grade histology showed less likeliness to metastasize and had a better 10-year survival in comparison to sporadic renal cell carcinoma [72]. The latter observation might be related to the fact that secondary mutations of genes associated with losses at the *VHL* locus or protein mutations thereof might contribute leading to more malignant phenotypes in case of sporadic renal cell carcinoma [73].

### CNAs stratifying the Fuhrman classification

CNA genes that do not show any preferences for Fuhrman grading (P-values of p = 1) are assumed to be implicated in common processes of renal tumorigenesis determined by gene sets with 25 gene losses and 203 gains (Table N and O in S2 File). Alternatively, CNA signatures distinguishing grade G1 and grade G3 tumours were recurrently found in a similar and comparable manner in numerous ccRCC tumours (Figs 2 and 3) implying that CNAs signatures are suitable for stratifying ccRCC by distinguishing early from more advanced ccRCC tumour subtypes. Furthermore, Fuhrman G3 ccRCC tumours do not seem to directly originate from Fuhrman G1 tumours since CNA genes initially lost in Fuhrman G1 tumours have to reappear as CNA gains in Fuhrman G3 tumours as depicted in Fig 2. Gene gains found on 7q22.1 and losses on 6q21 seem to lead to more malignant ccRCC grade G3 tumours as exemplified by HRO tumour G3-541 (Fig 4). Though 49 CNA genes located on 3p with p-values of p<0.001 favour G1 phenotypes, additional loci on other chromosomal loci are most likely decisive in manifesting the degree of malignancy. Inspection of individual loci underscore the presence of ongoing chromosomal alterations that most likely obey and follow specific constraints in a non-random fashion (Fig A-F in S4 File).

### Causalities of CNAs

In total, 63592 gene alterations encompassed 15762 genes. On average 1324 CNA events per tumour included 684.8 gene gains and 640.0 gene losses (Table A in S1 File). The appearance of consistent gene losses and/or gains at specific chromosomal loci reoccured in numerous HRO tumours implying the presence of causal mechanisms that are most likely implicated in the pathogenesis and disease-outcome of ccRCC tumours (Figs 2 and 3). Most CNA genes were recurrently detected in a non-random mode exclusively as losses or gains at specific loci in HRO ccRCC tumour genomes. Genes of the mixed type that showed either losses or gains count for 3056 gene members (Table A in S1 File). Overall comparisons of CNAs resulted in ratios of gene losses versus gene gains between ccRCC grades G1 (ratio 1.25) and G3 (ratio 0.58) implying that gains of specific genes are most likely responsible for worse clinical outcomes of Fuhrman grade G3 versus grade G1 ccRCC tumours. Thus, the occurrence of CNAs in ccRCC genomes is definitely not just based on random processes, but reveals the presence of common mechanisms leading to distinct loci-specific chromosomal alterations involved in oncogenic processes (Fig 2 and Fig. G in S4 File)—providing the rationale for CNA based stratification of ccRCC patients. The impact and functional relevance of gene losses and gains on RNA expression levels is underscored by transcriptional data recently obtained in the HAP1 cell system: Expression levels of an originally diploid chromosomal region was shown to be reduced by half after the corresponding region has become haploid by CRISPR-Cas9 engineered intervention (Table F in S15 File), initially published by Essletzbichler et al. [32]. As such, chromosomal losses were inferred to lead to decreasing and gains to increasing expression levels of their corresponding RNA transcripts. Interestingly, of 85 genes whose expressions were reduced in the HAP1 system [32], 23 genes showed copy number alterations detected in 14 ccRCC tumours (6 G1 and 8 G3 tumours). Of 23 CNA genes, 13 CNA gene losses were found in G1, 17 gene losses in G3 ccRCC tumours. The most common loss was assigned to *CSNK1G1* found in 4 grade G1 and 2 grade G3 tumours (Table F in S15 File).

### Functional CNA assessment

Our CNA studies leads to following conclusions: Step I. Initial ccRCC disease processes are dominated by gene losses resulting in Fuhrman grade G1 phenotypes. Step II. Additional ongoing CNAs either establish Fuhrman grade G1 or promote progression to Fuhrman grade G3 tumours. As depicted in Fig. C and D in S7 File, CNA losses and gains are restricted to specific cytobands underlining that specific chromosomal regions confer functionalities implicated in ccRCC disease progression and/or constituting malignancy grades (Table C in S3 File). The open question remains what genetic alterations, mutations or germ line characteristics do initiate chromosomal instabilities as determined herein. Fuhrman grade G1 was dominated by gene losses particularly on 3p (Table K in S2 File) promoting a less malignant phenotype in regard to gene gains at other chromosomal loci that quite often favour more malignant Fuhrman grade G3 phenotypes (Table C in S3 File).

### Validation of HRO CNA gene sets

Our HRO workflow visualized in Fig 1 led to various gene sets displayed in Table 1 such as HRO286 and HRO201. Gene set HRO201 has been validated by using as external data set the Swiss data set GSE19949 previously published by Beleut et al. 2012 [74].

**Fig 8:**
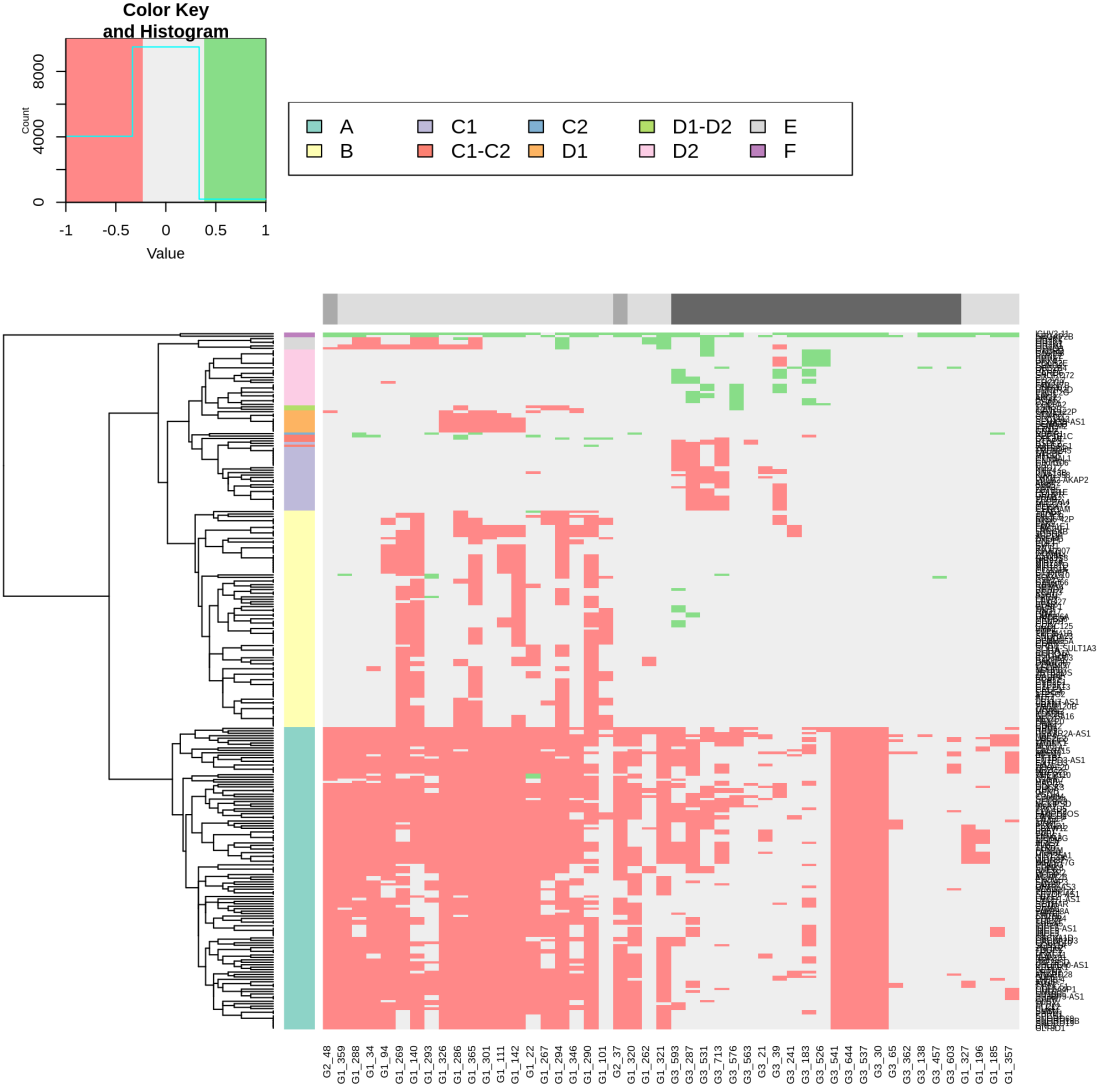
Heatmap of CNA gene sets derived from 4 HRO G3 tumours. Unsupervised hierarchical clustering by average linkage and Euclidian distance of all HRO tumours based on a list of CNA genes with a p-value below 0.1 were shared by 4 G3-patients which are similar to most of the G1-patients (determined by unsupervised hierarchical clustering of the corresponding correlation-matrix, Table A in S13 File). Data are presented at Tables F and G in S13 File.

The HRO CNA data sets derived from 48 ccRCC tumour patient samples provide ideal subsets for comparing CNA gene heterogeneities within and between individual chromosomal loci. The 849 CNA genes that encompassed Fisher’s exact test p-values <0.1 (Table A in S13 File) in combination with the correlation matrix (Fig E in S13 File) stratify three patient groups: Patient Group 1, Patient Group 2 and Patient Group 3 (Fig E and Table F in S13 File). In particular, Patient Group 1, the group with the highest correlation, encompassed 26 tumours (4 Fuhrman grade G3 vs. 23 grade G1) of which CNAs of two G3 tumours even cluster directly together leading to gene set HRO286 encompassing 286 CNA genes. This observation instructed us to further interrogate gene set HRO286 that are shared by these 4 Fuhrman G3 tumours in Patient Group 1 (Fig E in S13 File). This gene set of 286 genes segregated by unsupervised hierarchical clustering in 6 main subgroups A to F leading to group A (losses “G1, G3”), group B (losses “G1”), group C1 (losses “G3”), group C2 (gains “G1”), group D1 (losses “G1”), group D3 (gains “G3”), group E (losses “G1”) and group F (gains “G1”, “G3”) (Fig 8 and Table H in S13 File). The corresponding gene subsets were interrogated to distinguish subgroups of G1 and G3 ccRCC tumours. ccRCC tumour subtypes representing Fuhrman grades G1 and G3 were specified by specific combinations of CNA losses and gains (Table H in S13 File). Losses of group A genes encompassed gene losses of loci on 3p predominantly supporting G1 phenotypes by 2265 CNA gene hits assigned to 26 G1 tumours vs. 648 CNA gene hits present in 20 G3 tumours. Losses of group B genes favoured G1 phenotypes by 558 CNA gene hits in G1 vs. 20 CNA hits in G3 tumours. In particular, gains of group B genes seemed to be supportive for Fuhrman grade G3 tumours. Losses of group C1 genes stratified G3 tumour phenotypes by 92 G3 CNA gene hits vs. 1 G1 CNA gene hit. Gains of group C2 genes favoured G1 phenotypes due to CNA gain of *NDFIP1*. Losses of group D1 genes supported G1 phenotypes by 51 G1 CNA gene hits vs. 1 Fuhrman G2 and no G3 CNA gene hits whereas gains of group D2 genes specified G3 phenotypes by 70 G3 CNA genes vs. 1 G1 CNA gene hit. Losses of group E1 genes supported G1 phenotypes. The combinatorial analysis of CNA gene losses and gains in gene set HRO286 demonstrates that criteria can be determined for subclassifying ccRCC tumours (Fig 8). A subset of HRO tumours representing Fuhrman tumour grades G1 and G3 can be related to CNA gene gains and gene losses of 15762 genes broken down to 286 CNA genes (HRO286), see Supplement S2 Text.

### Patient stratification by gene set HRO201

**Fig 9:**
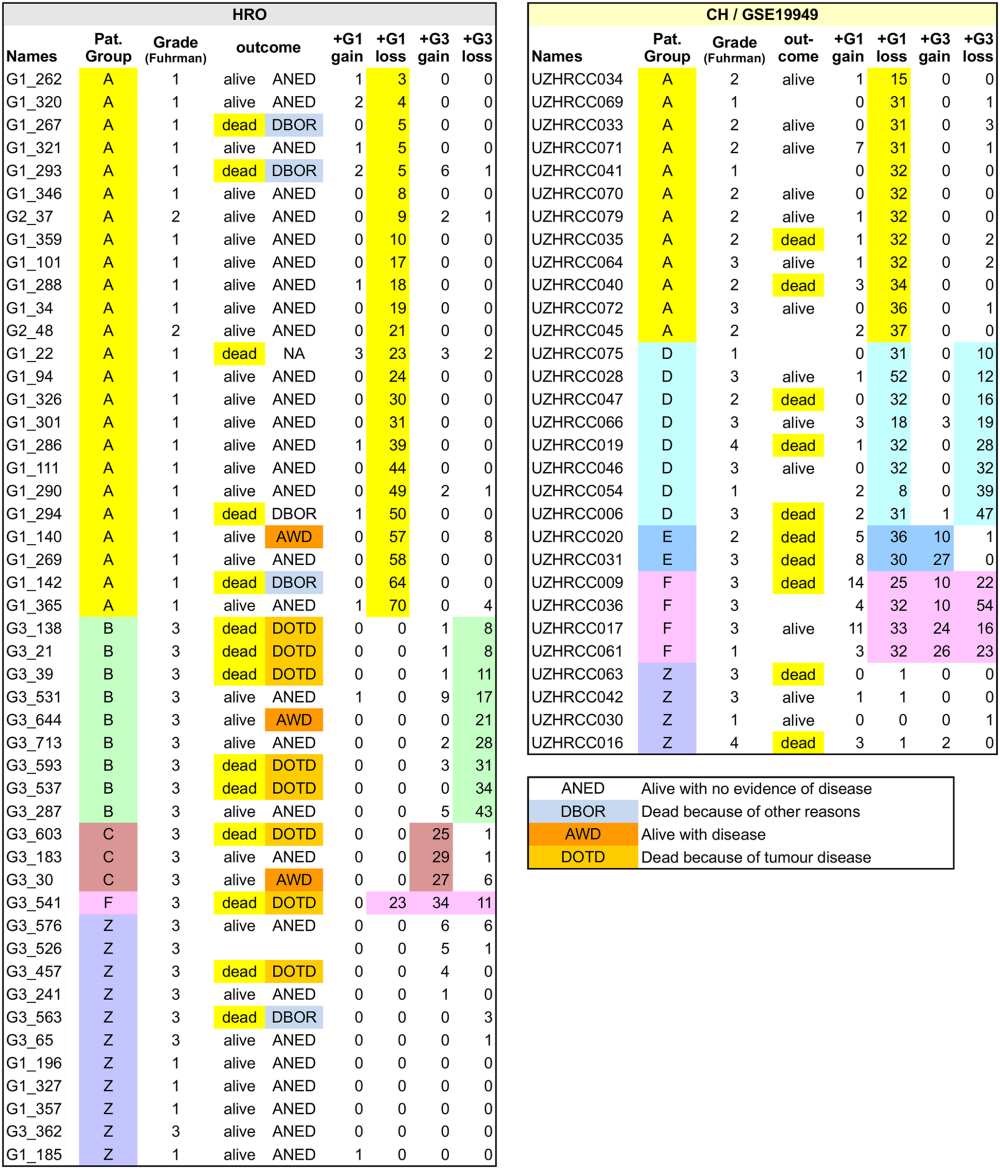
Patient group assignments of HRO and Swiss data sets. CNA based patient group assignments include Fuhrman grades and survival outcome. Classifications are highlighted (extended data under S16 File).

**Fig 10:**
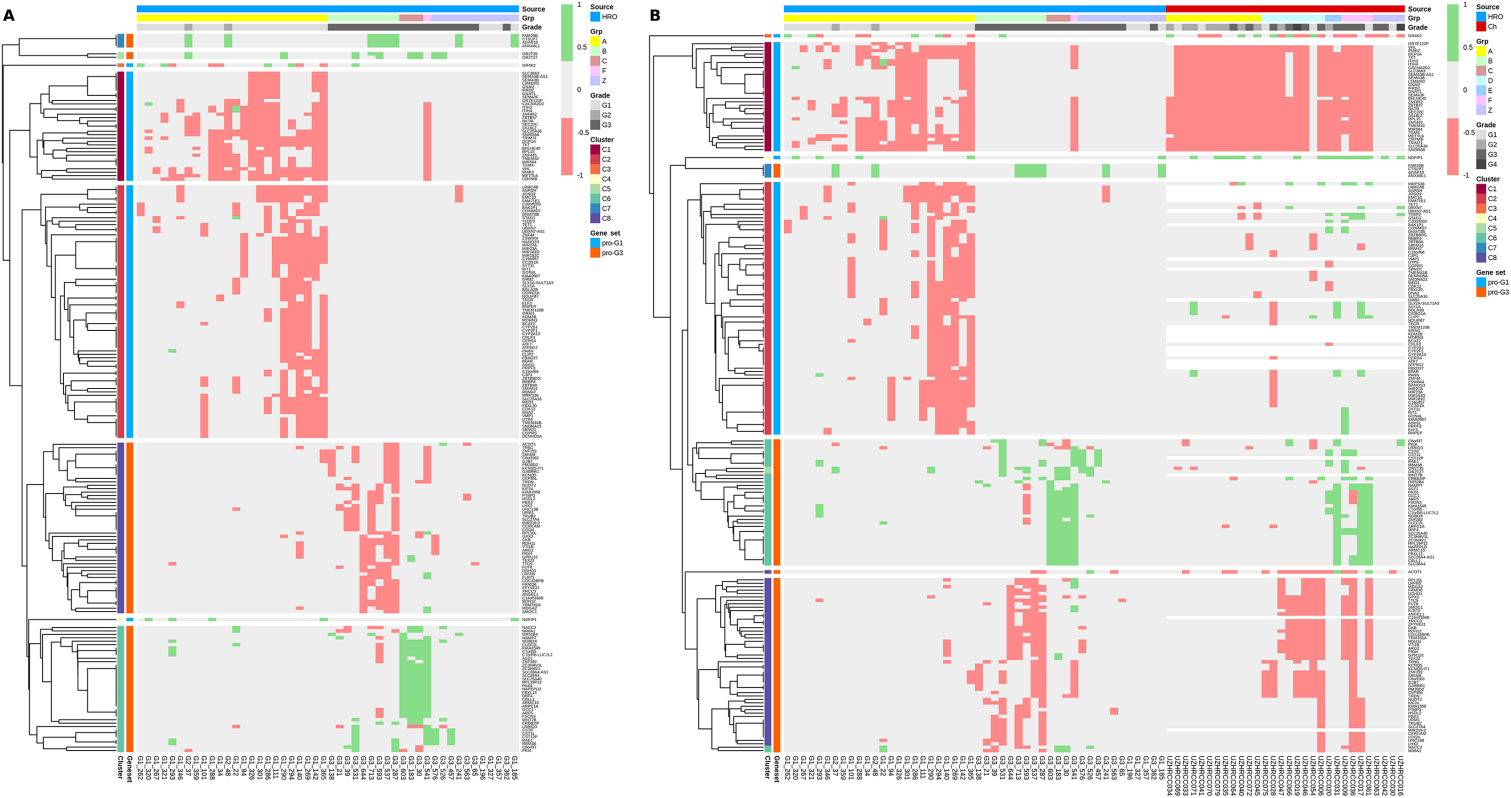
CNA-heatmap of HRO and Swiss tumours (gene set HRO201). A: Hierarchical clustering of CNA genes (gene set HRO201) regarding HRO is shown. In total, 8 gene subgroups are obtained by unsupervised hierarchical clustering by average linkage and Euclidian distance, data at Table A and B in S14 File. Corresponding patient groups as well as Fuhrman gradings are shown, see Fig 9 as well. B: Swiss data set (GSE19949) was analysed as in Fig 10A based on Gene set HRO201. Data at Table A and B in S16 File. Concerning HRO and Swiss tumours, corresponding patient groups as well as Fuhrman gradings are shown, see Fig 9 as well.

HRO and Swiss tumour genomes were compared by assigning G1 and G3 CNA gene losses and gains as initially specified by gene set HRO201 to individual ccRCC tumours of both cohorts (Table A in S16 File). Mutual ccRCC subtypes were identified that show subtype-specific signatures (Fig 9). Stratifyed the HRO cohort, patient group A was predominantly specified by G1 gene losses, patient group B by G3 gene losses, patient group C was identified by G3 gains and patient group F by a combination of G1 losses, G3 losses and G3 gains. CNA gene set HRO201 stratifies the Swiss cohort in a similar manner (Table B in S14 File). Interestingly, the Swiss patient group D turned out to be a composite of HRO patient group A and B, and the Swiss patient group E a composite of HRO patient group A and C. One reason why HRO patient groups B and C were not found as singular signatures in the Swiss cohort might be due to different degrees of tumour heterogeneities present in tumour tissues. CNA differences between both cohorts are possibly due to size and shape of tumour tissue areas being taken for initial DNA extraction. Heterogeneities of HRO tumours seems to be less complex in the HRO data set that displays a lower average of copy number alterations per patient (Table A in S1 File). Gene set HRO201 was insufficient in stratifying ccRCC patients designated patient group Z (Table A in S16 File) most likely due to lack of appropriate discriminatory CNA genes within the data set. Thus, as a result of our combined cohort analysis patient group A tumours are dominated by G1 related gene losses, patient group D tumours have G1 and G3 associated losses in common, whereas patient group B predominantly harbours increasing numbers of G3 related gene losses and patient group C genes are classified by G3 related CNA gene gains. Patient group E describes tumours having G1 related losses and G3 related gains in common. Patient group F tumours are specified by G1 related gene losses and gains plus G3 related gene losses. HRO and Swiss ccRCC tumours have been stratified based on G1 and G3 associated CNA losses plus G3 related CNA gains (Fig 10A). Interestingly, rates of deaths seem to increase with the total number of G3 related gene losses plus the number of gene gains (Table A in S16 File). The comparative analysis of HRO and Swiss CNA data sets using gene set HRO201 indicates that mutual CNA patterns are repeatedly present in both renal cancer cohorts underlining that chromosomal instabilities follow a specific order (Fig 10B). Overall, the HRO201 gene set is suitable to coherently stratify ccRCC patients in a comparable manner as initially identified by correlation matrix analysis (S1 Fig) and visualized by hierarchical clustering (Fig D in S14 File) stratifying ccRCC tumour subtypes present in both patient cohorts.

### Comparative analysis of gene sets HRO286 and HRO201

Gene set HRO201 was successfully applied to stratify both ccRCC cohorts, the HRO (201 CNA genes) as well as the Swiss (171 CNA genes), in a mutual manner (Table B and C in S14 File). Interestingly, Swiss patient groups D and E describe composites of the original HRO patient groups A and B, as well as A and C, respectively (Fig 9). The HRO gene set HRO201 is capable of stratifying both ccRCC cohorts, the HRO as well as the Swiss. In particular, the workflow how gene set HRO286 was established (S2 Text) exemplifies how to determine subsets of CNA genes that match individual ccRCC patients having distinct CNAs in common. In particular, gene set HRO286 that originated from gene sets of 4 G3 tumours related G1 tumours (Fig E in S13 File) seems to be limited in its discriminatory power in comparison to gene set HRO201 for distinguishing Fuhrman G1 and G3 ccRCC tumours. Gene set HRO286 shares many CNAs in common with numerous G1 tumours. Thus, gene set HRO286 does most likely not include a sufficient number of discriminatory G3 related CNA genes in order to stratify higher malignant ccRCC tumours more efficiently. Apparently, higher ccRCC stages might go along with progression in tumour size and tumour heterogeneity [75]. Possibly, quite distinct CNA patterns might occur at these tumour stages that might not reflect CNA gene sets herein identified in G1 and early G3 tumours. Furthermore, external and internal CNA data sets are considered to be of limited comparative usability if the complexity of CNAs including SOPs for CNA data generation does significantly diverge from each other (Table A in S1 File).

### Overall survivals and Kaplan-Meier Analysis

**Fig 11:**
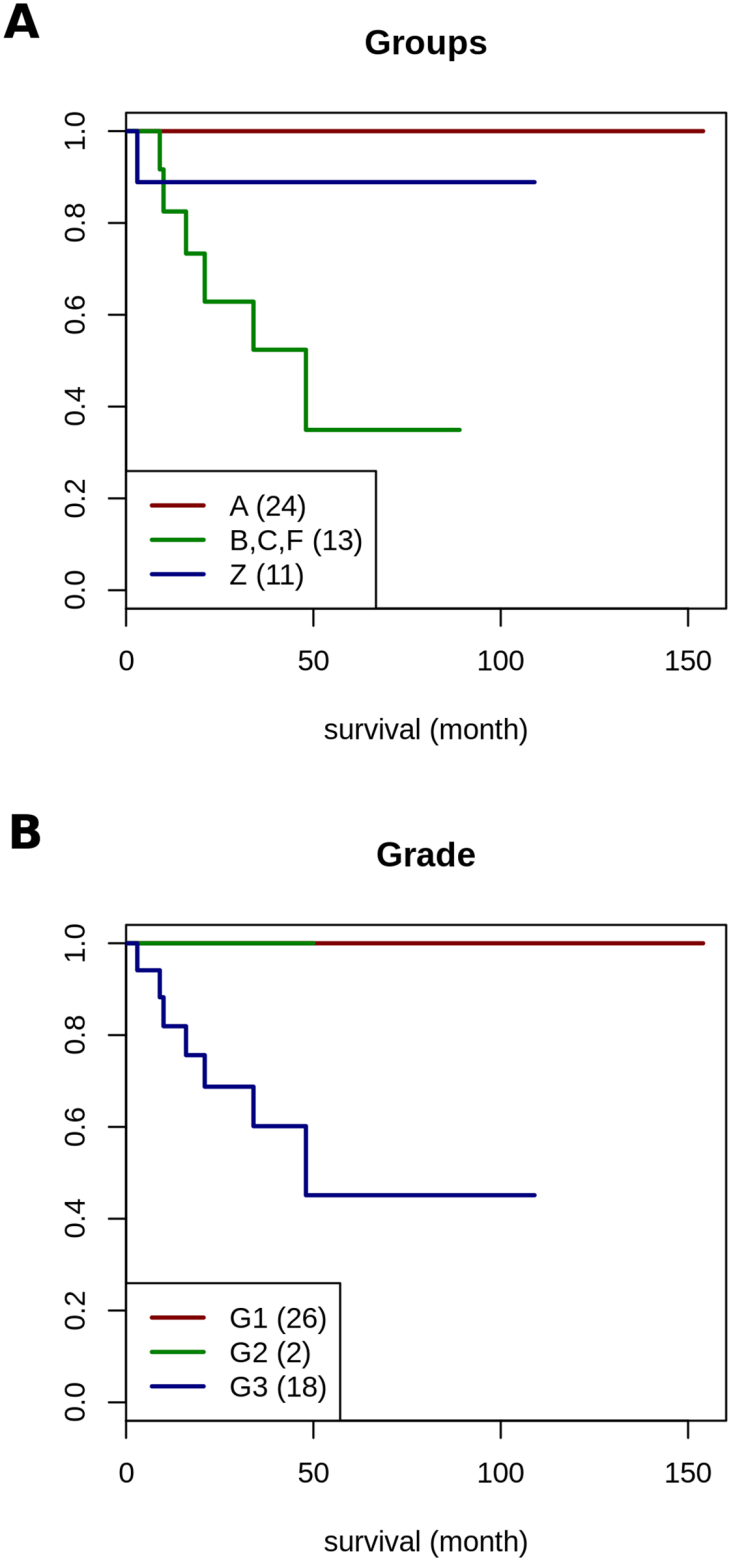
Kaplan-Meier survival analysis of HRO cohort patients. A: visualizes survival data of HRO patients stratified by three CNA patient groups based on gene set HRO201 leading to classifications as assessed in Table A in S16 File. The hazard ratio (HR) of the HRO cohort concerning both two groups (A, Z vs. B-F) is 21.76 (95% confidence interval is [2.58, 183.2]). The logrank test between both two groups yields a p-value of 5.83·10^−5^. B: is based on survival data categorized by Fuhrman grades G1, G2 and G3, number of patients in parenthesis (data: Tabla A in S16 File). Comparing Fuhrman grades G1 and G3 using Cox proportional hazards regression model leads to a hazard ratio close to infinity since all G1 and G2 patients are survivors.

In many cases, documentations of overall survival data do not distinguish between deaths of tumour disease (DOTD) and deaths because of other reasons (DBOR). Quite often, overall survival data generated of ccRCC patients are limited in their informative value due to the fact that grading and staging are based on tumour status determined at the time of surgery, but not at the date of death. Thus, the cause of death is quite often not reliably documented in respect to the cause of death, cancer related versus unrelated (Fig 9). As such, the value of Kaplan-Meier analysis is even more limited due to the fact that Fuhrman grades determined in primary kidney tumour material do not necessarily reflect Fuhrman grades present in metastases that are quite often associated with tumour related deaths. Kaplan-Meier analysis using gene set HRO201 applied on HRO patient data visualized that patient group A had the best overall survival in comparison to the composite of patient assignments of patient groups B, C and F (Fig 11). Unfortunately, the Swiss patient data do not distinguish between tumour related deaths (DOTD) and deaths because of other reasons (DBOR). The HRO201 gene set based classification seems to identify Swiss patients with higher tendencies and frequency to die on tumour disease (Table B in S16 File). Thus, specific CNA gene losses and gains evinced to be related to overall survival of ccRCC patients. Consequently, CNA genes involved are considered candidate genes to be preferentially analysed, i.e. for methylation patterns, gene mutations, expression levels and associated gene functions on the way to determine causal mechanisms driving oncogenic processes in ccRCC tumour malignancy progression.

## Final statements

Initial correlation maps indicate the presence of CNA patterns either reflecting common features regarding the distribution of losses and gains of CNA genes and/or their contribution to Fuhrman malignancy grades. Informative ccRCC subgroups were identified with the aim to determine common nominators including causal relationships that initiate CNA related processes and govern an ongoing progression of CNAs. Finally, distinct gene sets thereof have been validated by taking advantage of additional CNA data sets. Herein, CNA data of a Swiss ccRCC cohort [74] publically available under GSE19949 (designated Swiss cohort) were successfully applied for validating CNA gene sets initially determined by copy number analysis of 46 ccRCC tumours at the University Medicine, Hansestadt Rostock (HRO), designated HRO gene sets. Our initial results provided further evidence indicating CNAs are suitable for stratifying ccRCC tumour genomes. The workflow presented has been demonstrated to lead to informative CNA signatures of ccRCC subsets worth to be further investigated by exome, RNA and epigenetic analyses in order to evaluate the diagnostic and therapeutic impact of individual CNA signatures (Fig 1). Tumour processes of ccRCC are considered to be due to an interplay of mutational changes possibly occurring stochastically in combination with orchestrated copy number alterations propagated by causal regulatory processes. Our CNA studies of ccRCC tumour genomes enable the categorization of ccRCC genomes and their subclassification. Distinct chromosomal loci reveal the existence of ordered processes of gene losses or gains associated with different stages of tumour progression. These alterations became visible by aligning CNAs present in corresponding cytobands. The CNA analysis substantiates our understanding of causal mechanisms associated with gains and losses of genes possibly driving or reducing tumour progression. The order of specified CNA gene gains and losses identified by studying CNAs of ccRCC at distinct chromosomal loci distinguishes chromosomal aberrations between early and late CNA processes in oncogenesis of renal cancer (Figs 3, 4 and 5). Thus, our HRO CNA data set documented an ideal starting point to determine a time line how the interplay of distinct gene gains (e.g. amplification of tumour driver genes) and/or losses (e.g. tumour suppressor genes) at specific chromosomal loci influence the progression of ccRCC tumour formation. Hereto, CNAs of particular HRO tumours were ordered beginning with initial CNA events followed by additional losses and gains at these loci (Fig C and G in S4 File). These studies are considered to become a starting point for initiating forthcoming CNA studies in concert with genomic sequencing analysis guided by CNA results enlisted in Table 1. Our ongoing studies are predominantly focussed on investigating early G1 ccRCC tumours that encompass low numbers of gene losses and gains. Clear cell renal cell carcinoma (ccRCC) tumour genomes of this kind are considered to be instrumental in defining somatic or even germ line mutations that are involved in initiating and/or mediating chromosomal aberrations. The type of CNA signature assigned to each individual ccRCC patient is foreseen to significantly contribute to the understanding of ccRCC pathogenesis and to guide further ongoing studies. The overall challenge will be to determine the prognostic and predictive power of specific CNA gene sets in substratifying ccRCC patients.

### Perspectives

Our CNA analysis prioritized distinct low and high malignant ccRCC tumours that deploy low numbers of copy number alterations to be subjected to whole genome sequence analysis in order to determine causal gene mutations involved in initiating chromosomal instabilities in clear cell renal cellular carcinoma. The application of genome-wide analysis of copy number alterations in concert with forthcoming whole genome sequencing data is expected to provide significant progress in biological understanding of ccRCC pathogenesis and oncogenesis in general. As indicated herein and stated elsewhere [76] detailed studies of selected CNA signatures are assumed to lead to more robust tumour classifications hand in hand with exome and RNA based transcriptome data sets. One tempting challenge will be to define molecular features that initiate and promote chromosomal alterations in ccRCC tumour panels. Our key efforts in complementing ongoing research [77] is focussed on whole genome sequence analysis of ccRCC Fuhrman G1 tumours that display a limited number of copy number alterations. Thus, it will become tempting to search for putative causal mutations such as point mutations or small deletions or insertions by exome or by advanced whole genome sequence analysis that initiate and propagate chromosomal instabilities. The final challenge will be to relate dynamics of mutations, translocations, copy number alterations and epigenetic modifications occurring in ccRCC to clinical outcome. According to our own analysis, each ccRCC tumour subtype displays common and cancer subtype-specific copy number alterations (CNAs) possibly guiding or guided by mutational alterations (Table 2). Our workflow of how to determine CNA gene sets possibly involved in initiating and propagating CNAs might be applicable to other tumour entities as well. Gene-directed and patient-centered CNA analyses have been applied to stratify renal cancer genomes with the perspective to determine nominators that are expected to support clinical assessments and lead to more promising treatment options as well as improved clinical outcome.

## Materials and methods

### Clinical data

In total, the HRO ccRCC data set represents 48 ccRCC patients that underwent surgical treatment by radical or partial nephrectomy at the Department of Urology, University Medicine Rostock, Hansestadt Rostock (HRO) in a three year period from 2009 to 2011. Assessment of histological gradings performed by certified pathologists resulted in 26 Fuhrman grade G1, 2 Fuhrman grade G2 and 20 Fuhrman grade G3 ccRCC tumours.

### Sample processing and CNA profiling

At the time of surgery, tissue samples were collected, freshly frozen and stored in liquid nitrogen at -80°C. Genomic DNA was extracted from small frozen pieces of RCC tissue (close to 30mg tissue) according to the tissue protocol for the QIAamp^®^ DNA Mini Kit (Qiagen, Hilden). Agarose gel electrophoretic separation was performed for quality control after spectrophotometric quantification using the Nanodrop 1000 device. The DNA samples were treated mainly as described in the “Affymetrix Cytogenetics Copy Number Assay User Guide”, i.e. 500ng genomic DNA sample was split and two aliquots of 250ng were cleaved by restriction endonucleases (StyI and NspI). After adapter ligation, a reduction of the genomic complexity was performed by limited cycle preparative PCR. PCR products were cleaned up using an ultrafiltration procedure by NucleoFast 96 PCR Plates (Machery-Nagel). Fragmentation by DNase I and end labelling was done using the Genome-Wide Human Nsp/Sty 5.0/6.0 Assay Kit (Affymetrix). The hybridization of Genome-Wide Human SNP 6.0 Arrays followed for 16 to 17 hours at 50°C in the GeneChip Hybridization Oven 640. After washing, staining and antibody amplification using the Fluidics Station 450 the SNP 6.0 arrays were scanned with the Affymetrix GeneChip Scanner 3000 (7G) at 0.7 micron resolution. Data processing: Cel-files, representing raw image data of Genome-Wide Human SNP 6.0 Arrays, were processed by the Affymetrix Genotyping Console (Version 4.2.0.26) with a reference file calculated locally based on 270 HapMap samples supplied by Affymetrix NetAffx annotation files NA33. Genotyping was performed by Birdseed_v2 algorithm and segmentation using default settings, minimum number of markers per segment = 5, minimum genomic size of a chromosomal segment = 100 kilobase pairs (100kbp), and an overlap of known CNA regions by 100%. Segmentation files listing initial information on every called segment per tumour were annotated using R package ChIPseeker [78] with TxDb.Hsapiens.UCSC.hg19.knownGene as genomic background. In a comprehensive manner, data sets derived from KEGG [79], COSMIC (v71) [80], TSGene [81] and IntOGen [44] were used for CNA stratification and gene classification.

### Statistical analysis of CNA Data

Gains and losses determined on 26 G1 and 20 G3 ccRCC tumour samples led to a gene set of 15762 different genes. The workflow is visualized in Fig 1. R version 3.2.3 (http://www.r-project.org) was used for comparative statistical data analysis. Fisher‘s exact test for count data was employed to determine the discriminatory power of each CNA gene in respect to the Fuhrman grade assignments G1, G2 and G3. A p-value of p<0.1 led to a CNA gene list of 849 genes (Table A in S13 File). Fisher‘s exact test for count data was taken as well to determine the discriminatory power of chromosomal bands specified by CNA gene losses and gains in respect to Fuhrman grading. CNA gene lists were determined by correlation matrix analysis conducted by R version 3.2.3 as well. Networks were visualized by cytocape [82]. Data validation was conducted by analysing published Swiss data [74] available under GSE19949 leading to gene set HRO201 and restricted HRO gene set of 171/201 CNA genes based on overlapping CNA genes with the Swiss cohort as presented in Table 1.

#### Gene set HRO286

As a result of comparative correlation analysis between HRO G1 and G3 ccRCC tumours, 286 CNA genes have been identified that are completely shared by 4 Fuhrman G3 (127 CNA genes) in conjunction with 20 grade G1 related gene losses (Table F and G in S13 File).

#### Gene set HRO201

The relative number of patients regarding individual copy number alterations (CNA) of each CNA gene per group (G1, G3) is calculated and those ratios are then subtracted:

$$\text{R}=\frac{\left(\text{G}1\text{ patients with CNA}\right)-\left(\text{G3 patients with CNA}\right)}{\left(\text{G}1\text{ patients with CNA}\right)+\left(\text{G}3\text{ patients with CNA}\right)}$$

These ratios are determined in conjunction with two additional constraints: I. Gene Set I: Each CNA gene altered in at least 4 G3 patients with a CNA ratio of -⅔ or less defines 93 CNA genes with "G3>G1", and II: Gene Set II: Each gene being altered in at least 5 G1 patients with a CNA ratio of ⅖ or more enlists 108 CNA genes with "G1>G3".

#### GEO GSE19949 data

Swiss data set GEO GSE19949 [74] was downloaded from https://www.ncbi.nlm.nih.gov/geo/query/acc.cgi?acc=GSE19949. Fuhrman assignments and overall survival data were provided by Peter Schraml, Department of Pathology and Molecular Pathology, University Hospital Zürich.

#### GEO GSE95239

The HRO data discussed in this publication have been deposited in NCBI's Gene Expression Omnibus and will be accessible through GEO Series accession number GEO GSE95239.

### Kaplan-Meier analysis

Cancer-specific survival has been determined by Kaplan-Meier analysis. The associations of assigned patient groups and overall survival with ccRCC-specific deaths was estimated using Cox proportional hazards regression model for both groups (A, Z vs. B-F). The hazard ratio (HR) of the HRO cohort concerning both two groups (A, Z vs. B-F) is 21.76 (95% confidence interval is [2.58, 183.2]). The logrank test between both two groups yields a p-value of 5.83·10^−5^. Regarding the Swiss data set, hazard ratio concerning both Swiss groups (A, Z vs. B-F) is 2.47 with 95% confidence interval at [0.69, 8.80] and a logrank test p-value of 0.151.

## Ethics statement

All patients provided written informed consent that they agreed to undergo surgery of the kidney in order to remove their kidney tumours. This consent comprised the subsequent pathological investigation of the renal specimens. Since the tumour specimens have been analysed retrospectively using that routine pathological material, the participants' informed consent covered our analyses. The study and this consent procedure have been approved by the ethics committee of the Medical Faculty at the University Medicine Rostock.

## Supporting information

S1 File Abundance of CNA gene gains and losses in ccRCC cohorts. A: CNA gene gains and losses in HRO, Swiss and TCGA ccRCC cohorts are given as absolute numbers of altered CNA genes in total and per patient (groupwise averaged by gradings). B: Sample IDs, Fuhrman gradings, total number of CNA gene gains and losses, "mixed type" genes (described as gene gains or losses) and the number of pathways affected categorize each individual HRO tumour. C: Sample IDs, Fuhrman gradings, total number of CNA gene gains and losses, "mixed type" genes (described as gene gains or losses) and the number of pathways affected categorize each individual Swiss tumour. Additionally to Fuhrman gradings, Thoenes grades are given for this cohort. The latter grading is available as uploaded data set at GEO GSE19949. For enabling the comparison of CNA datasets with tumour gradings, Fuhrman gradings have been obtained directly from Peter Schraml, coauthor of Beleut et al. 2012 [74]. D: Top 6 KEGG-pathways are determined that contain the highest numbers of genes showing CNAs. Enlisted are the numbers of genes in individual pathways, numbers of genes altered in at least one tumour, the relative amount of genes in the corresponding pathway, the number of tumour-suppressor- and driver-genes in that pathway and cumulative numbers of CNAs, distinguished by type of CNAs (Losses, Gains) and gradings (G1, G3) as well as the number of affected tumours.
(XLSX)

S2 File CNAs of 15762 genes. S2 subtables are presented in similar formats: the first 3 columns enlist gene names and chromosomal localization. Column D & E show the number of tumours with gains or losses of individual genes. Column F enlists the number of KEGG-pathways linked with each CNA gene. Column G presents p-values of Fisher's exact test (testing whether this gene differentiates between G1 and G3). Columns H to N contain numbers of tumours, according to Fuhrman gradings in the header. Column O describes the relative number of tumours affected by CNAs of individual genes. P and Q describe the relation between number of affected G1 and G3 tumours and column R the difference between both. Columns S to AG contain a "1" if a gene is assigned to a specific set of genes or a specific pathway. The first Table contains all CNA genes of the HRO cohort, the other tables contain identical data but enlist specific subsets of CNA genes.
(XLSX)

S3 File A: Regarding all HRO tumours, CNA gene losses at 3p25.3 are enlisted in column C and associated gains at loci at 5q Loci at 5q are enlisted in columns F to AJ. B: CNA gene losses at 3p25.3 are enlisted in column C and associated gains at loci at 5q Loci at 5q are enlisted in columns F to AJ restricted to HRO tumours having at least losses or gains in one both chromosomal loci.C: CNA data of 2 sets of genes: G1 nominator genes and G3 nominator genes regarding each HRO tumour are enlisted: gene losses are designated “-1”, gains “1” and no CNAs “0”.
(XLSX)

S4 File CNA gene assignments to chromosomal loci. CNA gene assignments to chromosomal loci visualizing Fuhrman grades G1 and G3.A: Cytoband-plot of 3p21.31 is shown for all HRO tumours. Bars consist of up to 2 colours, representing the number of tumours assigned by Fuhrman grades G1 and G3: medium gray represents G1 and dark gray G3. B: Cytoband-plot of 16p13.3 representing all HRO tumours. C: Cytoband-plot of 14q32.33 representing 8 different HRO tumours. The height of bars documents number of tumours that share CNAs. Assignments of colourings are dark = G3 nominator genes, medium = G1 nominator genes, light = genes without any preference for Fuhrman malignancy grades. D: Cytoband-plot of 14q32.33 representing all HRO tumours. E: Cytoband-plot of 7q22.1 representing all HRO tumours. F: A cytoband-plot (like A) representing locus 16p11.2 G: Cytoband-plot of locus 4p16.3 showing only CNAs of 7 selected HRO tumours.
(PDF)

S5 File CNA genes assigned to cytobands. Each cytoband is characterized based on following information: Number of genes at each cytoband (column B), number of genes showing only losses (column C), number of genes showing only gains (column D), number of genes showing both gains and losses (column E), number of affected tumours by all genes in this cytoband (column F), number of CNA genes only in G1 tumours (column G), number of CNA genes only in G3 tumours (column H) and number of CNA genes in G1 and G3 tumours (column I).
(XLSX)

S6 File Correlation matrix analysis of tumour samples. Unsupervised hierarchical clustering by average linkage and Euclidian distance, representing a correlation-matrix, show Pearson correlation between tumours, based on: A: CNA losses per cytoband; B: CNA gains per cytoband; C: CNA losses per cytoband but restricted to highly altered cytobands affected by at least 20 tumours; D: CNA gains per cytoband but restricted to highly altered cytobands affected by at least 20 tumours. E: CNA losses per cytoband but limited to specific cytobands (p-value below 10^−14^). Colour-code follows as: High positive correlation is represented by green and high negative correlation by red.
(PDF)

S7 File Correlation matrix analysis of cytobands. Unsupervised hierarchical clustering by average linkage and Euclidian distance, representing a correlation-matrix, show Pearson correlation of: A: losses between all cytobands; B: gains between all cytobands; C: losses between highly altered cytobands affected by at least 20 tumours; D: gains between highly altered cytobands affected by at least 20 tumours;E: gains between significant cytobands (p-value below 10^−14^). Colour-code follows as: High positive correlation is represented by green and high negative correlation by red.
(PDF)

S8 File Selected chromosomal loci specified by CNA gene assignments. These tables contain ten subtables that categorize individual genes at specific chromosomal loci enlisted in a format similar to S2 File and one overview-table of those 10 chromosomal loci.
(XLSX)

S9 File CNA genes present in at least 20 tumours (Top 20). A: enlists all CNA genes present in at least 20 tumours (columns described alike S2 File), subdivided into 3 subtables: B: contains only CNA genes with losses in at least 20 tumours but no CNA gains in any of these tumours, C: contains at least 20 HRO tumours having a gene gain but no one a gene loss, and D: contains CNA genes that are lost in some HRO tumours and gained in others.
(XLSX)

S10 File Pathway interaction networks of KEGG pathways. A: All pathways of assigned CNAs present in at least 20 HRO tumours are visualized by Cytoscape [82], see data Table A in S11 File. B: Most common 11 pathways of assigned CNAs present in at least 20 HRO tumours are visualized by Cytoscape [82], see data Table B in S11 File.
(PDF)

S11 File Pathways of assigned CNAs present in at least 20 HRO tumours. CNA genes enlisted show CNAs in at least 20 HRO tumours assigned to at least one KEGG-Pathway. "1" describes the linkage between CNA genes and associated pathways. A: enlists all KEGG pathways. B: enlists the most common KEGG pathways. Column N depicts the sum of pathways regarding each CNA gene (i.e. the number of pathways linked with that gene). Furthermore, column O and P mark whether a CNA gene is a driver or a tumour suppressor gene.
(XLSX)

S12 File PI3K-pathway decomposition. Decomposition of the KEGG Pathway PI3K representing tumour A: G1_365; B: G3_287 shows a colour coded map of genes, see as well Fig 7 of the manuscript. The shapes are:
Red = LossBlue = GainYellow = Both states (Gain/Loss)Rectangle = Uncoloured, gene is not affected by copy number alterationRectangle = Grade (1/3) equally affected, colour shows aberrationCircle = Grade 3, colour shows aberrationDiamond = Grade 1, colour shows aberrationCorresponding data are enlisted at Table G in S2 File.
(PDF)

S13 File A-C: enlist CNA genes based on Fisher’s exact test with p-values representing thresholds with p-values of <0.1; <0.01; <0.001). Columns enlisted are alike S2 File. D: enlists genes that distinguish Fuhrman G1 and G3 tumours of Patient Group 2. Subgroups are visualized by correlation matrix analysis (E). E: Hierarchical clustered heatmap of a correlation-matrix showing the correlation between HRO tumours based on CNA losses per gene specified by p-values below 0.1 (A). Three patient groups are determined. Four G3 tumours stratified by patient group 1 lead to gene set HRO286 (Fig 8, G, H). This correlation-matrix-analysis uses a modified weighted matrix: negative numbers still represent a loss and positive numbers a gain but instead of -1 and 1, numbers of HRO patients showing losses and gains of the same gene replaces the original numbers. Below the complete Heatmap is a reduced image referring to patient group 2 depicted in Fig 6 visualized only CNA genes that differ between Fuhrman G1 and G3 tumours of that subgroup (patient group 2). Corresponding CNA gene are enlisted in D. This correlation-matrix-analysis uses a modified weighted matrix: negative numbers still represent a loss and positive numbers a gain but instead of -1 and 1, numbers of HRO patients showing losses and gains of the same gene replaces the original numbers. F: Description of patients groups 1, 2, 3 determined in E. HRO tumour IDs are assigned to patient groups 1, 2, and 3 depicted in E. Four G3 tumours stratified by patient group 1 lead to gene set HRO286 (Fig 8, G, H). G: CNA genes selected based on a fisher's exact test with p-value <0.1 display identical CNAs in 4 HRO tumours G3_644, G3_537, G3_541, and G3_30. Gene groups (Column AY) are determined by hierarchical clustering. This table displays the exact data of Fig 8.H: CNA genes describing individual gene groups are documented in a different format. Gene groups are visualized in the manuscript Fig 8. Data are enlisted in G.
(XLSX)

S14 File Gene set HRO201 in respect to HRO and Swiss cohort data. A: Gene set HRO201 differentiates HRO Fuhrman G1 and G3 tumours. Swiss cohort data are enlisted as well. Columns are: Gene name (column A), Chromosome (column B), Cytoband (column C), Pathway affiliation (columns D-G), Subgroup (column H, whether CNAs of genes are more likely G1-related than G3-related). The rest of the columns describe the data sets. See as well Table B and C in S16 File and Fig 10 in the manuscript. B: This table encloses raw data used to generate the correlation-matrix leading to figure S1 Fig regarding the assigned patients-groups (Fig 10 enclosed in the manuscript and Table A in S16 File). Patient IDs of ccRCC tumours studied in Table C and Figure D in S16 File and S1 Fig are coloured in yellow in Table B in S14 File. C: This table encloses raw data used to generate the correlation-matrix leading to S1 Fig representing restricted patient-groups. D: This CNA heatmap generated by unsupervised hierarchical clustering (average linkage and Euclidian distance) represents CNA HRO201 gene assignments by displaying a subset of HRO and Swiss tumour samples (see colouring of patient IDs in Table B in S14 File) in contrast to all HRO ccRCC tumours and to both HRO and Swiss tumour data sets (Fig 10 of the manuscript).
(XLSX)

S15 File CNA gene losses in order of chromosomal locus 3p25.3. Table A enlists all HRO CNAs present in cytoband 3p25.3 stratifying all individual 48 HRO patients. Additionally, HRO samples that do not show any gains on 5q are highlighted. Table B summarizes CNA data of 3 p25.3 alike tables in S2 File. Table C alike Table A enlists all Swiss CNA data concerning ccRCC tumours at 3p25.3. Table D enlists CNA genes predominantly positioned at 5q35.3. CNA gains and losses differentiating Fuhrman grades G1 from G3 are shown in conjunction with KEGG pathway assignments. Columns C to K assign CNA subsets to numbers of HRO tumours as described by the header. Table E enlists CNA genes exclusively positioned at 5q35.3. CNA gains and losses differentiating Fuhrman grades G1 from G3 are shown in conjunction with KEGG pathway assignments. Columns C to K assign CNA subsets to numbers of HRO tumours as described by the header. Table F shows CNA gene assignments to genes at 5q21-26 regarding ploidy and RNA expression. First column enlists an arbitrary index, columns B-G gene names and chromosomal localizations. H-M contain expression data from cell lines A11 (haploid), E9 (haploid) and C597 (diploid) including 2 replicates per cell line. Columns N-P contain averaged values of each cell line plus in columns Q-S statistical data regarding all 3 cell lines. Columns T-V enlist relative values (expression-distribution between these 3 cell lines). The original data are derived from [32]. The third last column enlists CNAs present in the HRO cohort. The second last column notes CNA gene gains and the last gene losses. Table G shows abundance of HRO and Swiss CNAs in comparison to published mutations. Haddad & Margulis et al. [42] enlist the top significantly mutated genes from several NGS studies. Genes are enlisted incl. their corresponding relative frequencies (Column B-H). Next to these values, relative abundances of HRO and Swiss CNAs (data from Beleut et al. [74]) are shown. Percentages are given as relative numbers of patients regarding all ccRCC patients or being classified by specific Fuhrman gradings.
(XLSX)

S16 File HRO and Swiss survival data in respect to assigned patient groups. Table A presents survival-data and gene counts of both HRO and Swiss data sets including assignments of patient groups based on gene set HRO201. Fig B (upper panel) visualizes survival data of Swiss patients stratified by three CNA patient groups is based on gene set HRO201 leading to classifications depicted in A. Regarding the Swiss data set, hazard ratio concerning both Swiss groups (A, Z vs. B-F) is 2.47 with 95% confidence interval at [0.69, 8.80] and a logrank test p-value of 0.151. The lower panel of Fig B is based on survival data categorized by Fuhrman grades G1, G2, G3 and G4 (number of patients are in parenthesis). Data are enlisted in A. The hazard ratio for G1+G2 vs. G3+G4 is 1.06 with 95% confidence interval at [0.29, 3.80] and a longrank-test p-value of 0.932. The hazard ratio for G2 vs. G3 is 0.65 with 95% confidence interval at [0.16, 2.66] and a longrank-test p-value of 0.551.
(XLSX)

S1 Table CNA gains of tumour suppressor genes regarding 9 G3 tumours. Detailed data regarding 10 tumour suppressor genes that show at least two gains in at least 2 G3-HRO tumours. “-1” describes losses, “1” gains. In column C, number of pathways of CNA genes under study.
(XLSX)

S1 Text CNAs common ccRCC tumours. CNA genes are described in Tables N and O in S2 File. CNA genes enlisted do not discriminate between Fuhrman G1 and G3 ccRCC tumours by Fisher’s exact testing.
(DOCX)

S2 Text ccRCC tumour stratification by CNA gene set HRO286. An extending detailed discussion of CNA gene set HRO 286 has been attached.
(DOCX)

S1 Fig Correlation heatmap HRO201 gene set. This heatmap generated by unsupervised hierarchical clustering (average linkage and Euclidian distance) represents a correlation-matrix of gene set HRO201 showing the correlation between ccRCC tumours (Table M in S2 File, Table B S14 File). Additionally, a second heatmap displays a subset of HRO and Swiss tumour samples (see colouring of patient IDs in Table B in S14).
(TIFF)
