# Supplementary figures and images for: Stratification of clear cell renal cell carcinoma (ccRCC) genomes by gene-directed copy number alteration (CNA) analysis
Source: PLoS One. 2017 May 9;12(5):e0176659. doi: 10.1371/journal.pone.0176659 (PMC5423597; doi:10.1371/journal.pone.0176659)

3p21.31

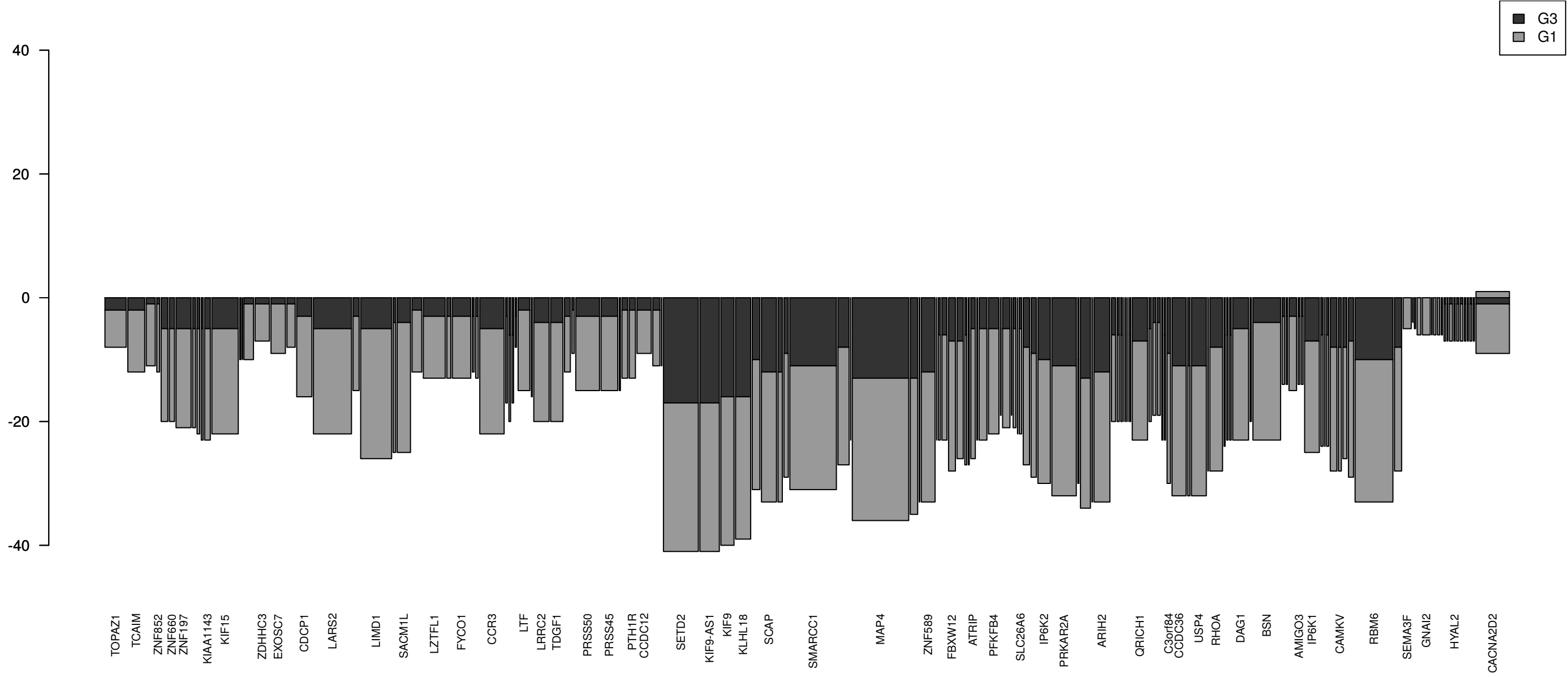

# 16p13.3

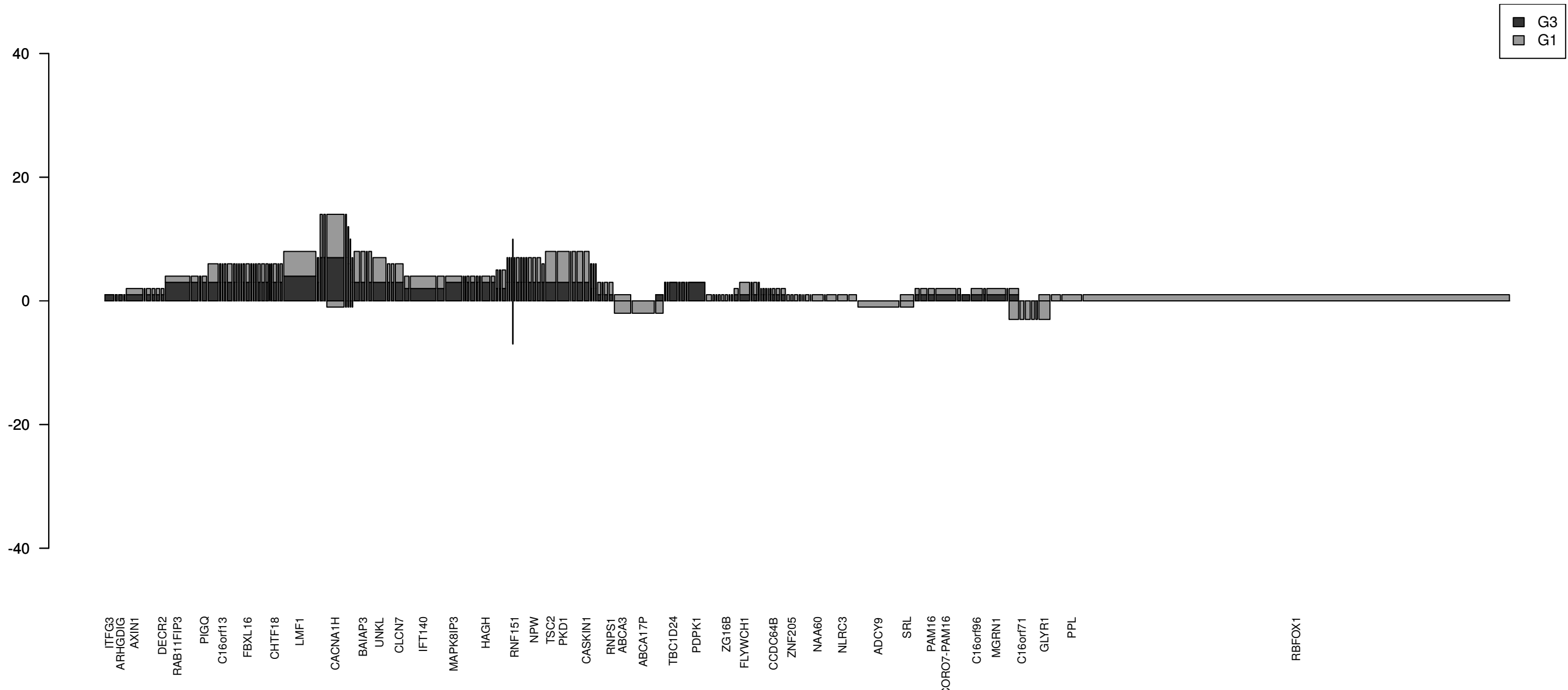

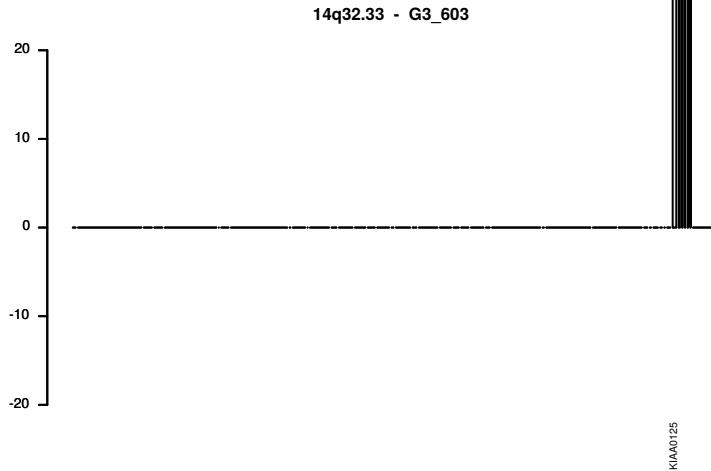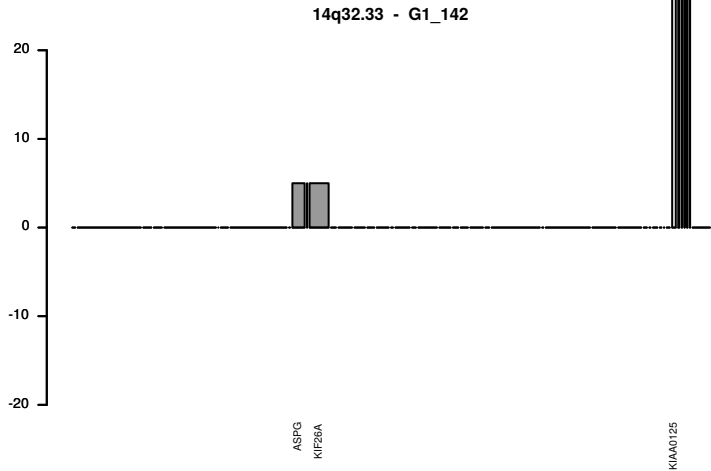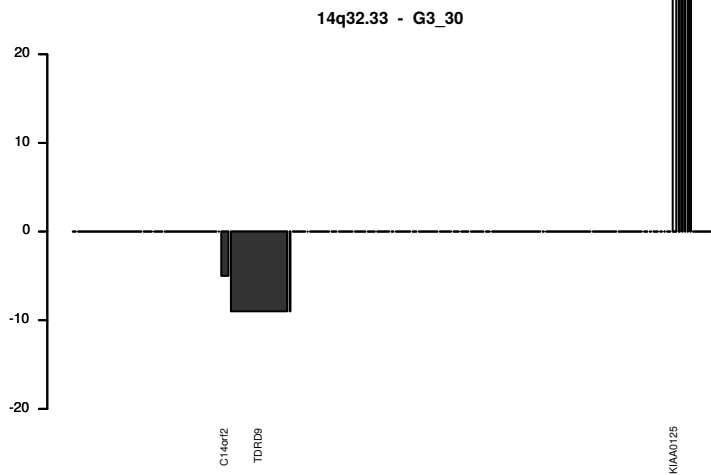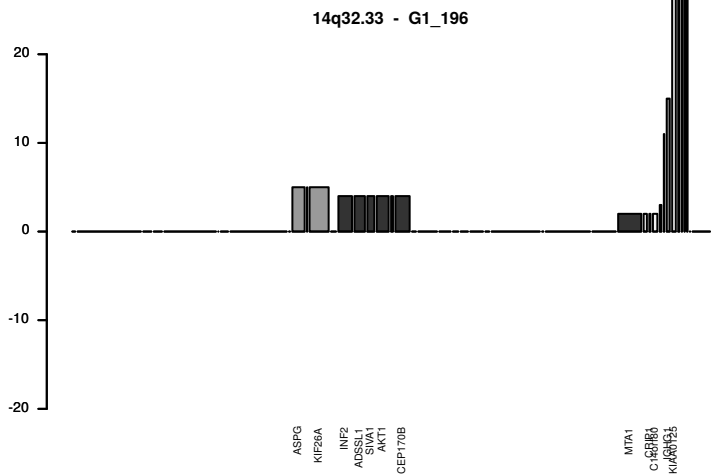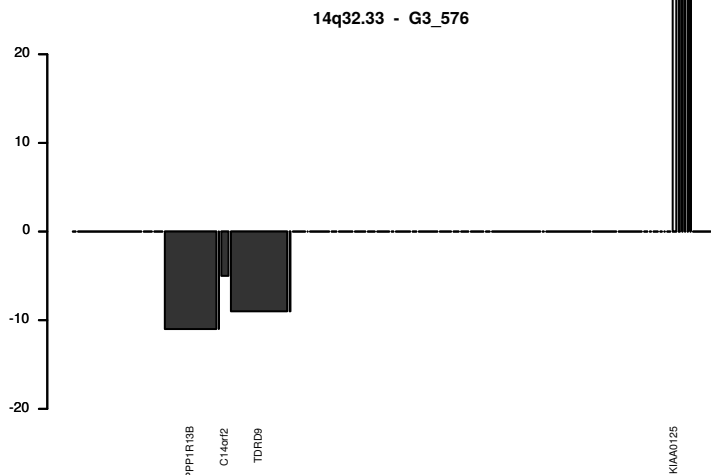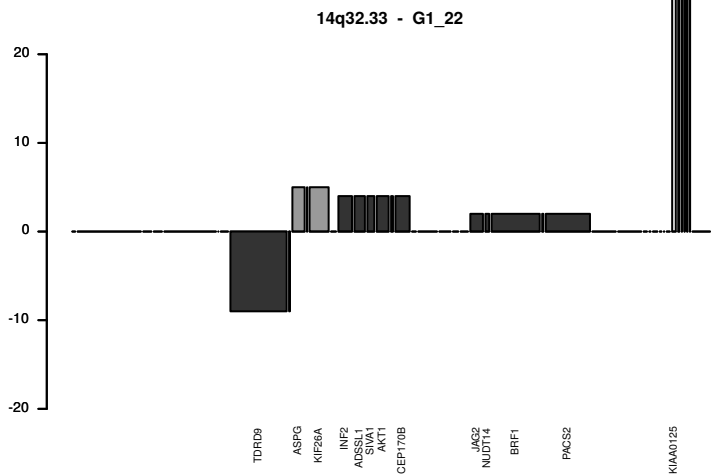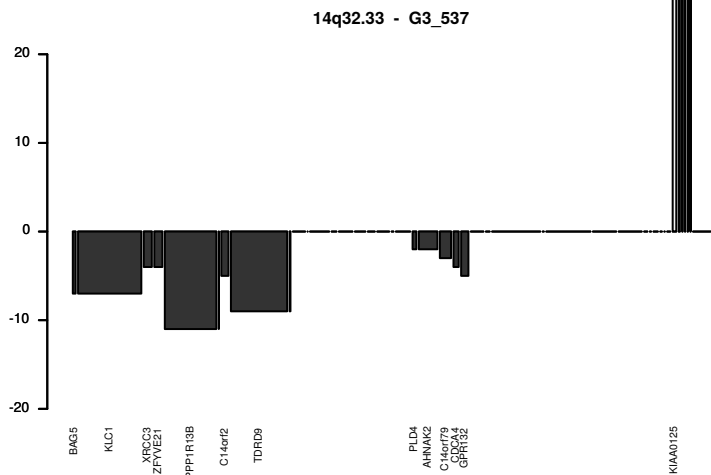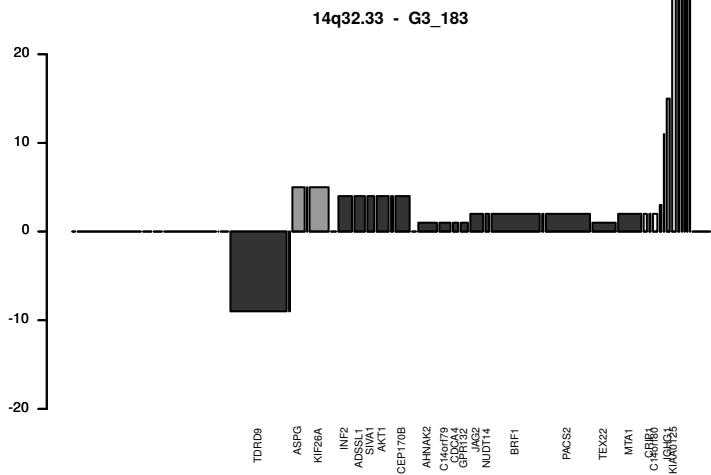

14q32.33

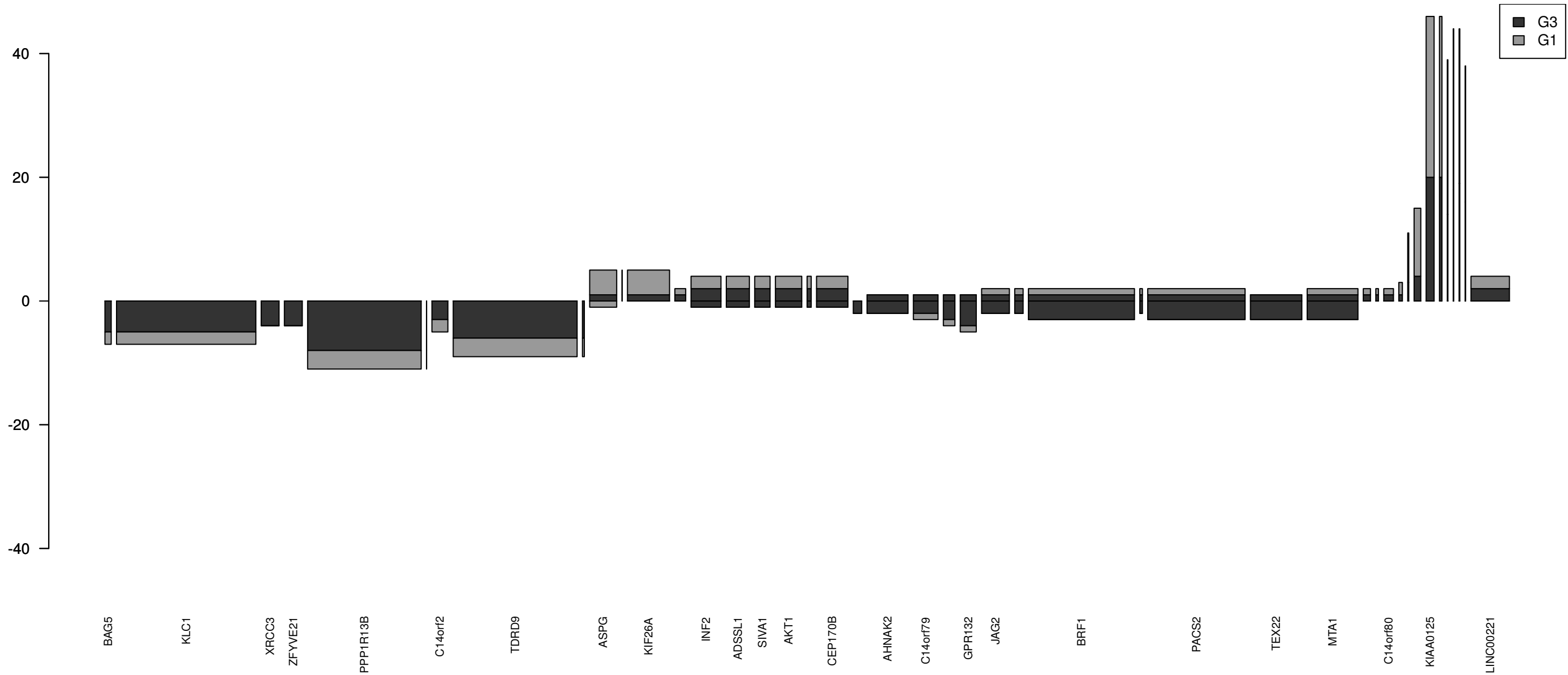

# 7q22.1

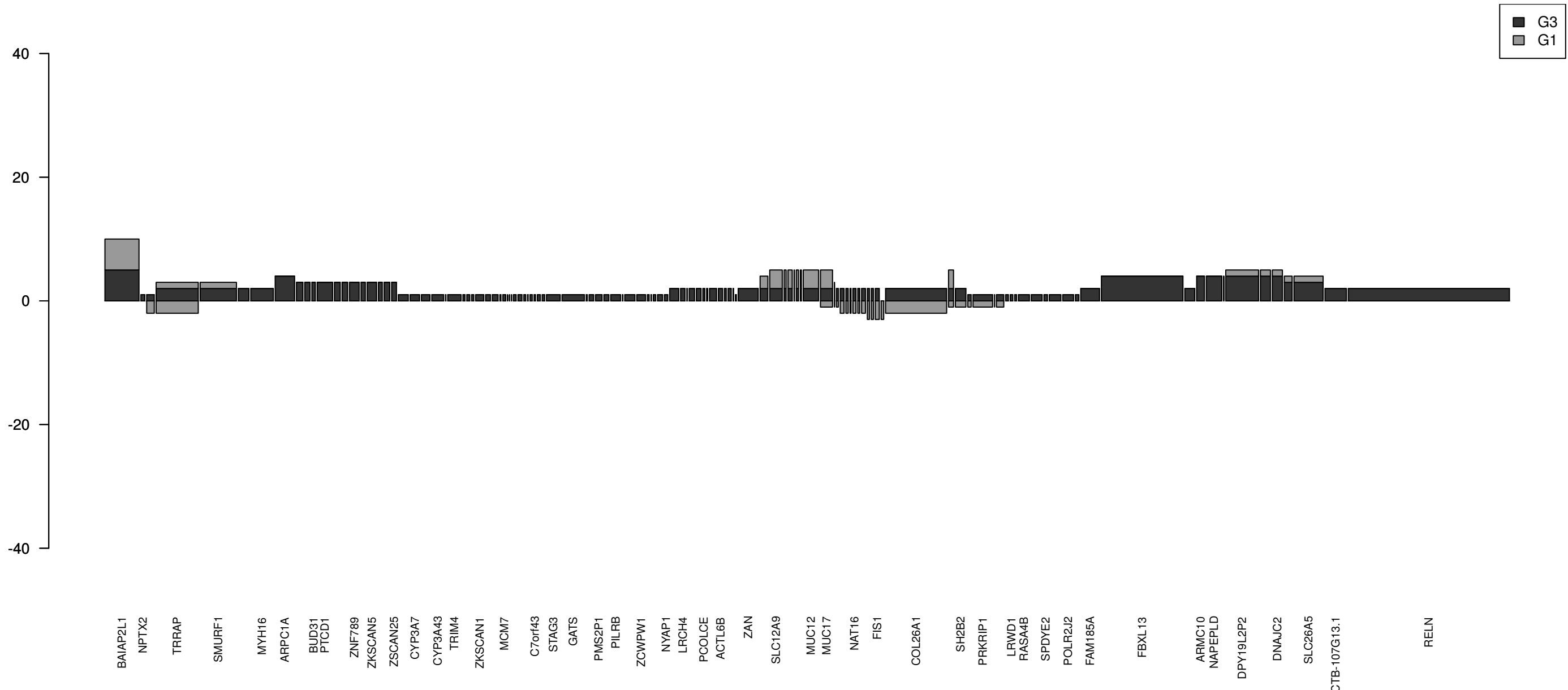

# 16p11.2

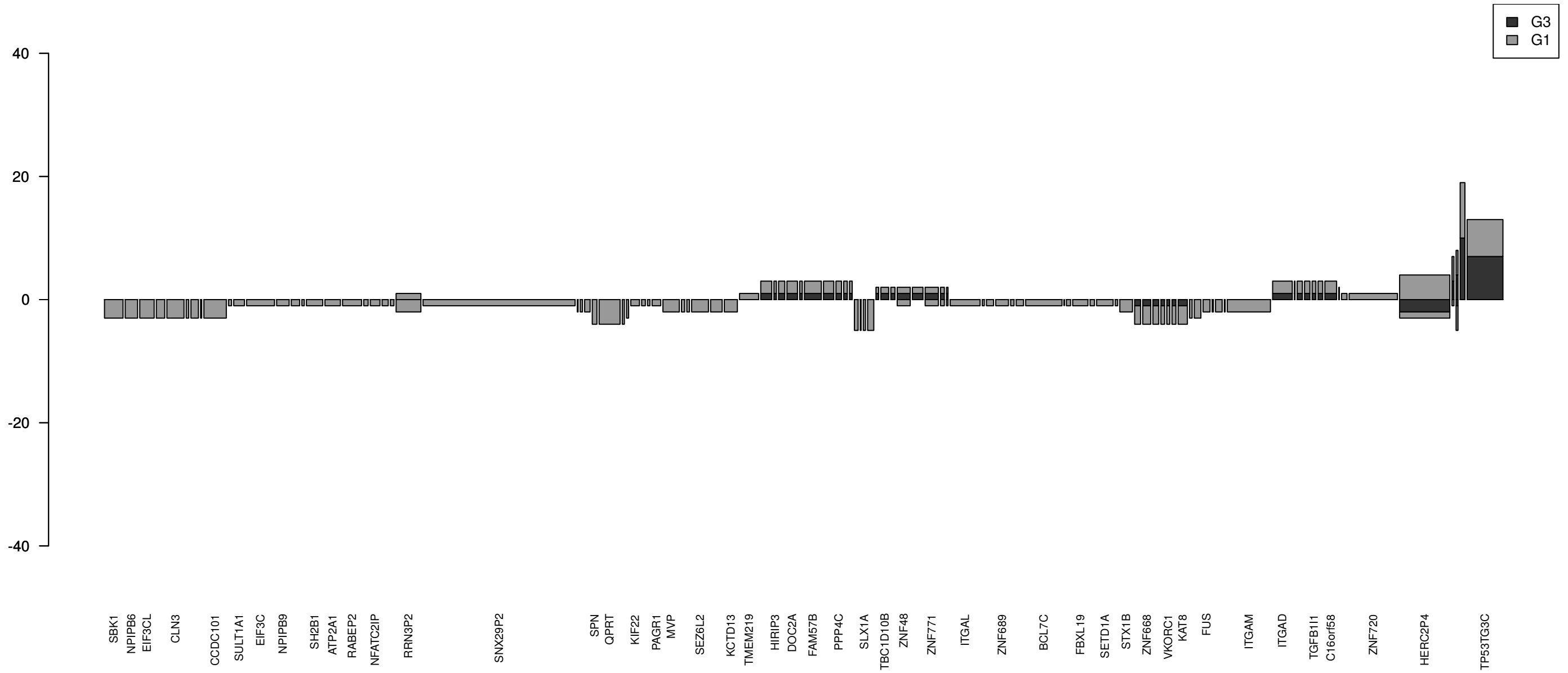

4p16.3 - G1\_286

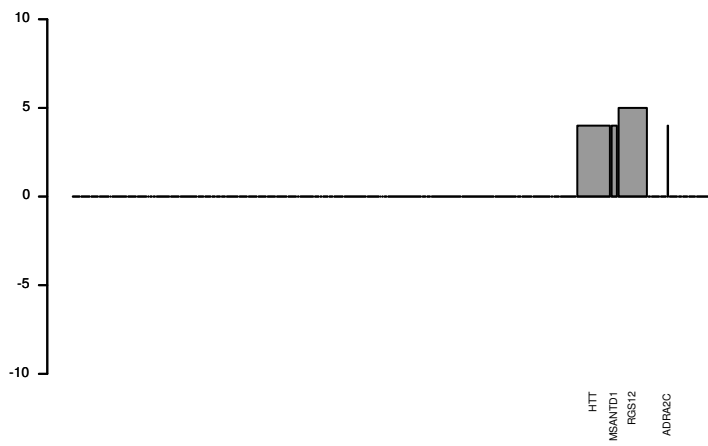

4p16.3 - G3\_526

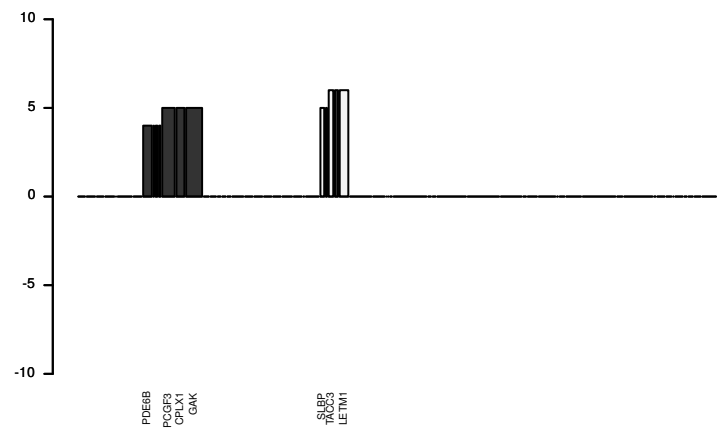

4p16.3 - G1\_294

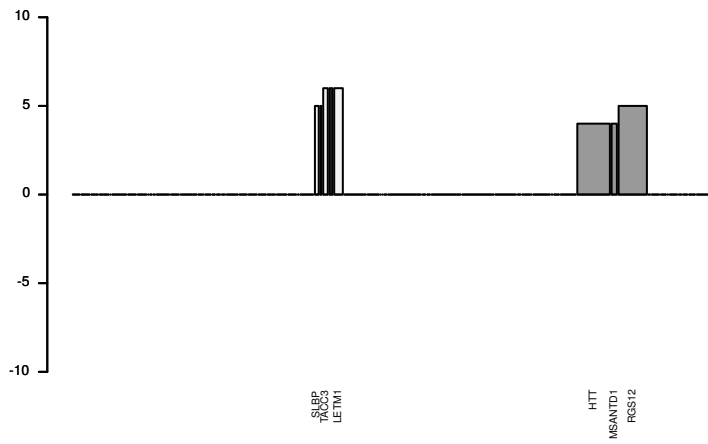

4p16.3 - G3\_30

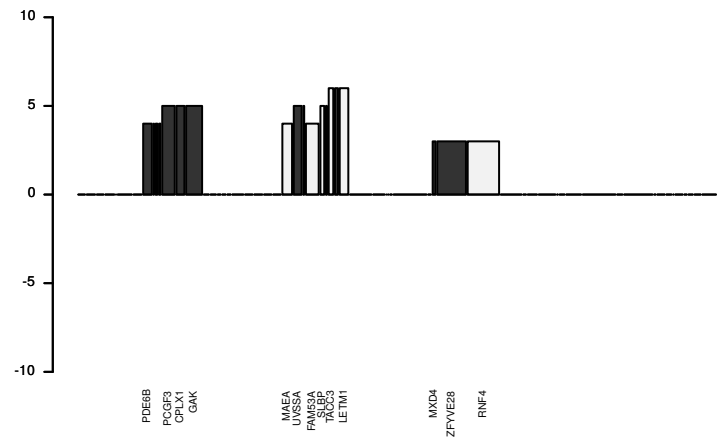

4p16.3 - G1\_290

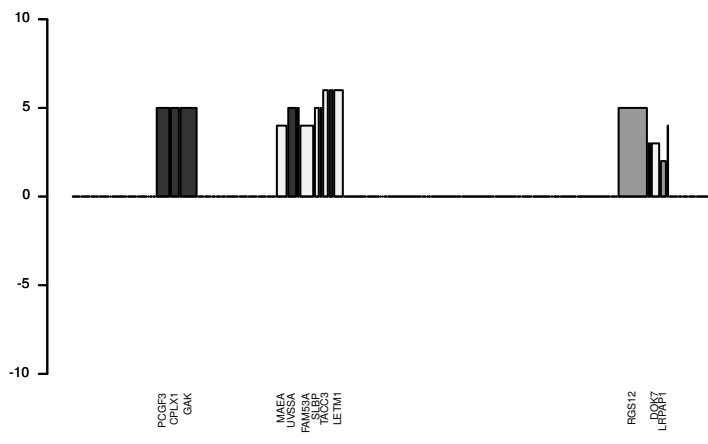

4p16.3 - G3\_531

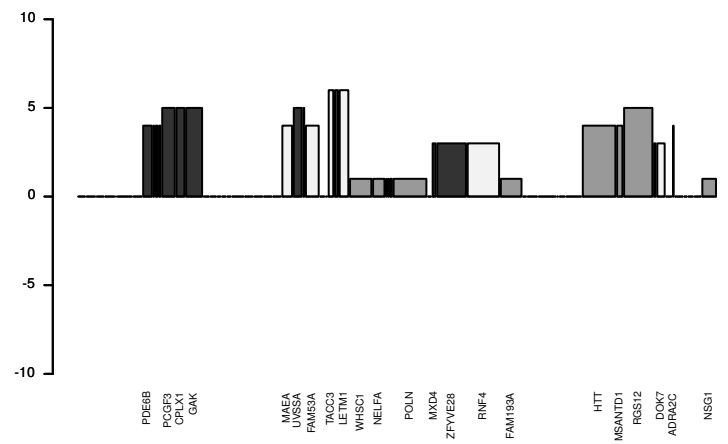

4p16.3 - G3\_183

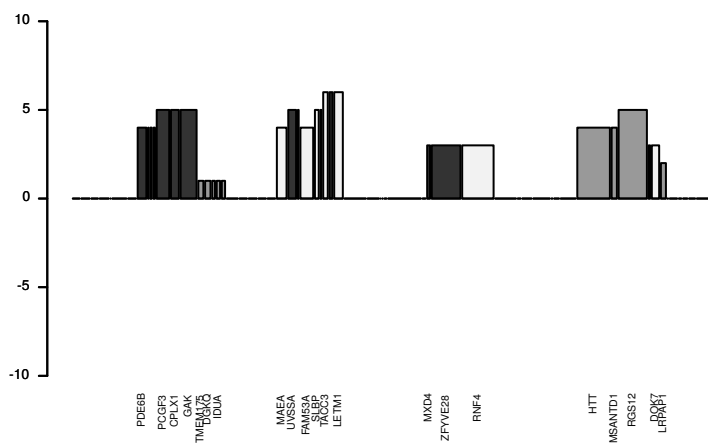

Supplement: S4 File — CNA gene assignments to chromosomal loci visualizing Fuhrman grades G1 and G3.A: Cytoband-plot of 3p21.31 is shown for all HRO tumours. Bars consist of up to 2 colours, representing the number of tumours assigned by Fuhrman grades G1 and G3: medium gray represents G1 and dark gray G3. B: Cytoband-plot of 16p13.3 representing all HRO tumours. C: Cytoband-plot of 14q32.33 representing 8 different HRO tumours. The height of bars documents number of tumours that share CNAs. Assignments of colourings are dark = G3 nominator genes, medium = G1 nominator genes, light = genes without any preference for Fuhrman malignancy grades. D: Cytoband-plot of 14q32.33 representing all HRO tumours. E: Cytoband-plot of 7q22.1 representing all HRO tumours. F: A cytoband-plot (like A) representing locus 16p11.2 G: Cytoband-plot of locus 4p16.3 showing only CNAs of 7 selected HRO tumours. (PDF) [file pone.0176659.s004.pdf]

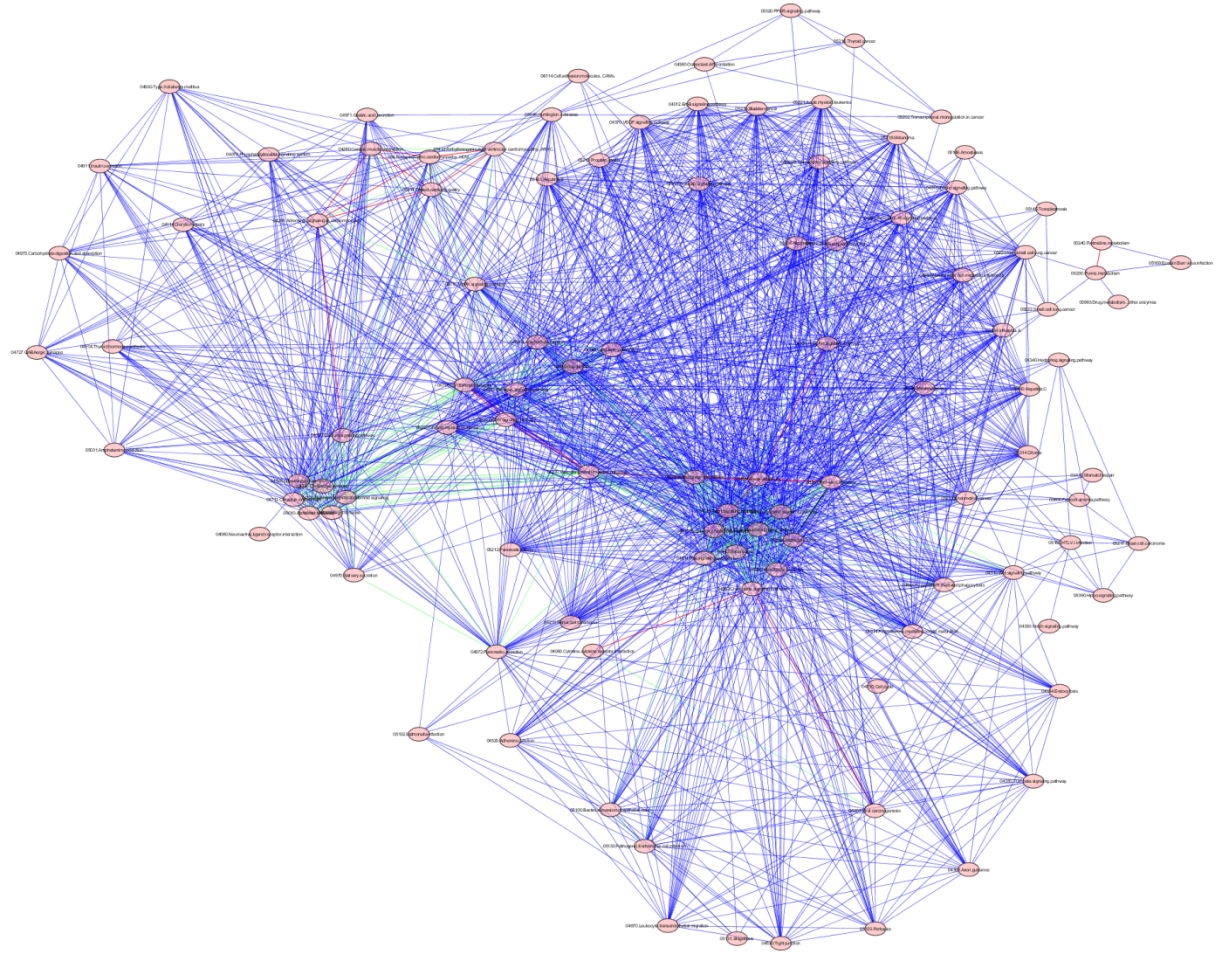

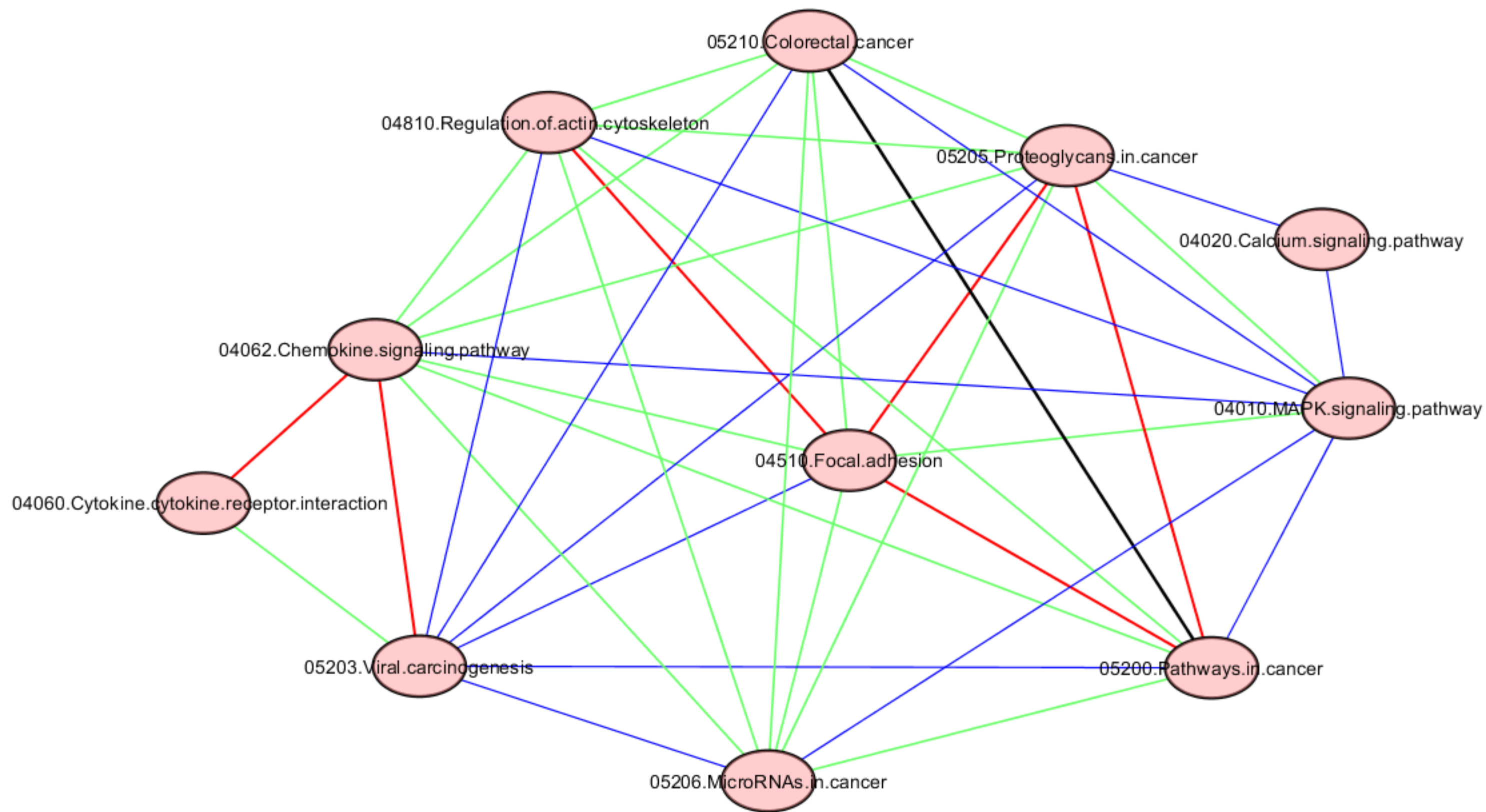

Supplement: S10 File — A: All pathways of assigned CNAs present in at least 20 HRO tumours are visualized by Cytoscape [82], see data Table A in S11 File. B: Most common 11 pathways of assigned CNAs present in at least 20 HRO tumours are visualized by Cytoscape [82], see data Table B in S11 File. (PDF) [file pone.0176659.s010.pdf]

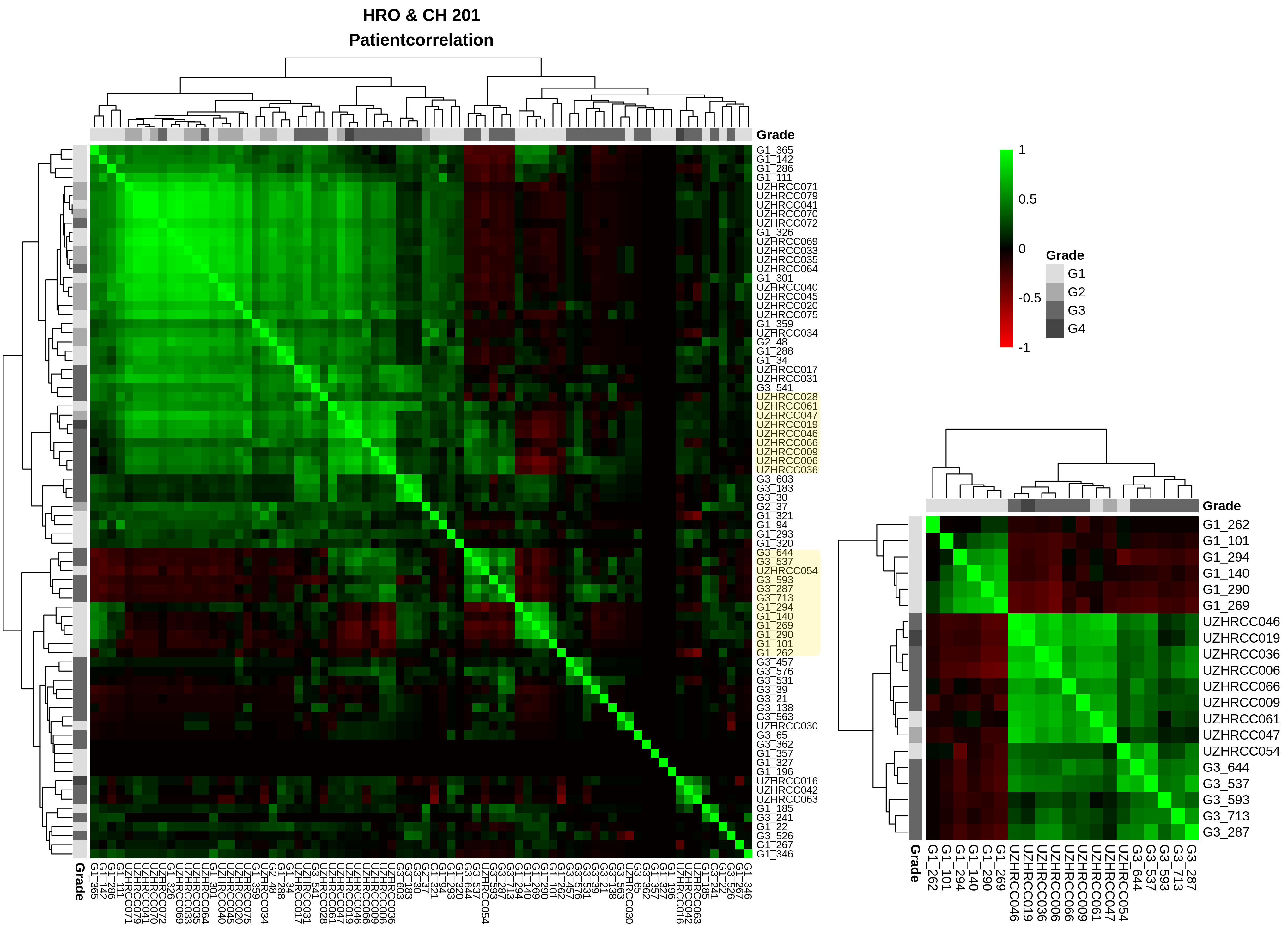

Supplement: S1 Fig — This heatmap generated by unsupervised hierarchical clustering (average linkage and Euclidian distance) represents a correlation-matrix of gene set HRO201 showing the correlation between ccRCC tumours (Table M in S2 File, Table B S14 File). Additionally, a second heatmap displays a subset of HRO and Swiss tumour samples (see colouring of patient IDs in Table B in S14). (TIFF) [file pone.0176659.s020.tiff]
