# Supplementary material for: Stratification of clear cell renal cell carcinoma (ccRCC) genomes by gene-directed copy number alteration (CNA) analysis
Source: PLoS One. 2017 May 9;12(5):e0176659. doi: 10.1371/journal.pone.0176659 (PMC5423597; doi:10.1371/journal.pone.0176659)
Supplement: S6 File — Unsupervised hierarchical clustering by average linkage and Euclidian distance, representing a correlation-matrix, show Pearson correlation between tumours, based on: A: CNA losses per cytoband; B: CNA gains per cytoband; C: CNA losses per cytoband but restricted to highly altered cytobands affected by at least 20 tumours; D: CNA gains per cytoband but restricted to highly altered cytobands affected by at least 20 tumours. E: CNA losses per cytoband but limited to specific cytobands (p-value below 10−14). Colour-code follows as: High positive correlation is represented by green and high negative correlation by red. (PDF) [file pone.0176659.s006.pdf]

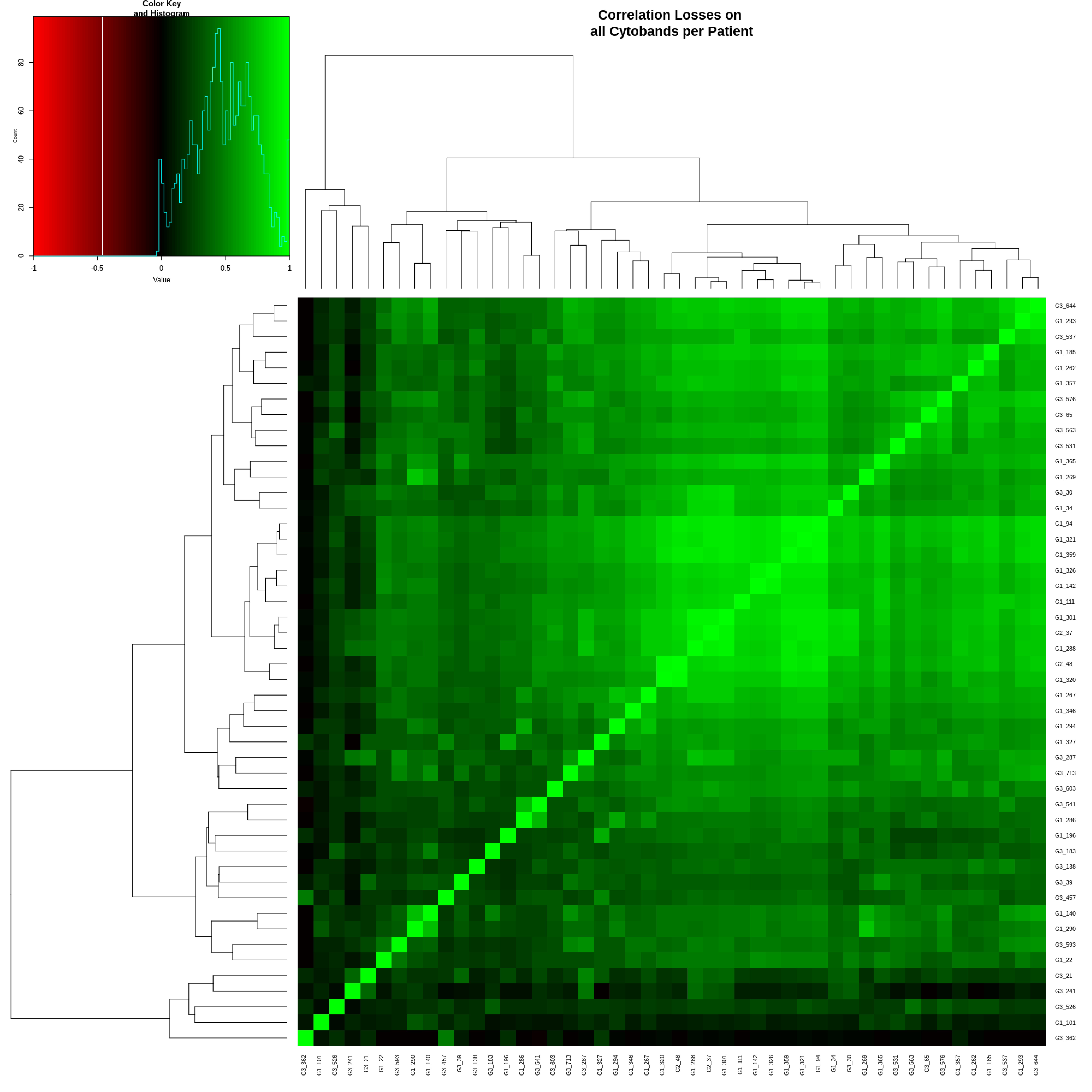
Color Key and Histogram
Count
Value
Correlation Losses on all Cytobands per Patient
G3_362 G1_101 G3_526 G3_241 G3_21 G1_22 G3_593 G1_290 G1_140 G3_457 G3_39 G3_138 G3_183 G1_196 G1_286 G3_541 G3_603 G3_713 G3_287 G1_327 G1_294 G1_346 G1_267 G1_320 G2_48 G1_288 G2_37 G1_301 G1_111 G1_142 G1_326 G1_359 G1_321 G1_94 G1_34 G3_30 G1_269 G1_365 G3_531 G3_563 G3_65 G3_576 G1_357 G1_262 G1_185 G3_537 G1_293 G3_644

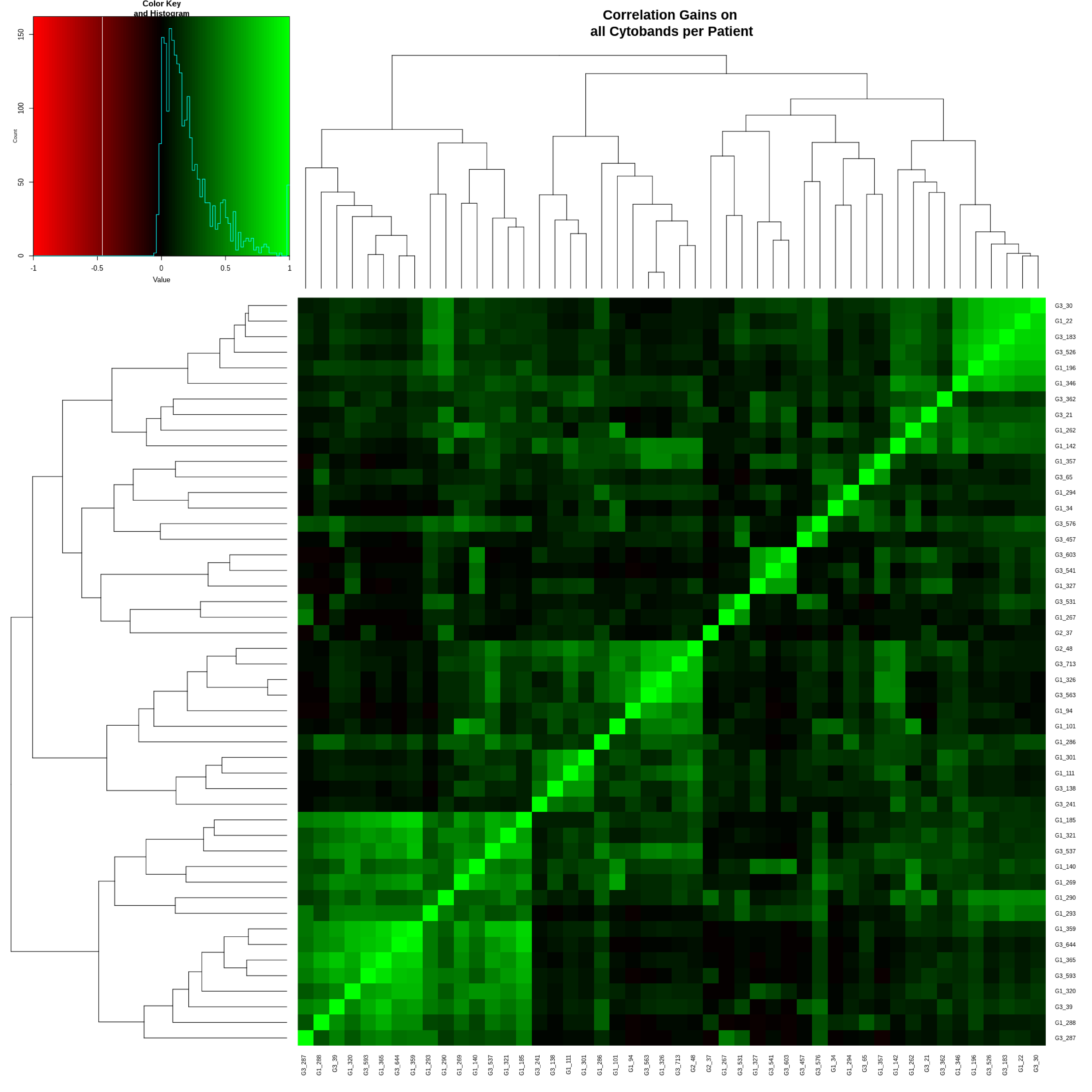
Color Key
and Histogram
Count
150
100
50
0
-1
-0.5
0
0.5
1
Value
Correlation Gains on
all Cytobands per Patient
G3_30
G1_22
G3_183
G3_526
G1_196
G1_346
G3_362
G3_21
G1_262
G1_142
G1_357
G3_65
G1_294
G1_34
G3_576
G3_457
G3_603
G3_541
G1_327
G3_531
G1_267
G2_37
G2_48
G3_713
G1_326
G3_563
G1_94
G1_101
G1_286
G1_301
G1_111
G3_138
G3_241
G1_185
G1_321
G3_537
G1_140
G1_269
G1_290
G1_293
G1_359
G3_644
G1_365
G3_593
G1_320
G3_39
G1_288
G3_287

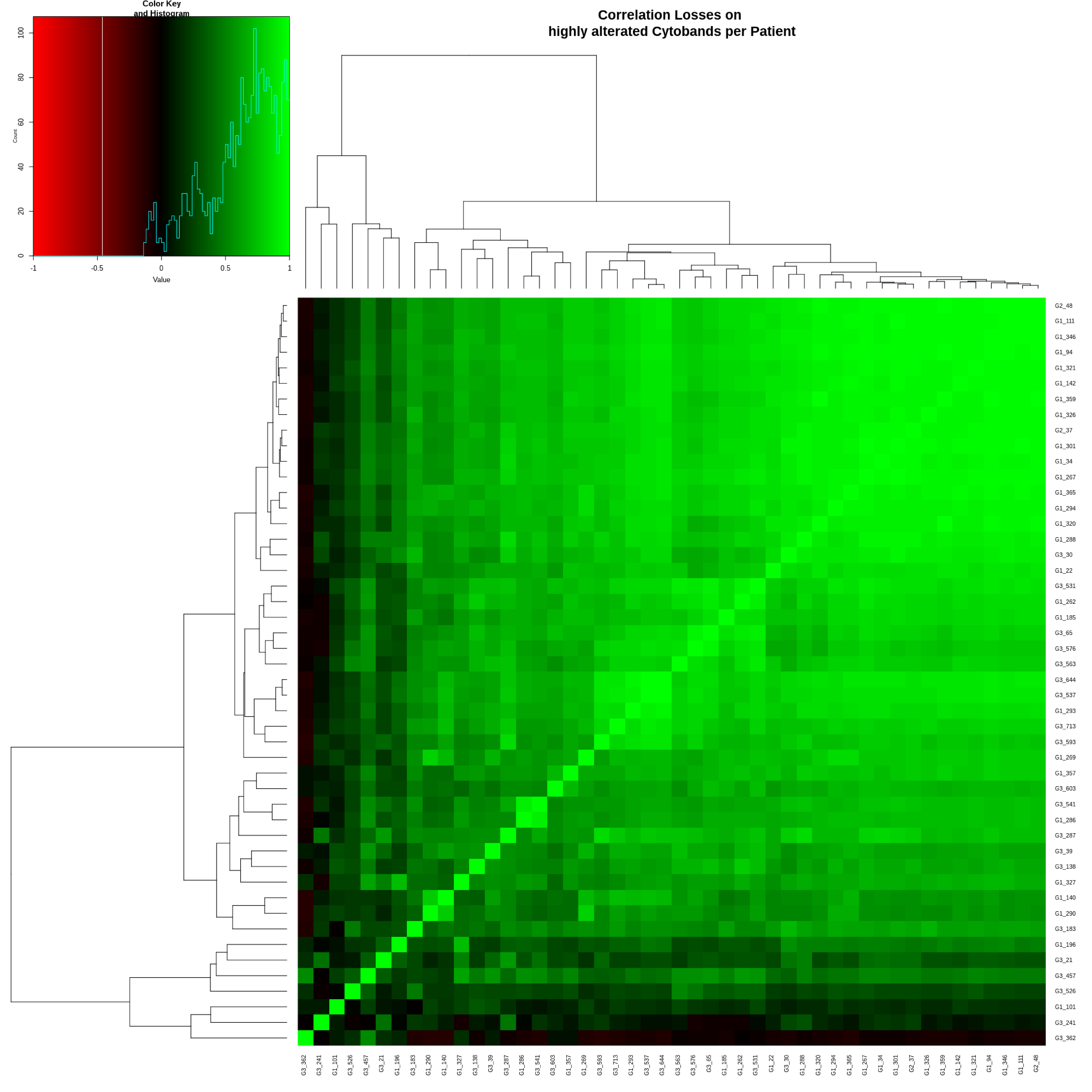
Color Key
and Histogram
Count
0
20
40
60
80
100
-1
-0.5
0
0.5
1
Value
Correlation Losses on
highly alterated Cytobands per Patient
G2_48
G1_111
G1_346
G1_94
G1_321
G1_142
G1_359
G1_326
G2_37
G1_301
G1_34
G1_267
G1_365
G1_294
G1_320
G1_288
G3_30
G1_22
G3_531
G1_262
G1_185
G3_65
G3_576
G3_563
G3_644
G3_537
G1_293
G3_713
G3_593
G1_269
G1_357
G3_603
G3_541
G1_286
G3_287
G3_39
G3_138
G1_327
G1_140
G1_290
G3_183
G1_196
G3_21
G3_457
G3_526
G1_101
G3_241
G3_362
G3_362
G3_241
G1_101
G3_526
G3_457
G3_21
G1_196
G3_183
G1_290
G1_140
G1_327
G3_138
G3_39
G3_287
G1_286
G3_541
G3_603
G1_357
G1_269
G3_593
G3_713
G1_293
G3_537
G3_644
G3_563
G3_576
G3_65
G1_185
G1_262
G3_531
G1_22
G3_30
G1_288
G1_320
G1_294
G1_365
G1_267
G1_34
G1_301
G2_37
G1_326
G1_359
G1_142
G1_321
G1_94
G1_346
G1_111
G2_48

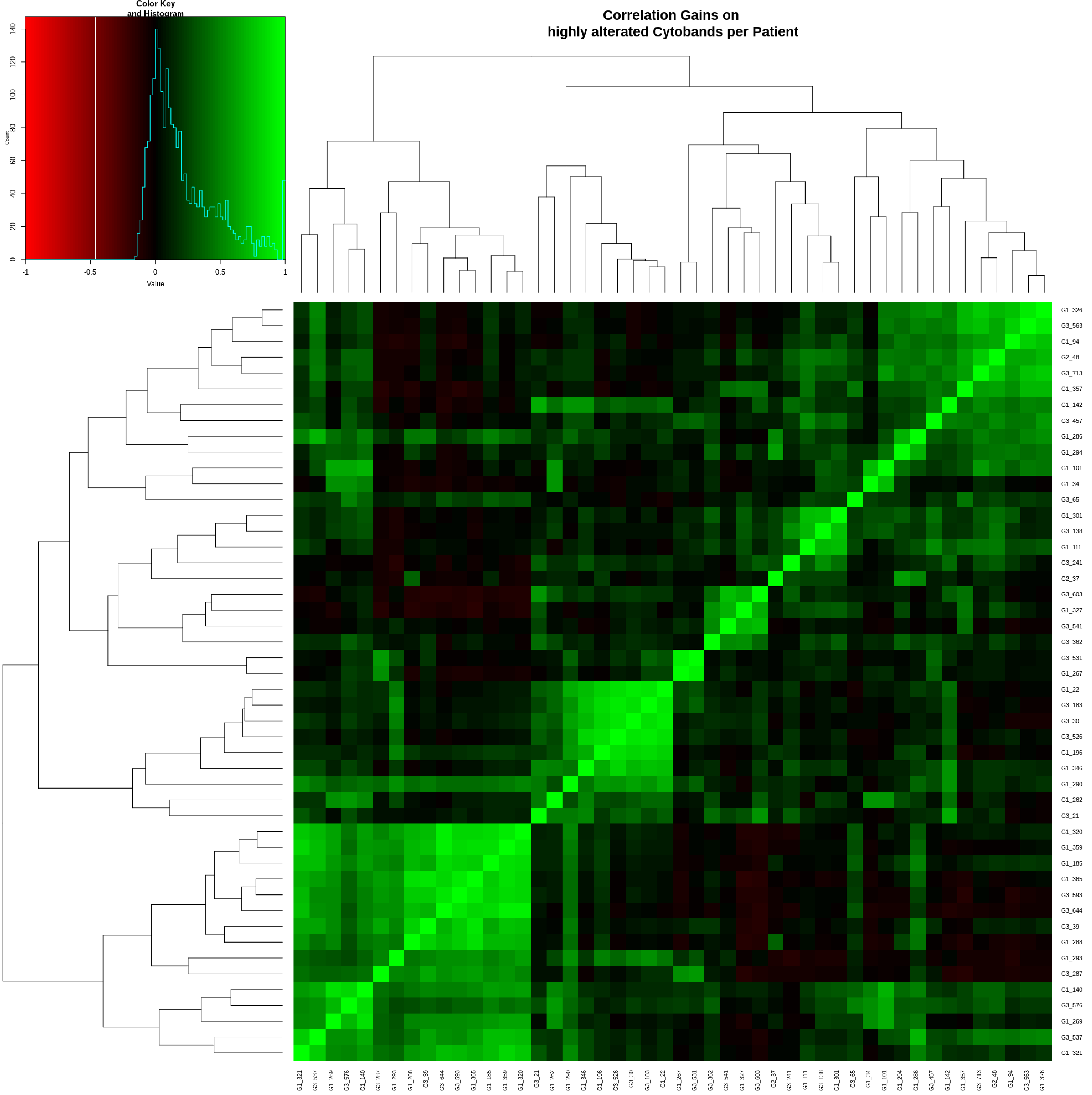

Color Key
and Histogram
Count
Value
Correlation Gains on
highly alterated Cytobands per Patient
G1_326
G3_563
G1_94
G2_48
G3_713
G1_357
G1_142
G3_457
G1_286
G1_294
G1_101
G1_34
G3_65
G1_301
G3_138
G1_111
G3_241
G2_37
G3_603
G1_327
G3_541
G3_362
G3_531
G1_267
G1_22
G3_183
G3_30
G3_526
G1_196
G1_346
G1_290
G1_262
G3_21
G1_320
G1_359
G1_185
G1_365
G3_593
G3_644
G3_39
G1_288
G1_293
G3_287
G1_140
G3_576
G1_269
G3_537
G1_321

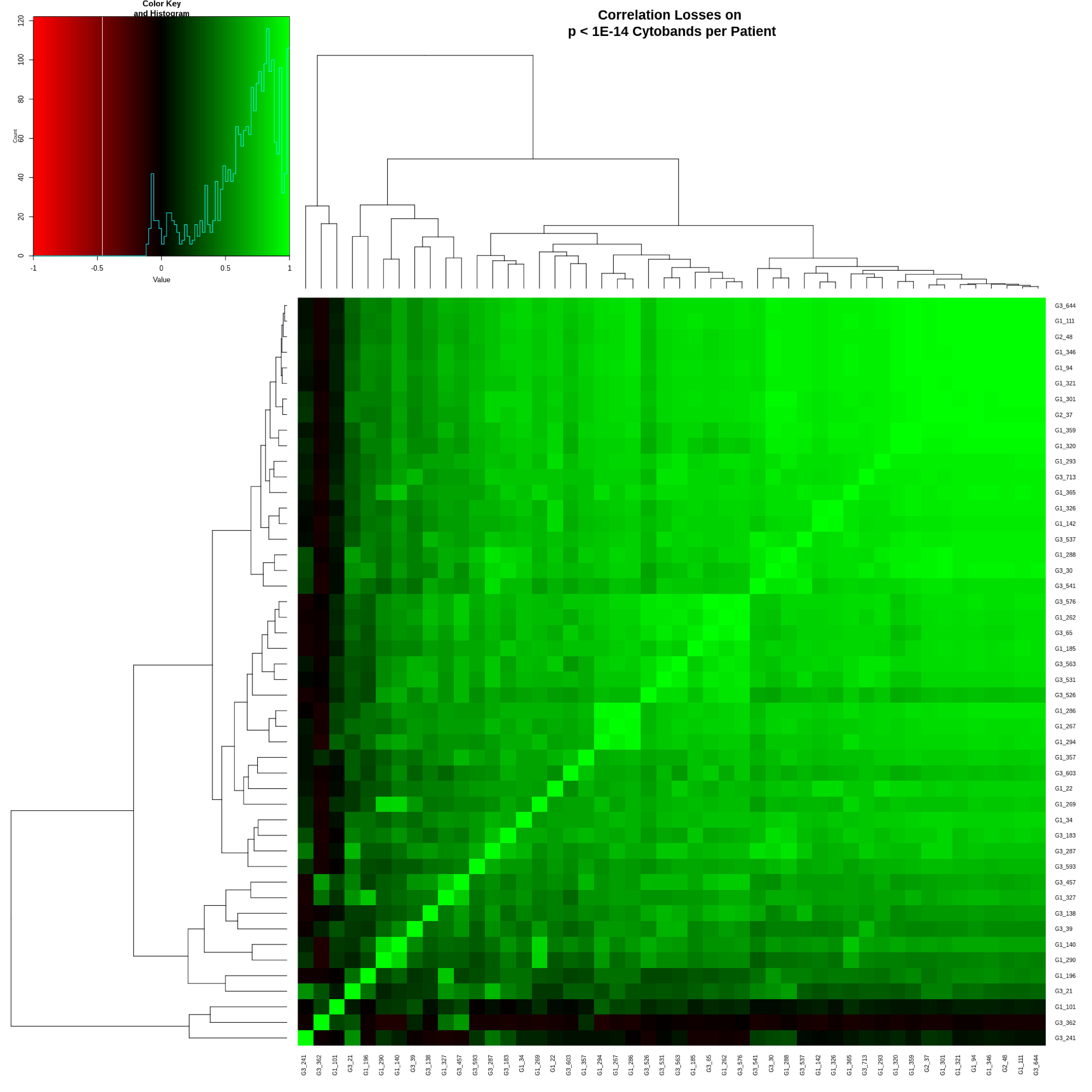
Color Key
and Histogram
Count
0
20
40
60
80
100
120
-1
-0.5
0
0.5
1
Value
Correlation Losses on
p < 1E-14 Cytobands per Patient
G3_644
G1_111
G2_48
G1_346
G1_94
G1_321
G1_301
G2_37
G1_359
G1_320
G1_293
G3_713
G1_365
G1_326
G1_142
G3_537
G1_288
G3_30
G3_541
G3_576
G1_262
G3_65
G1_185
G3_563
G3_531
G3_526
G1_286
G1_267
G1_294
G1_357
G3_603
G1_22
G1_269
G1_34
G3_183
G3_287
G3_593
G3_457
G1_327
G3_138
G3_39
G1_140
G1_290
G1_196
G3_21
G1_101
G3_362
G3_241
G3_241
G3_362
G1_101
G3_21
G1_196
G1_290
G1_140
G3_39
G3_138
G1_327
G3_457
G3_593
G3_287
G3_183
G1_34
G1_269
G1_22
G3_603
G1_357
G1_294
G1_267
G1_286
G3_526
G3_531
G3_563
G1_185
G3_65
G1_262
G3_576
G3_541
G3_30
G1_288
G3_537
G1_142
G1_326
G1_365
G3_713
G1_293
G1_320
G1_359
G2_37
G1_301
G1_321
G1_94
G1_346
G2_48
G1_111
G3_644
