# Supplementary material for: Stratification of clear cell renal cell carcinoma (ccRCC) genomes by gene-directed copy number alteration (CNA) analysis
Source: PLoS One. 2017 May 9;12(5):e0176659. doi: 10.1371/journal.pone.0176659 (PMC5423597; doi:10.1371/journal.pone.0176659)
Supplement: S7 File — Unsupervised hierarchical clustering by average linkage and Euclidian distance, representing a correlation-matrix, show Pearson correlation of: A: losses between all cytobands; B: gains between all cytobands; C: losses between highly altered cytobands affected by at least 20 tumours; D: gains between highly altered cytobands affected by at least 20 tumours;E: gains between significant cytobands (p-value below 10−14). Colour-code follows as: High positive correlation is represented by green and high negative correlation by red. (PDF) [file pone.0176659.s007.pdf]

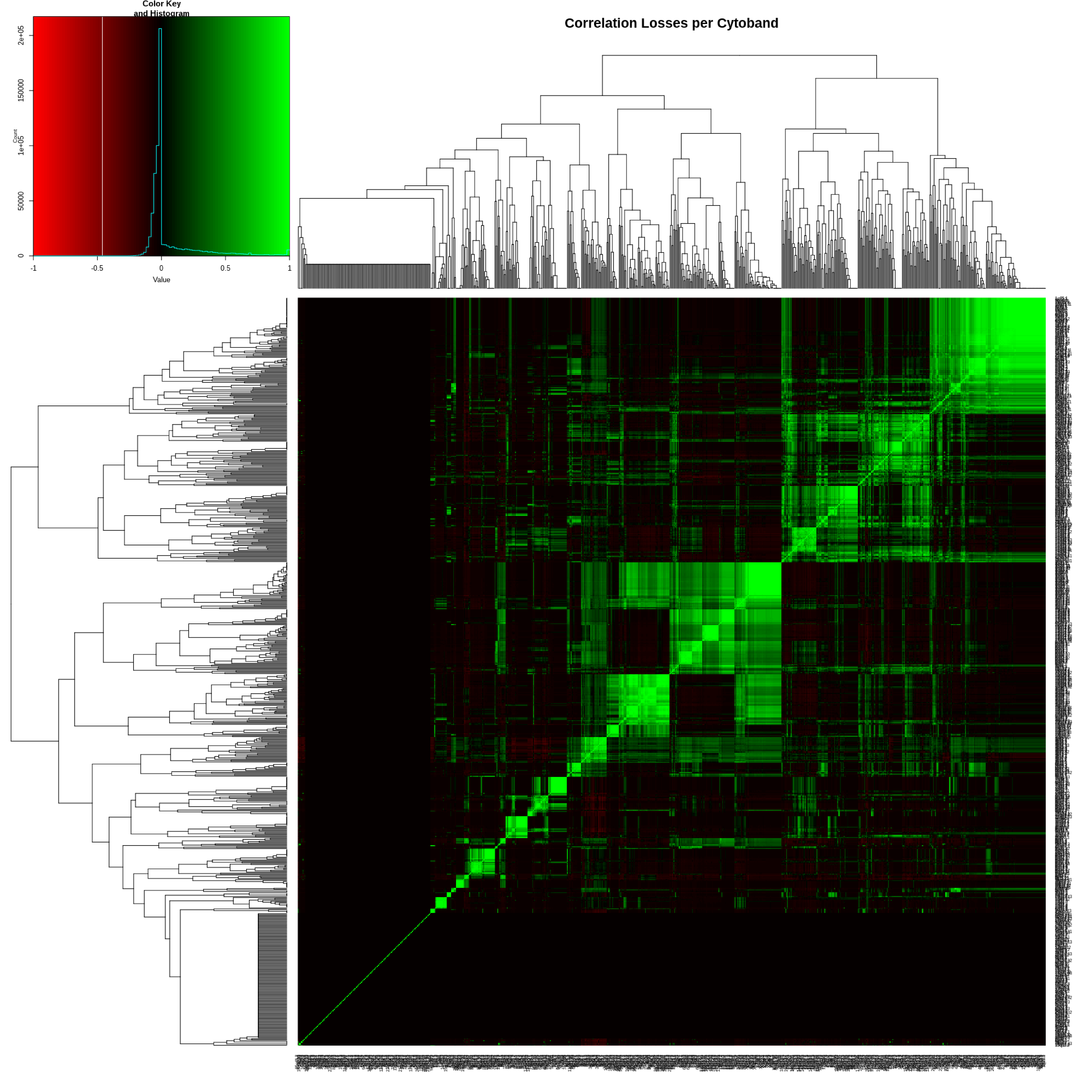
Color Key
and Histogram
Count
Value
-1
-0.5
0
0.5
1
0
50000
1e+05
150000
2e+05
Correlation Losses per Cytoband

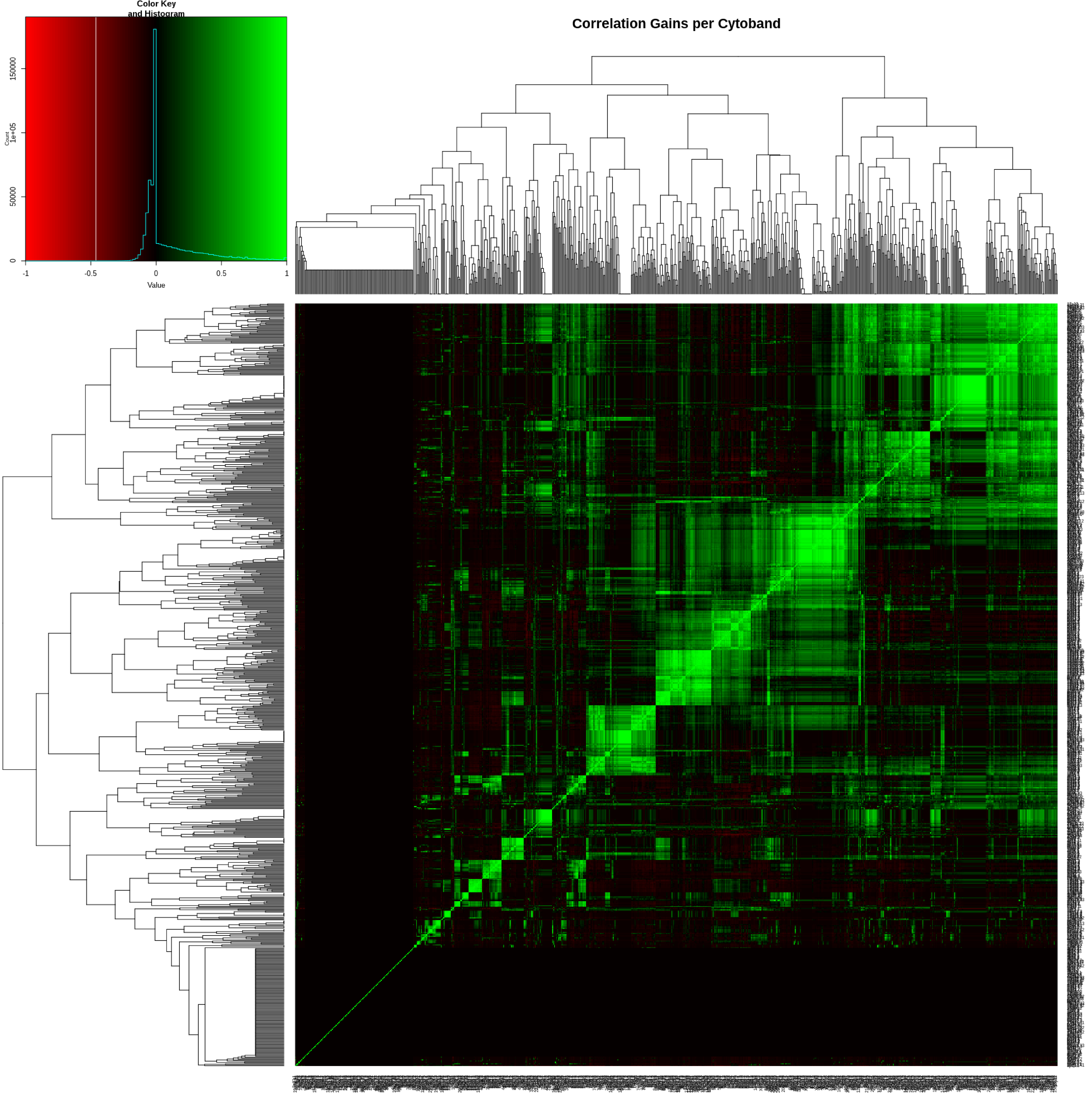

Color Key
and Histogram
Count
Value
Correlation Gains per Cytoband

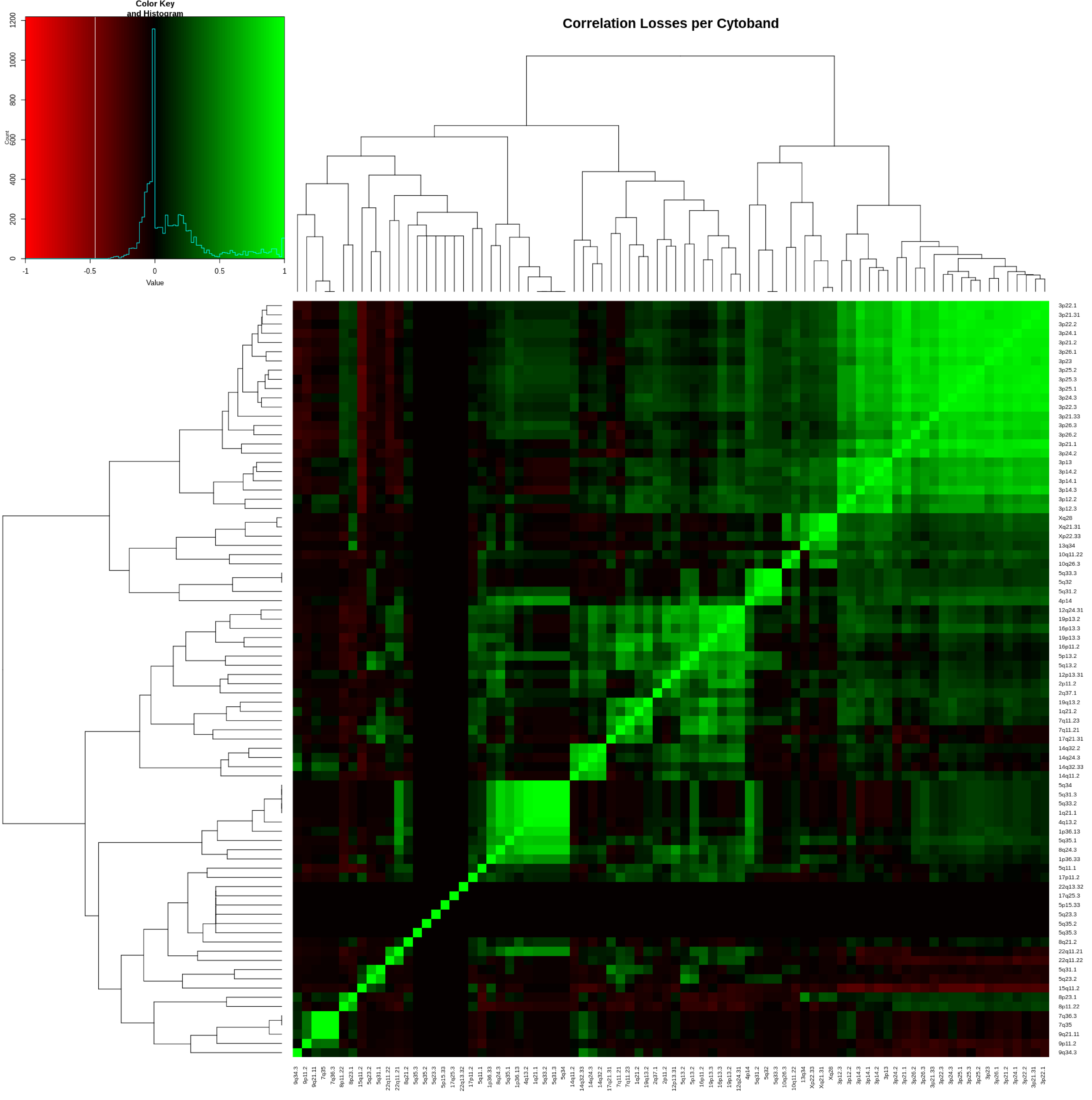

Color Key and Histogram
Count
Value
Correlation Losses per Cytoband

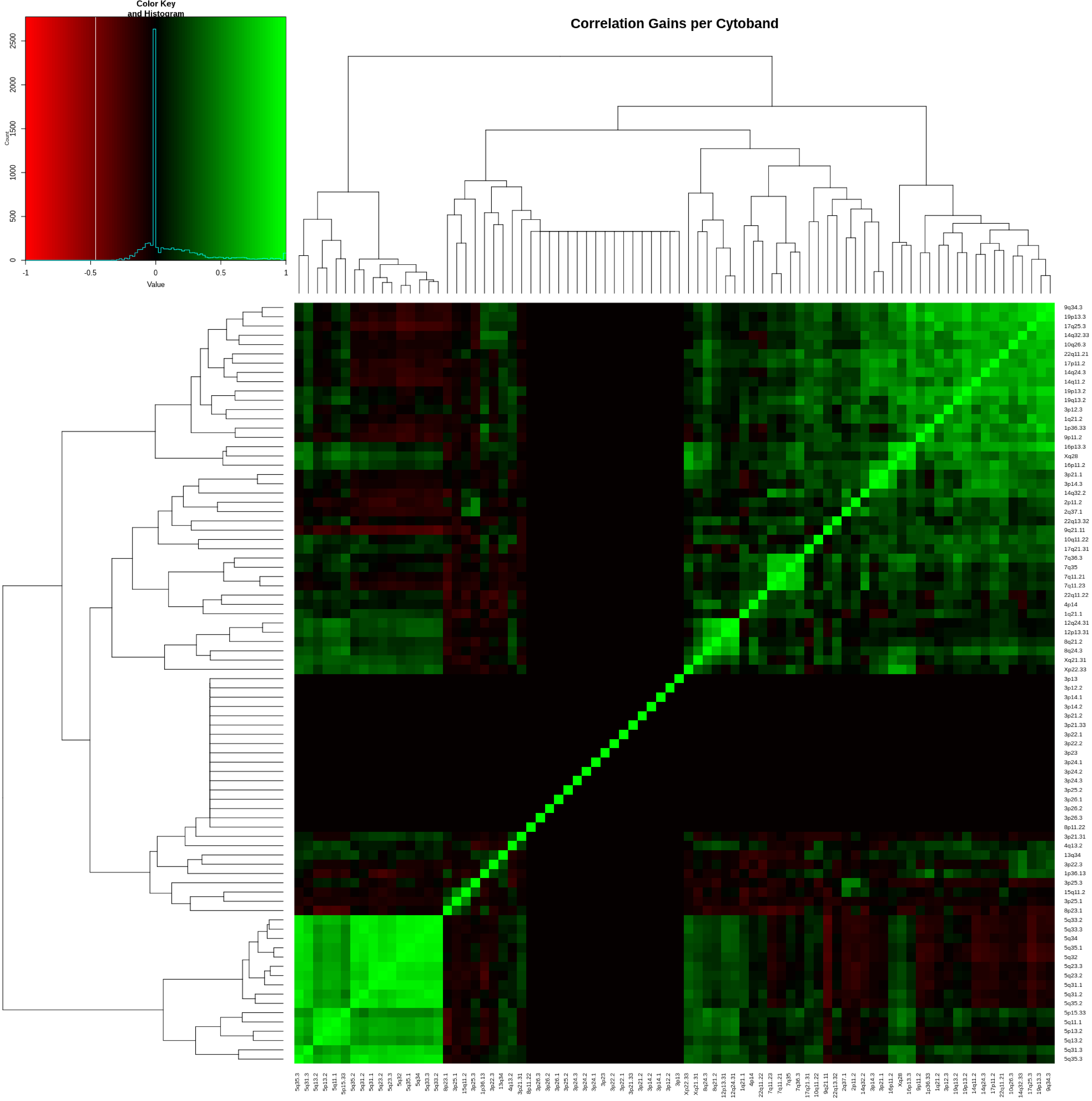
Correlation Gains per Cytoband
Color Key and Histogram
Count
2500
2000
1500
1000
500
0
-1
-0.5
0
0.5
1
Value
9q34.3
19p13.3
17q25.3
14q32.33
10q26.3
22q11.21
17p11.2
14q24.3
14q11.2
19p13.2
19q13.2
3p12.3
1q21.2
1p36.33
9p11.2
16p13.3
Xq28
16p11.2
3p21.1
3p14.3
14q32.2
2p11.2
2q37.1
22q13.32
9q21.11
10q11.22
17q21.31
7q36.3
7q35
7q11.21
7q11.23
22q11.22
4p14
1q21.1
12q24.31
12p13.31
8q21.2
8q24.3
Xq21.31
Xp22.33
3p13
3p12.2
3p14.1
3p14.2
3p21.2
3p21.33
3p22.1
3p22.2
3p23
3p24.1
3p24.2
3p24.3
3p25.2
3p26.1
3p26.2
3p26.3
8p11.22
3p21.31
4q13.2
13q34
3p22.3
1p36.13
3p25.3
15q11.2
3p25.1
8p23.1
5q33.2
5q33.3
5q34
5q35.1
5q32
5q23.3
5q23.2
5q31.1
5q31.2
5q35.2
5p15.33
5q11.1
5p13.2
5q13.2
5q31.3
5q35.3

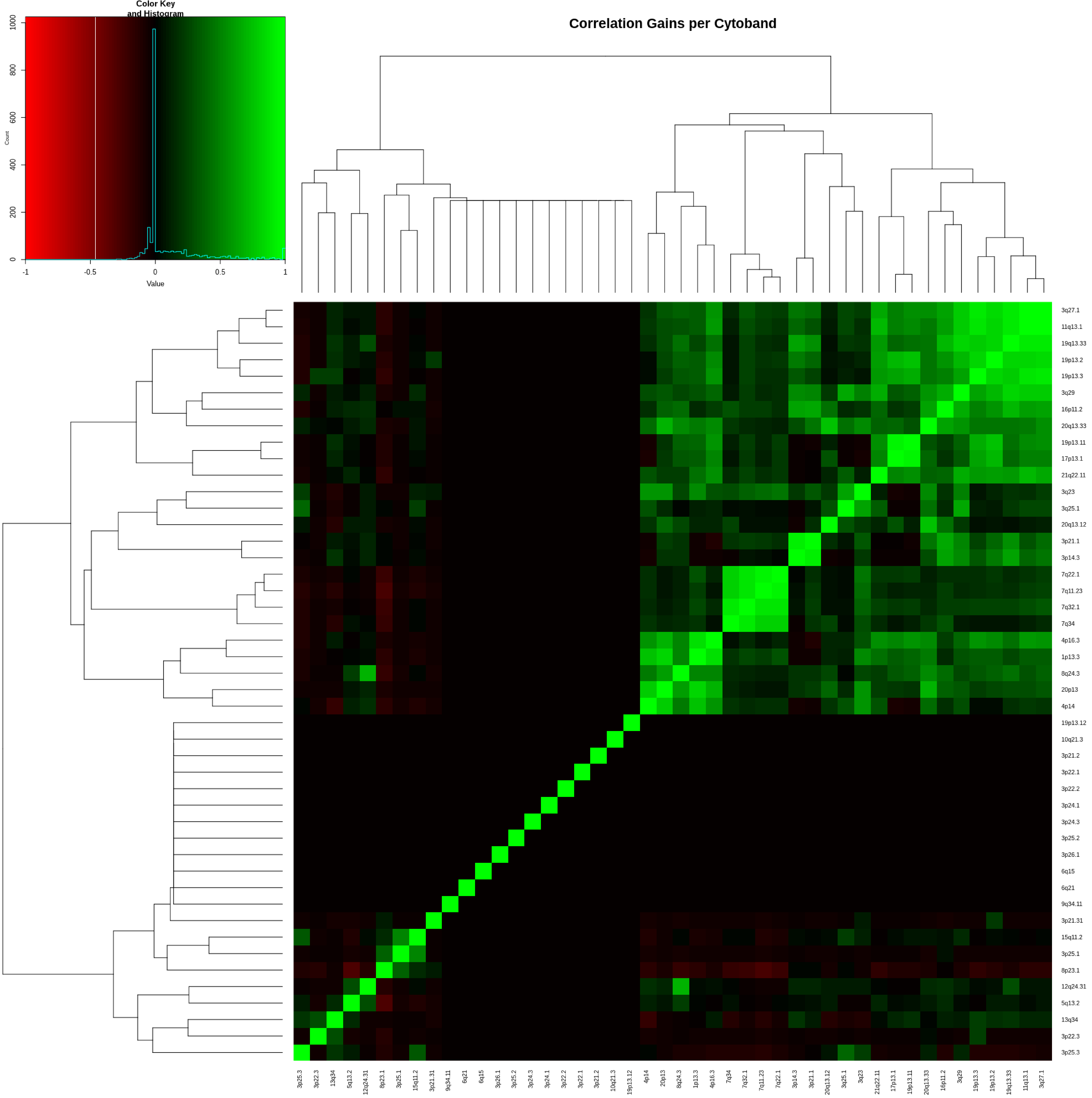
Color Key
and Histogram
Count
Value
Correlation Gains per Cytoband
3q27.1
11q13.1
19q13.33
19p13.2
19p13.3
3q29
16p11.2
20q13.33
19p13.11
17p13.1
21q22.11
3q23
3q25.1
20q13.12
3p21.1
3p14.3
7q22.1
7q11.23
7q32.1
7q34
4p16.3
1p13.3
8q24.3
20p13
4p14
19p13.12
10q21.3
3p21.2
3p22.1
3p22.2
3p24.1
3p24.3
3p25.2
3p26.1
6q15
6q21
9q34.11
3p21.31
15q11.2
3p25.1
8p23.1
12q24.31
5q13.2
13q34
3p22.3
3p25.3
