# Supplementary material for: Stratification of clear cell renal cell carcinoma (ccRCC) genomes by gene-directed copy number alteration (CNA) analysis
Source: PLoS One. 2017 May 9;12(5):e0176659. doi: 10.1371/journal.pone.0176659 (PMC5423597; doi:10.1371/journal.pone.0176659)
Supplement: S12 File — Decomposition of the KEGG Pathway PI3K representing tumour A: G1_365; B: G3_287 shows a colour coded map of genes, see as well Fig 7 of the manuscript. The shapes are: Red = LossBlue = GainYellow = Both states (Gain/Loss)Rectangle = Uncoloured, gene is not affected by copy number alterationRectangle = Grade (1/3) equally affected, colour shows aberrationCircle = Grade 3, colour shows aberrationDiamond = Grade 1, colour shows aberrationCorresponding data are enlisted at Table G in S2 File. (PDF) [file pone.0176659.s012.pdf]

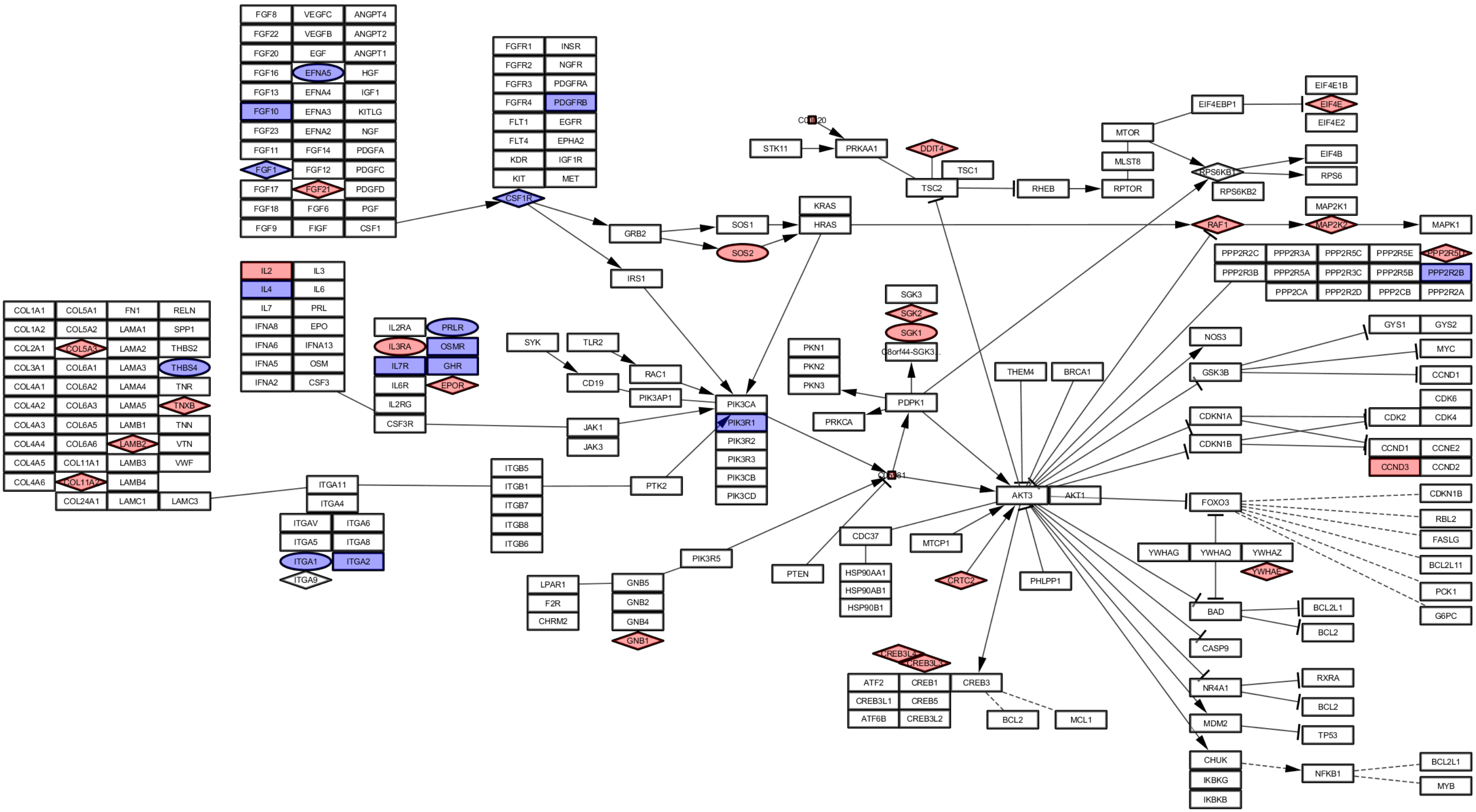
FGF8
VEGFC
ANGPT4
FGF22
VEGFB
ANGPT2
FGF20
EGF
ANGPT1
FGF16
EFNA5
HGF
FGF13
EFNA4
IGF1
FGF10
EFNA3
KITLG
FGF23
EFNA2
NGF
FGF11
FGF14
PDGFA
FGF1
FGF12
PDGFC
FGF17
FGF21
PDGFD
FGF18
FGF6
PGF
FGF9
FIGF
CSF1
FGFR1
INSR
FGFR2
NGFR
FGFR3
PDGFRA
FGFR4
PDGFRB
FLT1
EGFR
FLT4
EPHA2
KDR
IGF1R
KIT
MET
CSF1R
GRB2
SOS1
SOS2
KRAS
HRAS
IRS1
STK11
PRKAA1
DDIT4
TSC1
TSC2
RHEB
MTOR
MLST8
RPTOR
EIF4EBP1
EIF4E1B
EIF4E
EIF4E2
EIF4B
RPS6KB1
RPS6
RPS6KB2
MAP2K1
RAF1
MAP2K2
MAPK1
PPP2R2C
PPP2R3A
PPP2R5C
PPP2R5E
PPP2R5D
PPP2R3B
PPP2R5A
PPP2R3C
PPP2R5B
PPP2R2B
PPP2CA
PPP2R2D
PPP2CB
PPP2R2A
IL2
IL3
IL4
IL6
IL7
PRL
IFNA8
EPO
IFNA6
IFNA13
IFNA5
OSM
IFNA2
CSF3
IL2RA
PRLR
IL3RA
OSMR
IL7R
GHR
IL6R
EPOR
IL2RG
CSF3R
COL1A1
COL5A1
FN1
RELN
COL1A2
COL5A2
LAMA1
SPP1
COL2A1
COL5A3
LAMA2
THBS2
COL3A1
COL6A1
LAMA3
THBS4
COL4A1
COL6A2
LAMA4
TNR
COL4A2
COL6A3
LAMA5
TNXB
COL4A3
COL6A5
LAMB1
TNN
COL4A4
COL6A6
LAMB2
VTN
COL4A5
COL11A1
LAMB3
VWF
COL4A6
COL11A2
LAMB4
COL24A1
LAMC1
LAMC3
ITGA11
ITGA4
ITGAV
ITGA6
ITGA5
ITGA8
ITGA1
ITGA2
ITGA9
ITGB5
ITGB1
ITGB7
ITGB8
ITGB6
SYK
TLR2
RAC1
CD19
PIK3AP1
JAK1
JAK3
PTK2
PIK3CA
PIK3R1
PIK3R2
PIK3R3
PIK3CB
PIK3CD
PIK3R5
LPAR1
F2R
CHRM2
GNB5
GNB2
GNB4
GNB1
PTEN
CDC37
HSP90AA1
HSP90AB1
HSP90B1
SGK3
SGK2
SGK1
PKN1
PKN2
PKN3
PDPK1
PRKCA
THEM4
BRCA1
MTCP1
CRTC2
PHLPP1
AKT3
AKT1
CREB3L4
CREB3L3
ATF2
CREB1
CREB3
CREB3L1
CREB5
ATF6B
CREB3L2
BCL2
MCL1
NOS3
GSK3B
GYS1
GYS2
MYC
CCND1
CDKN1A
CDK6
CDK2
CDK4
CDKN1B
CCND1
CCNE2
CCND3
CCND2
FOXO3
CDKN1B
RBL2
FASLG
BCL2L11
PCK1
G6PC
YWHAG
YWHAQ
YWHAZ
YWHAE
BAD
BCL2L1
BCL2
CASP9
NR4A1
RXRA
BCL2
MDM2
TP53
CHUK
IKBKG
IKBKB
NFKB1
BCL2L1
MYB

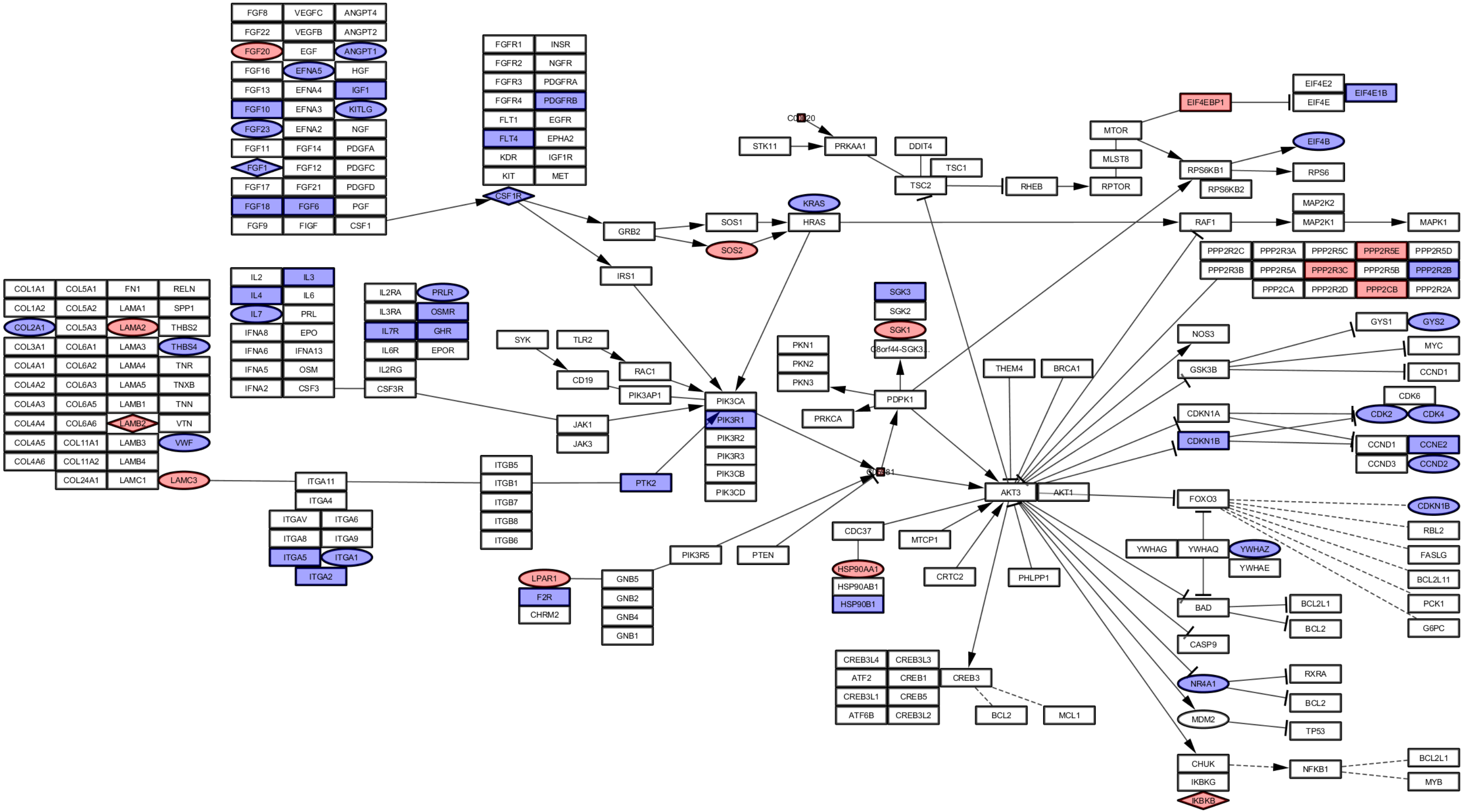

FGF8
VEGFC
ANGPT4
FGF22
VEGFB
ANGPT2
FGF20
EGF
ANGPT1
FGF16
EFNA5
HGF
FGF13
EFNA4
IGF1
FGF10
EFNA3
KITLG
FGF23
EFNA2
NGF
FGF11
FGF14
PDGFA
FGF1
FGF12
PDGFC
FGF17
FGF21
PDGFD
FGF18
FGF6
PGF
FGF9
FIGF
CSF1
FGFR1
INSR
FGFR2
NGFR
FGFR3
PDGFRA
FGFR4
PDGFRB
FLT1
EGFR
FLT4
EPHA2
KDR
IGF1R
KIT
MET
CSF1R
GRB2
SOS1
SOS2
KRAS
HRAS
IRS1
STK11
PRKAA1
DDIT4
TSC1
TSC2
RHEB
MTOR
MLST8
RPTOR
EIF4EBP1
EIF4E2
EIF4E
EIF4E1B
EIF4B
RPS6KB1
RPS6KB2
RPS6
RAF1
MAP2K2
MAP2K1
MAPK1
PPP2R2C
PPP2R3A
PPP2R5C
PPP2R5E
PPP2R5D
PPP2R3B
PPP2R5A
PPP2R3C
PPP2R5B
PPP2R2B
PPP2CA
PPP2R2D
PPP2CB
PPP2R2A
COL1A1
COL5A1
FN1
RELN
COL1A2
COL5A2
LAMA1
SPP1
COL2A1
COL5A3
LAMA2
THBS2
COL3A1
COL6A1
LAMA3
THBS4
COL4A1
COL6A2
LAMA4
TNR
COL4A2
COL6A3
LAMA5
TNXB
COL4A3
COL6A5
LAMB1
TNN
COL4A4
COL6A6
LAMB2
VTN
COL4A5
COL11A1
LAMB3
VWF
COL4A6
COL11A2
LAMB4
COL24A1
LAMC1
LAMC3
IL2
IL3
IL4
IL6
IL7
PRL
IFNA8
EPO
IFNA6
IFNA13
IFNA5
OSM
IFNA2
CSF3
IL2RA
PRLR
IL3RA
OSMR
IL7R
GHR
IL6R
EPOR
IL2RG
CSF3R
SYK
TLR2
CD19
RAC1
PIK3AP1
JAK1
JAK3
PIK3CA
PIK3R1
PIK3R2
PIK3R3
PIK3CB
PIK3CD
SGK3
SGK2
SGK1
C8orf44-SGK3...
PKN1
PKN2
PKN3
PDPK1
PRKCA
THEM4
BRCA1
AKT3
AKT1
NOS3
GSK3B
GYS1
GYS2
MYC
CCND1
CDK6
CDKN1A
CDK2
CDK4
CDKN1B
CCND1
CCNE2
CCND3
CCND2
FOXO3
CDKN1B
RBL2
FASLG
BCL2L11
PCK1
G6PC
YWHAG
YWHAQ
YWHAZ
YWHAE
BAD
BCL2L1
BCL2
CASP9
NR4A1
RXRA
BCL2
MDM2
TP53
CHUK
IKBKG
IKBKB
NFKB1
BCL2L1
MYB
ITGA11
ITGA4
ITGAV
ITGA6
ITGA8
ITGA9
ITGA5
ITGA1
ITGA2
ITGB5
ITGB1
ITGB7
ITGB8
ITGB6
PTK2
PIK3R5
PTEN
LPAR1
F2R
CHRM2
GNB5
GNB2
GNB4
GNB1
CDC37
HSP90AA1
HSP90AB1
HSP90B1
MTCP1
CRTC2
PHLPP1
CREB3L4
CREB3L3
ATF2
CREB1
CREB3
CREB3L1
CREB5
ATF6B
CREB3L2
BCL2
MCL1
