# Supplementary material for: Stratification of clear cell renal cell carcinoma (ccRCC) genomes by gene-directed copy number alteration (CNA) analysis
Source: PLoS One. 2017 May 9;12(5):e0176659. doi: 10.1371/journal.pone.0176659 (PMC5423597; doi:10.1371/journal.pone.0176659)
Supplement: S1 Text — CNA genes are described in Tables N and O in S2 File. CNA genes enlisted do not discriminate between Fuhrman G1 and G3 ccRCC tumours by Fisher’s exact testing. (DOCX) [file pone.0176659.s018.docx]

**Supporting text: Common CNAs**

The analysis resulted in 27 losses at 19 loci (see S2 Table 14) and 203 non-discriminatory CNA gene gains at 56 loci (see S2 Table 15). CNAs of *LINC00226*, of *ADAM6* and of *KIAA0125* result in gains in 45/48 HRO genomes at 14q32.33 displayed by 44 CNA genes, see S2 Table 15. Additional gene gains are found at chromosomal loci at 16p13.3 by 142/159 CNA genes and at 19p13.3 by 98/167 CNA genes. Common CNA loci were detected by *KIF9-AS1* in 46 HRO tumours at 3p21.31 (149 CNA genes) and in 45 tumours by *HERC2P3* at 15q11.2 (CNA 84 genes). Eighty-two cytoband loci shared by 20/48 ccRCC tumours outline the highest number of gene losses by 148/149 and 72/107 CNA genes at 3p21.31 and 16p11.2, respectively, followed by 68/96 CNA genes at 14q11.2, by 67/111 CNA genes at 19p13.2 and by 51/61 CNA genes at 14q24.3 (S5 Tables 1-3). The physical order of common ccRCC related gene alterations is visualized at loci 19p13.3 (167 genes / 98 gains / 47 losses / 22 mixed-type), 3p21.31 (149 genes / 0 gains / 148 losses / 1 mixed type) and on 16p13.3 (159 genes / 142 gains / 6 losses / 11 mixed type), see S4 Figure 1-2.
