# Supplementary material for: Stratification of clear cell renal cell carcinoma (ccRCC) genomes by gene-directed copy number alteration (CNA) analysis
Source: PLoS One. 2017 May 9;12(5):e0176659. doi: 10.1371/journal.pone.0176659 (PMC5423597; doi:10.1371/journal.pone.0176659)
Supplement: S2 Text — An extending detailed discussion of CNA gene set HRO 286 has been attached. (DOCX) [file pone.0176659.s019.docx]

**ccRCC tumour stratification by gene set HRO286**

In essence, a combination of common CNAs in concert with gene losses and gains at specific cytoband loci have shown in concordance with Fuhrman G1 or G3 gradings to be suitable for assigning CNA gene signatures to subsets of HRO ccRCC tumours (Fig 8). Combinations of 5 CNA gene sets enlisted as gene groups A, B, C, D, E and F encompassing 286 CNA genes from initially 15762 genes enabled the stratification of ccRCC subgroups by assigning malignancy states to individual ccRCC patients (S11 Table 7 & S10 Table 8). Fuhrman G1 grades seem to be associated with an increasing number of gene losses determined by Group A genes on 3p in conjunction with genes losses enlisted as Group B, Group D1 and Group E genes. Fuhrman grade G3 tumours are favoured by lower numbers of gene losses on 3p in combination with gene losses of Group C1 genes and gains of Group D2 genes (S10 Table 8). In cases ccRCC tumours are not classifiable by gene set HRO286, additional CNA gene sets might be determined accordingly to S10 Table 1 in order to support and enable alternative patient group classifications. For instance, G1 and G3 related gene sets specifying patients of patient group 2 (S10 Table 5, S10 Table 6) can be identified from 849 genes (S10 Table 1) leading to 28 CNA related genes (S10 Table 4) that distinguish 6 G1 ccRCC patients from 10 G3 ccRCC patients as visualized by correlation matrix analysis (S10 Table 5). In essence, correlation matrix analyses of this kind are a suitable tool for stratifying ccRCC patients from each other as well as for determining informative CNA gene subsets that jointly or differently distinguish individual ccRCC patient subgroups from each other. Thus, the workflow outlined in Fig 1 has been demonstrating its applicability for categorizing subgroups of ccRCC patients based on mutual functional or structural similarities that originate from mutual CNA signatures. Definitely, ccRCC genomes harbour recurrently occurring CNA signatures that enable sub-/stratifications of ccRCC patients. As such, copy number alterations either leading to G1 or G3 ccRCC tumours enlist distinct sets of CNA genes indicating that G3 tumour cells most likely do not directly evolve from established G1 tumour cells (S10 Table 8).
